# Synthesis of some novel coumarin-based heterocycles, elucidation of their antifungal behavior, molecular docking and computational studies

**DOI:** 10.1038/s41598-026-43854-5

**Published:** 2026-04-13

**Authors:** Mahmoud F. Ismail, Mounir A. I. Salem, Magda I. Marzouk, Naglaa F. H. Mahmoud, Nashwa H. Abdullah, Mustafa A. S. Gouda

**Affiliations:** 1 Department of Chemistry, Faculty of Science, Ain Shams University, Abbassia,Cairo, 11566 Egypt; 2 Botany and Microbiology Department, Faculty of Science, Capital University, Ain Helwan, Cairo, 11795 Egypt

**Keywords:** Cyanoacetohydrazide, Coumarin, Fused coumarin, Antifungal activity, Computational study, Biochemistry, Chemistry, Computational biology and bioinformatics, Drug discovery

## Abstract

A novel series of coumarin–cyanoacetohydrazide hybrids was synthesized via efficient strategies that exploit the nucleophilic and electrophilic functionalities of the cyanoacetohydrazide scaffold. The designed framework incorporated a C-4 amino-substituted coumarin nucleus, enabling the construction of diverse fused and heteroannulated coumarin derivatives under mild conditions, affording moderate to excellent yields. The structures of all new compounds were confirmed by spectroscopic analyses (IR, MS, ^1^H, and ^13^C NMR). Selected derivatives were evaluated for antifungal activity against phytopathogenic fungi. Results revealed that compounds **2**, **3**, **6**, **7**, and **8** exhibited notable inhibition, while compound **8** showed the broadest antifungal spectrum and the highest inhibition potency (inhibition zones up to 4.00 ± 0.50 mm). IC_50_ determination highlighted compounds **2** (0.35 mg/mL) and **3** (< 0.63 mg/mL) as the most potent. At the same time, molecular docking against five key fungal enzymes indicated strong, multi-target binding affinities for compounds 6–8, particularly 8, which showed ΔG values below -8.982 kcal/mol. Density Functional Theory (DFT) calculations established correlations between frontier orbital energies, energy gaps, softness, dipole moments, and antifungal performance, suggesting that reduced ΔE and enhanced softness favor the biological activity. These findings identify compound **8** as a promising broad-spectrum antifungal lead and provide valuable structure–activity insights for the rational design of coumarin-based antifungal agents.

**Supplementary Information:**

The online version contains supplementary material available at 10.1038/s41598-026-43854-5.

## Introduction

**Fig. 1:**
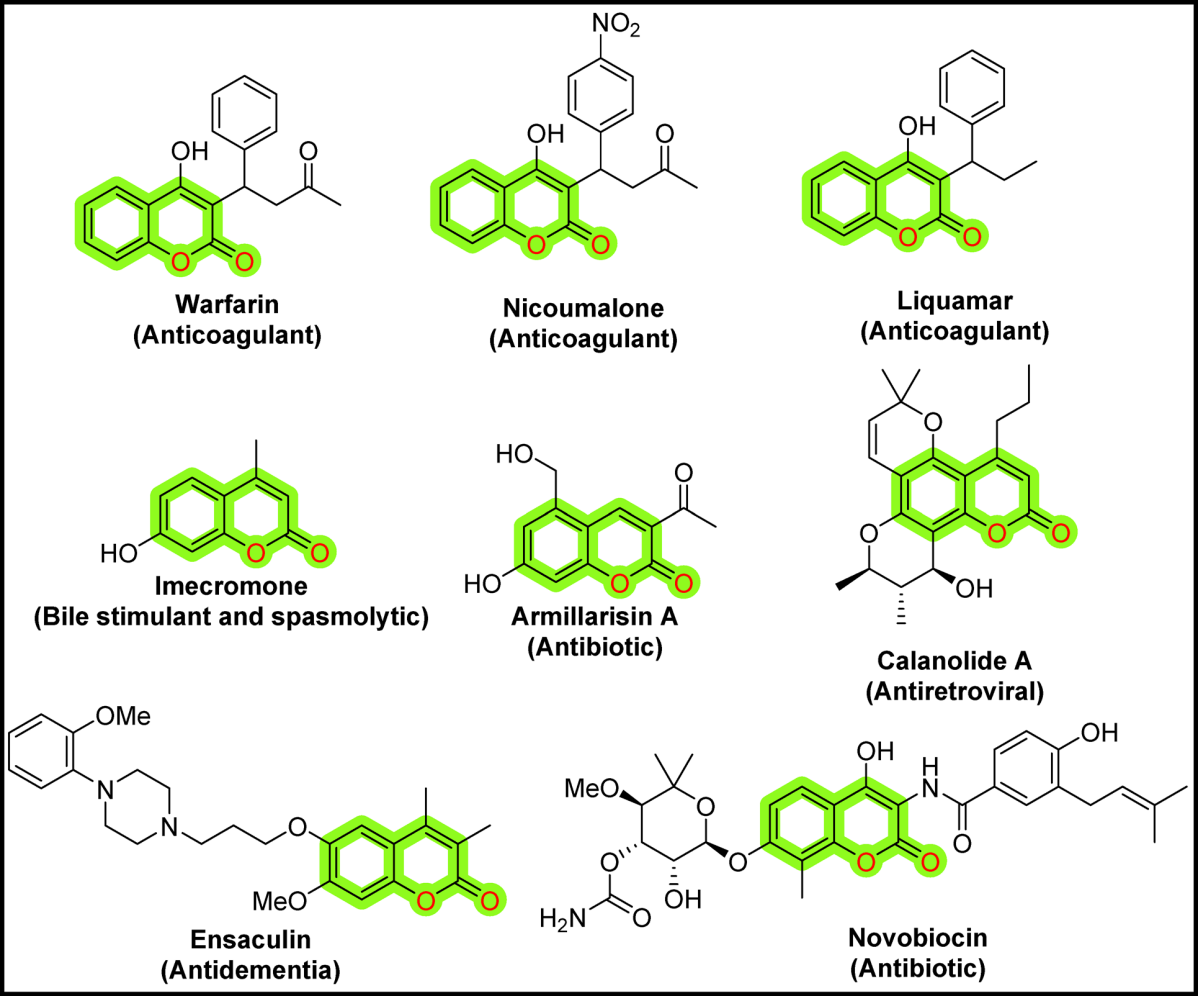
Structures of some marketed drugs containing a coumarin nucleus.

Coumarin is a unique chemical scaffold that occurs naturally in a wide variety of plants. Notably, over 1,300 naturally occurring coumarins have demonstrated promising biomedical properties and have been identified in a variety of plants, bacteria, and fungi^1^. Figure 1 illustrates the contribution of coumarin-based compounds to several marketed drugs, including Warfarin, Nicoumalone, Liquamar, Imecromone, Armillarisin A, Calanolide A, Ensaculin, and Novobiocin.

**Fig. 2:**
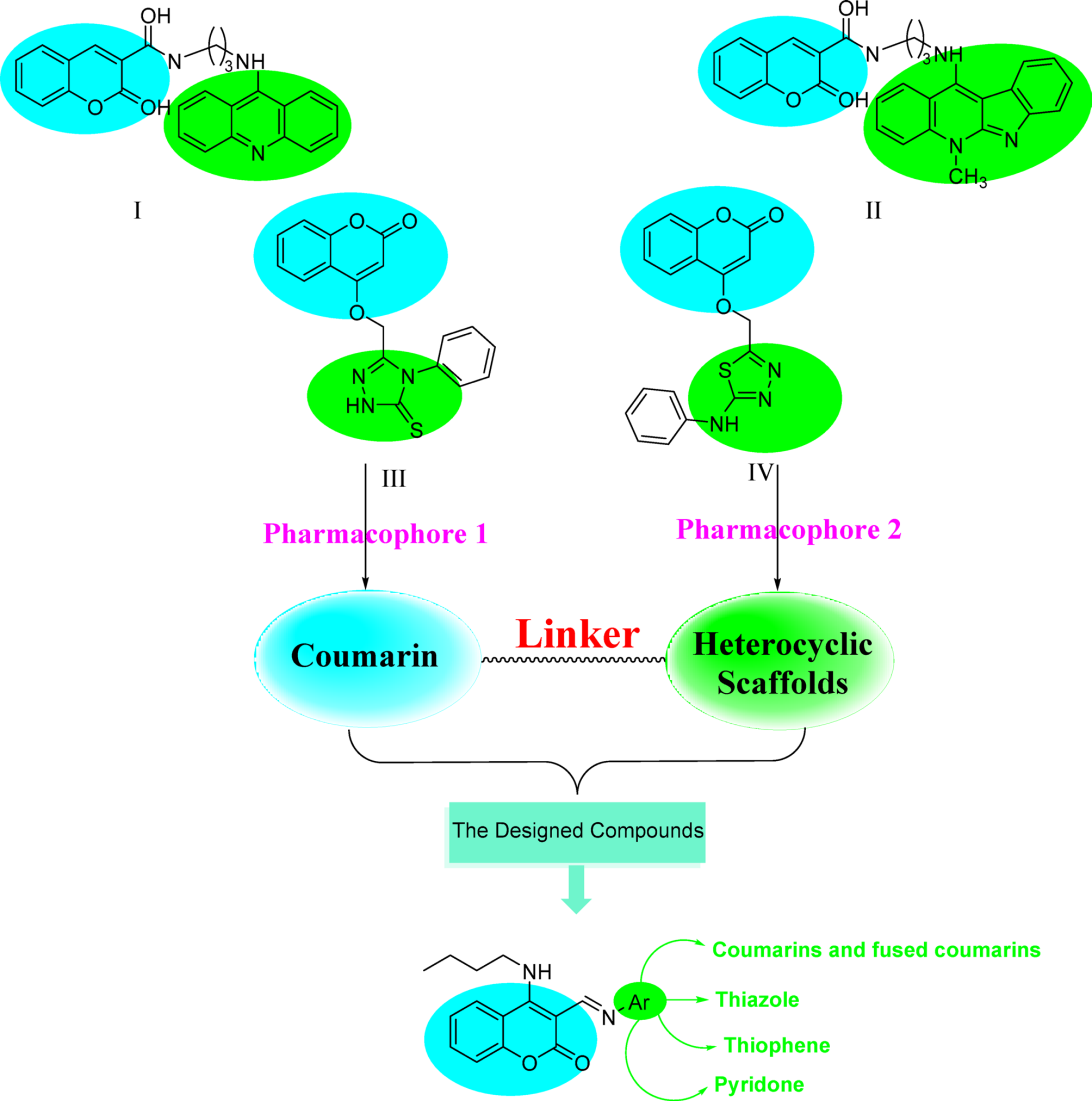
illustrates the suggested design rationale of the target compounds.

Recently^2,3^, coumarins have unquestionably garnered significant attention owing to their remarkable chemical diversity and wide-ranging pharmacological properties, such as, anti-inflammatory^4^, antioxidant^5^, antibacterial^6^, antifungal^7^, anticancer^8^, anticoagulant^9^, antitubercular^10^, anticonvulsant^11^, antiviral^12^, antihypertensive^13^, anti-HIV^14^, antidiabetic^15^ activities, and also coumarins have a fascinating range of applications in cosmetic^16^, perfumery^17^, food additives^18^, and optical brighteners^19^. Additionally, coumarins exhibit excellent photophysical behaviors^20,21^ and are widely used as fluorescent probes for sensing metal ions and proteins^22,23^. (Fig. 2)

On the other hand, cyanoacetohydrazide serves as a versatile and valuable scaffold in the synthesis of diverse heterocyclic compounds incorporating one or more heteroatoms, involving mixed and non-mixed systems such as thiophene, pyridone, coumarin, iminocoumarin, thiazole, thiadiazole, and pyrazole rings^24–29^. This broad synthetic utility stems from the presence of multiple functional groups within its structure, offering both electrophilic (C≡N group) and nucleophilic (NH group and the active methylene group in the presence of a base) centers. These reactive sites can be selectively or simultaneously exploited to form targeted heterocyclic frameworks with desired structural and pharmacological properties.

Herein, we aim to develop novel coumarin-based heterocyclic compounds with remarkable antifungal activity against fungal plant pathogens. To achieve this, we synthesized and employed *N*’-((4-(butylamino)-2-oxo-2*H*-chromen-3-yl)methylene)-2-cyanoacetohydrazide **(4)** as a versatile scaffold for constructing diverse heterocyclic moieties hybrid with coumarin ring, to obtain promising antifungal candidates, potentially valuable for drug discovery and therapeutic applications.

### Design rationale

Several coumarin derivatives have been extensively reported for their antifungal and antimicrobial potential through diverse mechanisms, with the notable advantage of structural versatility, metabolic stability, and the ability to form strong hydrogen-bonding and π–π interactions with microbial enzymes^2,30^. The benzopyrone nucleus of coumarin is considered a privileged pharmacophore due to its planar aromatic system, which facilitates stacking interactions within enzyme active sites, in addition to the lactone carbonyl group that functions as a hydrogen bond acceptor^31,32^.

Furthermore, heterocyclic systems are well established as key structural motifs in drug discovery^33^. Nitrogen- and sulfur-containing heterocycles have been shown to enhance biological activity by introducing additional hydrogen bond donors and acceptors, modulating electronic distribution, and improving molecular rigidity^34,35^.

For instance, substituted coumarin analogues have demonstrated promising antifungal activity against Candida species with MIC values in the low micromolar range, in some cases approaching the activity of reference azole drugs^36^. Similarly, coumarin–heterocycle hybrids were reported to exhibit enhanced antimicrobial activity through improved electronic modulation and additional hydrogen bonding interactions within target enzymes. Furthermore, samples (**I)** and (**II)** considered as highly efficient treatment against both *C. albicans* sample^37^. Moreover, In vitro antifungal screening effects of the compounds (**III)** and (**IV)**, were tested against some fungal spices (Aspergillus niger and Candida albicans). Compounds (**III)** and (**IV)** showed good activities as antifungals compared to the antifungal ability of fluconazole, which was used as a standard^38^. These findings emphasize the pharmacological significance of the coumarin when linked to heterocyclic scaffold as a core structure for the development of novel antifungal agents.

Based on these considerations, the present study was designed to develop novel coumarin-based heterocyclic derivatives by integrating key pharmacophoric features within a single molecular framework. The coumarin nucleus was retained as the main chromophore responsible for π–π and hydrophobic interactions, while diverse heterocyclic moieties were introduced to enhance hydrogen bonding capability and electronic modulation. Structural rigidification through cyclization was employed to improve shape complementarity within the biological target. These findings collectively underscore the rationale for designing coumarin-based heterocyclic hybrids as potential antifungal agents.

## Results and discussion

### Chemistry

**Scheme 1:**
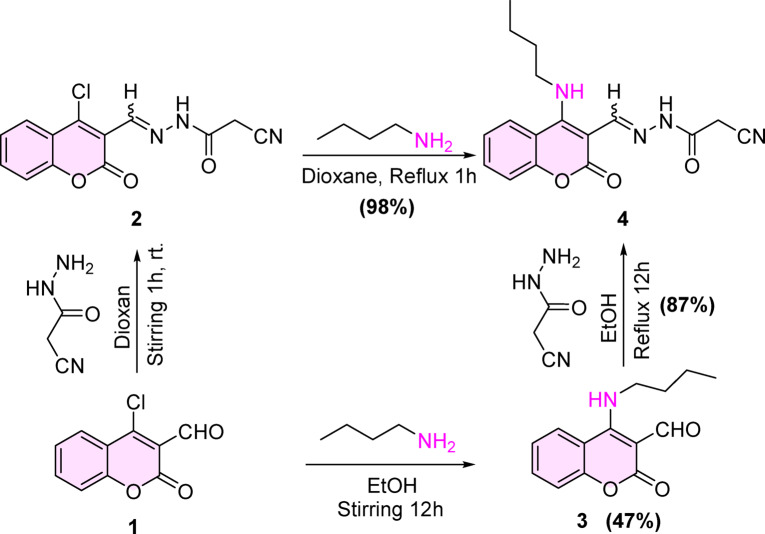
synthesis of the titled compound 4.

At the outset, we discuss the strategy for the synthesis of the newly formed compound **4** to give a better yield under smooth conditions. *N*’-((4-(butylamino)-2-oxo-2*H*-chromen-3-yl)methylene)-2-cyanoacetohydrazide **(4)** was emanated from refluxing for an hour of *N*’-((4-chloro-2-oxo-2*H*-chromen-3-yl)methylene)-2-cyanoacetohydrazide **(2)**^39^ with *n*-butyl amine, compound **2** was prepared through the reaction of 4-chloro-2-oxo-2*H*-chromene-3-carbaldehyde **(1)**^40^ with cyanoacetohydrazide^41^ according to our previous work^39^. On the other hand, refluxing compound **1** with *n*-butyl amine for 12 h gave 4-(butylamino)-2-oxo-2*H*-chromene-3-carbaldehyde **(3)** in a moderate yield (47%), followed by condensation of the later with cyanoacetohydrazide also afforded the desirable compound **4** in yield (87%) after longer time (12 h) compared with the first strategy the yield was an excellent (98%) after 2 h. **(**Scheme 1**).**

The formation of compound **4** was established by the appearance of four peaks in the ^1^H NMR spectrum: three multiplet peaks and one triplet peak corresponding to the *n*-butyl moiety at 3.88–3.87, 1.73–1.72, 1.47–1.40, 0.90 ppm, respectively. Moreover, two NH, CH=N and CH_2_CN protons manifested at (11.58, 11.72), (9.49, 10.89), (8.43, 8.51) and (4.12, 3.75) ppm corresponding to *syn*- and *anti*-isomers, respectively, due to the restricted rotation about the C=N bond.

**Scheme 2:**
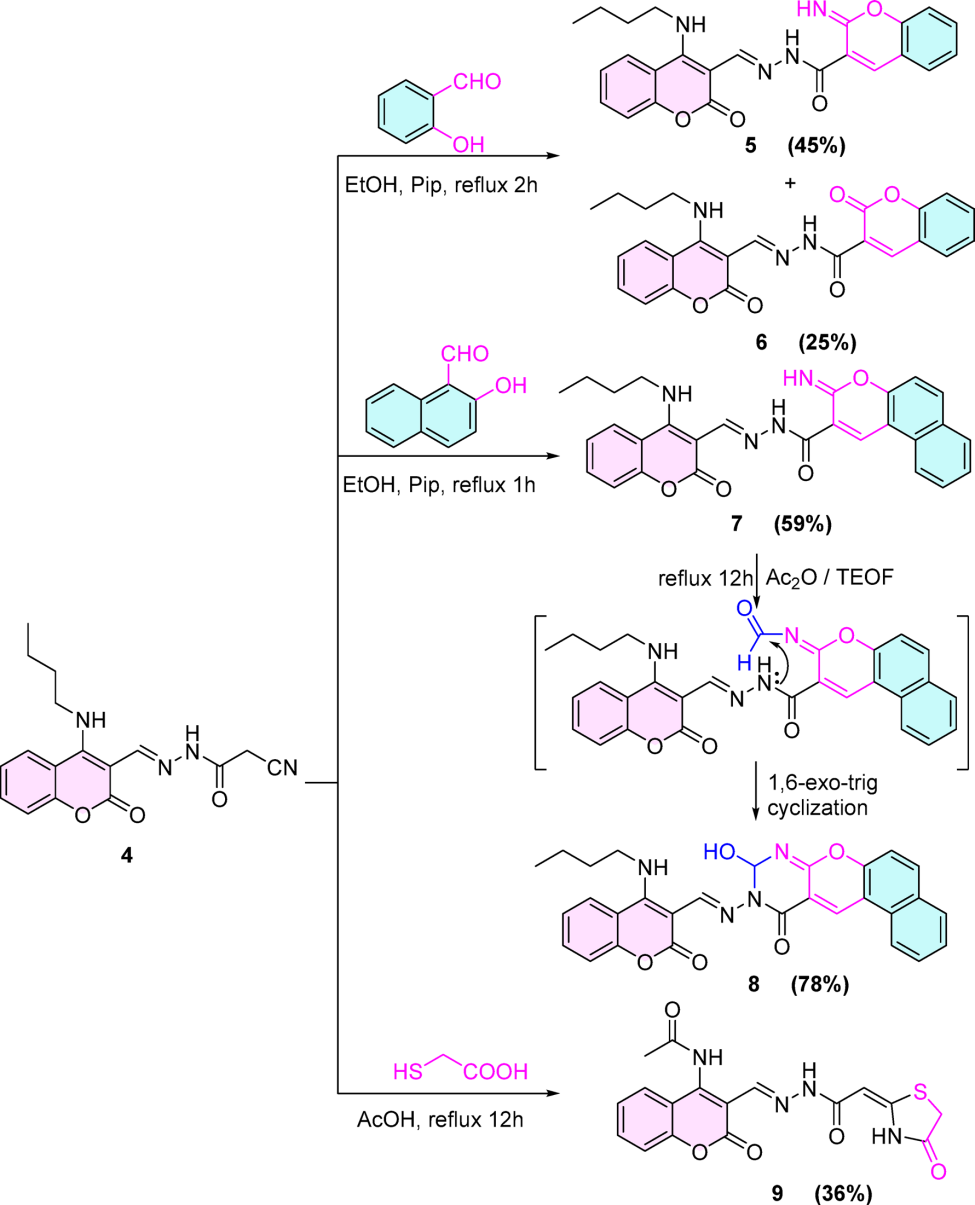
synthesis of compounds 5–9.

After protection of the position C-4 in the coumarin ring by *n*-butyl amino group, the reactivity of the functionalities included in cyanoacetohydrazide was estimated by different reagents such as *o*-salicylaldehyde, thioglycolic acid, acetylacetone, ethyl acetoacetate, carbon disulfide, and arylidene derivatives. For instance, refluxing compound **4** with *o*-salicylaldehyde in an ethanolic solution in the presence of a secondary amine (piperidine) under Knoevenagel condensation conditions afforded iminocoumarin derivative **5** in 45% yield via a 1,6-exo-dig cyclization. In the same manner, a part of **5** undergoes hydrolysis and affords coumarin derivative **6** in 25% yield (see Supplementary Materials, Scheme 1). **(**Scheme 2**)**. The foreseeable chemical structures of compounds **5** and **6** were confirmed by spectroscopic data, where the ^1^H NMR spectrum of compound **4** displayed two singlet peaks at 8.75 and 8.11 ppm compatible with C_4_-H_(iminocoumarin)_ and NH (exchangeable with D_2_O) protons. Meanwhile, compound **5** revealed a singlet peak at 8.84 corresponding to C_4_-H_(coumarin)_.

Also, the reaction of **4** with 2-hydroxynaphthaldehyde under the same conditions proceeded uneventfully to afford the 3-iminobenzo[*f*]chromene derivative **7** in a reasonable yield (59%). Afterwards, heating compound **7** with a mixture of triethyl orthoformate (TEOF) and acetic anhydride furnished benzo[5,6]chromeno[2,3-*d*]pyrimidone derivative **8** in (78%). (Scheme 2).

The ^1^H NMR spectrum of compound **7** manifested three broad singlet peaks exchangeable with D_2_O at 13.58, 12.63, 11.01 ppm corresponding to three NH protons, two singlet peaks at 9.11 and 8.42 ppm corresponding to C_1_-H_(3-iminobenzochromene)_ and CH=N protons, four doublet, a triplet and a multiplet peaks in aromatic region (8.47–7.29) belong to ten aromatic protons beside four peaks in shielding region according to *n*-butyl protons. On the other hand, the 1H NMR spectrum of compound 8 showed only two broad singlet peaks exchangeable with D_2_O at 10.83 and 5.13 ppm compatible with NH and OH protons, and a singlet peak at 5.74 ppm consistent with the behavior of C_2_-H_(pyrimidinone)_ proton.

Furthermore, the reactivity of the cyano group of cyanoacetohydrazide **4** was estimated via the reaction of compound **4** with thioglycolic acid, which gave 1,3-thiazolone derivative **9** in a low yield (36%). (Scheme 2) The IR spectrum elucidated the formation of 1,3-thiazolone moiety by the appearance of a stretching absorption band at 1740 cm^-1^ corresponding to C=O of 1,3-thiazolidin-4-one moiety beside that, the ^1^H NMR spectrum revealed a peak at 2.05 ppm corresponding to CH_3_ protons of the acetyl group and disappearance of the peaks of *n*-butyl group which indicated that the *n*-butyl group was broken, followed by acetylation by acetic acid.

**Scheme 3:**
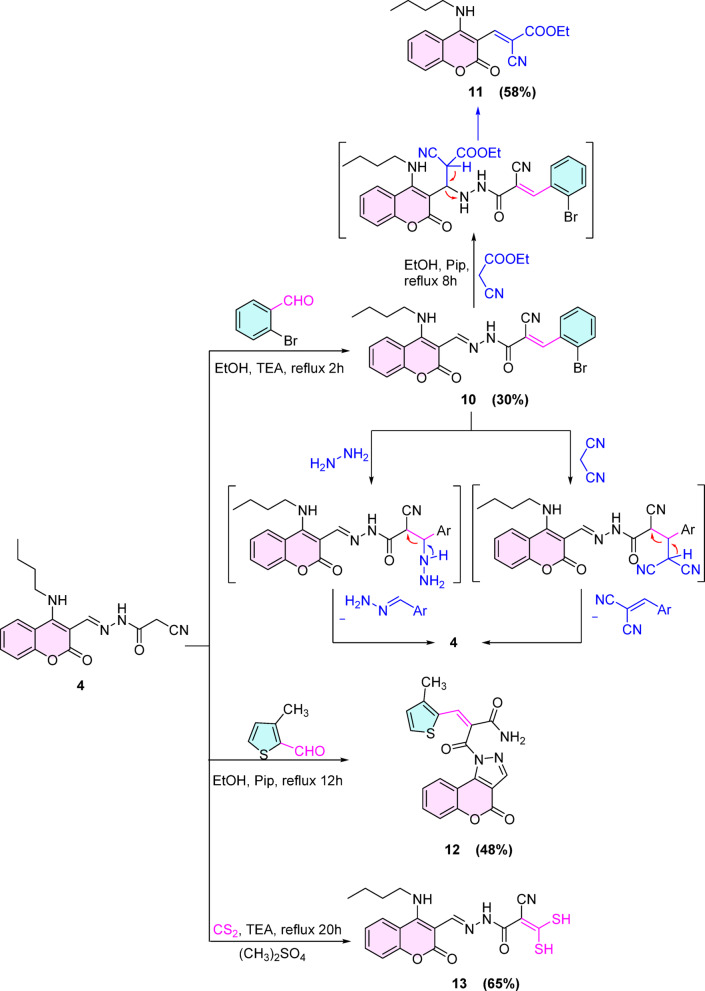
synthesis of compounds 10–13.

Reaction of compound **4** with 2-bromobenzaldehyde in ethanol containing a catalytic amount of triethylamine (TEA) afforded the arylidene derivative **10** as dark yellow crystals in a low yield (30%). (Scheme 3) The construction of arylidene derivative **10** was interpreted by the spectral data, such as the IR spectrum, which displayed a band at 2217 cm^-1^ corresponding to the conjugated C≡N group, and the ^1^H NMR spectrum showed a singlet peak at 8.40 ppm corresponding to the CH_(olefinic)_ proton.

Unfortunately, when compound **10** reacted individually with malononitrile and hydrazine hydrate, arylidene malononitrile and hydrazone derivatives were expelled and gave the starting compound **4**, respectively. Whereas, in the case of refluxing compound 10 with ethyl cyanoacetate, it gave coumarinoarylidene ethyl cyanoacetate derivative **11**, namely ethyl-3-(4-(butylamino)-2-oxo-2*H*-chromen-3-yl)-2-cyanoacrylate **(11)**. (Scheme 3).

Meanwhile, condensation of 3-methylthiophene-2-carbaldehyde with cyanoacetohydrazide derivative **4** at the active methylene group gave compound **12**. (Scheme 3) Actually, the formation of the unexpected compound **12** (see Supplementary Materials, Scheme 2) was interesting to interpret this structure, where the IR spectrum disappeared the cyano band, at the same time, the ^1^H NMR spectrum also disappeared the peaks of *n*-butyl protons and showed two broad singlet peaks at 7.25, 6.91 ppm corresponding to NH_2_ protons, two doublet peaks at 7.08, 6.69 ppm corresponding to two thiophene protons with *J* = 5.1 Hz, respectively, and a singlet peak at 2.09 corresponding to CH_3_ protons.

Moreover, the reaction of **4** with carbon disulfide furnished *N*’-((4-(butylamino)-2-oxo-2*H*-chromen-3-yl)methylene)-2-cyano-3,3-dimercaptoacrylohydrazide **(13)** in a reasonable yield (65%). (Scheme 3).

**Scheme 4:**
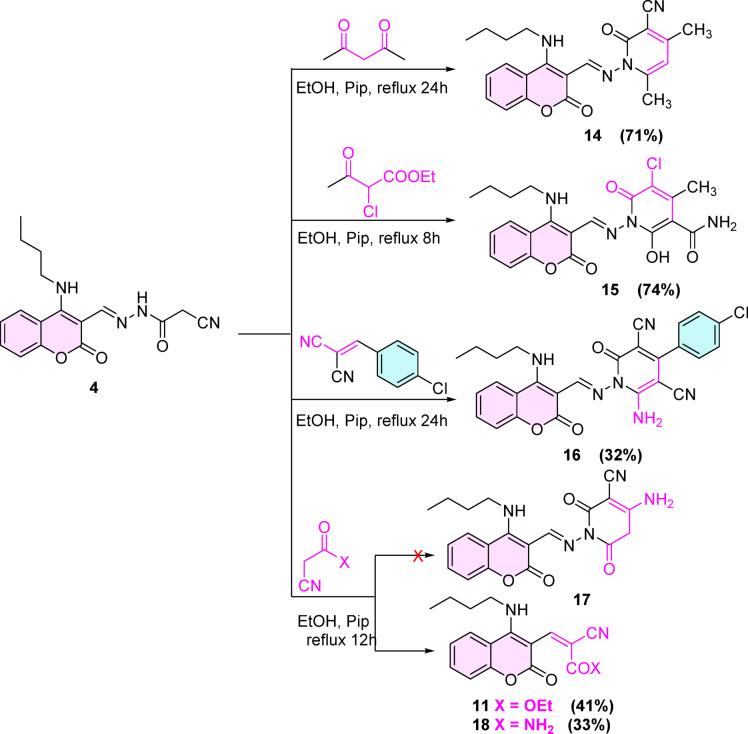
synthesis of compounds 13–17.

On the other hand, the reactivity of the active methylene encouraged us to estimate its reactivity beside the nucleophilicity of NH motif to construct variant pyridone derivatives. For instance, the reaction of coumarin cyanoacetohydrazone derivative **4** individually with acetyl acetone, ethyl-2-chloro-3-oxobutanoate, and 4-chlorobenzylidene malononitrile in ethanol containing drops of secondary amine (piperidine) afforded the predictable pyridone derivatives **14**–**16** uneventfully, respectively. (Scheme 4).

The chemical structures of compounds **14**–**16** were unambiguously ascertained by different spectral tools, such as the IR spectrum of compound **14**, which displayed bands at 2213, 1708, and 1651 cm^-1^ characteristic for C≡N, C = O_(coumarin)_, and C = O_(pyridone)_ functionalities. Furthermore, the ^1^H-NMR exhibited only one exchangeable peak at 10.65 ppm corresponding to NH proton and two singlet peaks at 6.39 and 2.34 ppm compatible with C_5_-H_(pyridone)_ and two CH_3_ protons.

Unfortunately, ethyl 3-(4-(butylamino)-2-oxo-2*H*-chromen-3-yl)-2-cyanoacrylate **18** commenced instead of pyridine derivative **17** when compound **4** reacted with ethyl cyanoacetate or cyanoacetamide under the same conditions. (Scheme 4).

### Biological activity

#### Antifungal activity

**I- Screening:**

The initial qualitative screening of antifungal activities showed that compounds **2**,** 3**,** 6**,** 7,** and **8** showed a detectable antifungal activity against at least one of the tested fungal pathogens Table 1.

**Table 1:** Screening of antifungal activities of synthesized compounds against fungal plant pathogens.

Pathogen Compounds	Inhibition Zone diameter (mm) “Mean ± standard deviation”				
	*Fusarium solani*	*Fusarium oxysporum*	*Fusarium semitectum*	*Alternaria solani*	*Rhizoctonia solani*
2	ND	ND	1.25 ± 0.25	ND	ND
3	ND	1.75 ± 0.25	ND	ND	2.75 ± 0.00
6	ND	0.75 ± 0.05	ND	ND	1.50 ± 0.00
7	ND	2.50 ± 0.00	2.00 ± 0.00	ND	ND
8	ND	2.05 ± 0.05	2.75 ± 0.25	3.25 ± 0.25	4.00 ± 0.50

*: Not Detected.

**II- Estimation of IC**_**50**_ **:**

Based on the previous screening results, compounds **2**, **3**, **6**, **7**, and **8** were selected to estimate their IC_50_ against the most inhibited fungal pathogen.

**Fig. 3:**
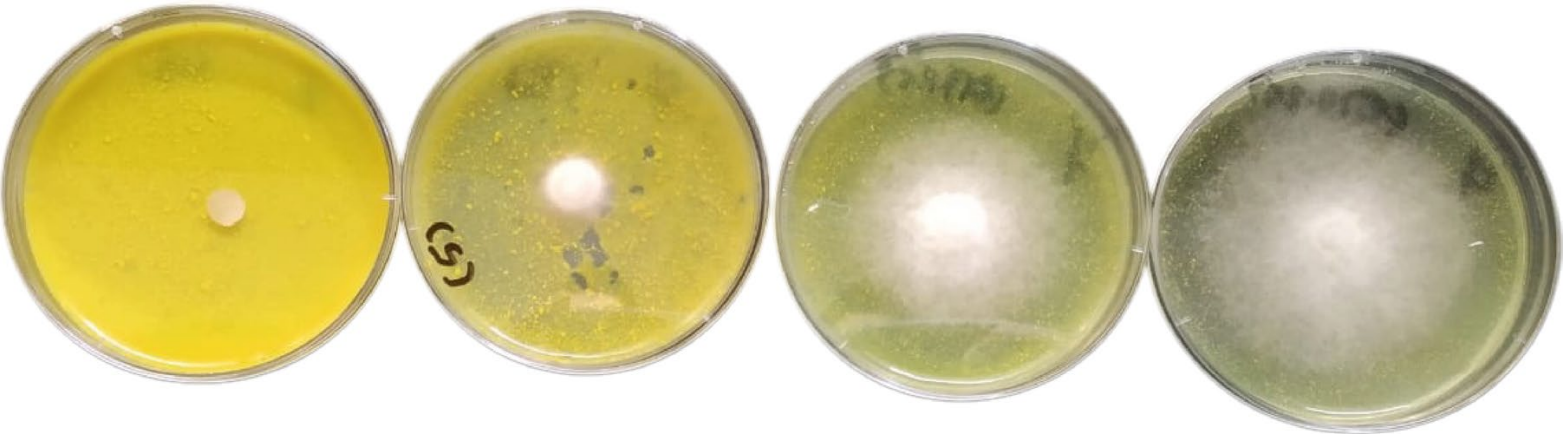
Dose dependent inhibition of *Rhizoctonia solani* by compound 8.

**Fig. 4:**
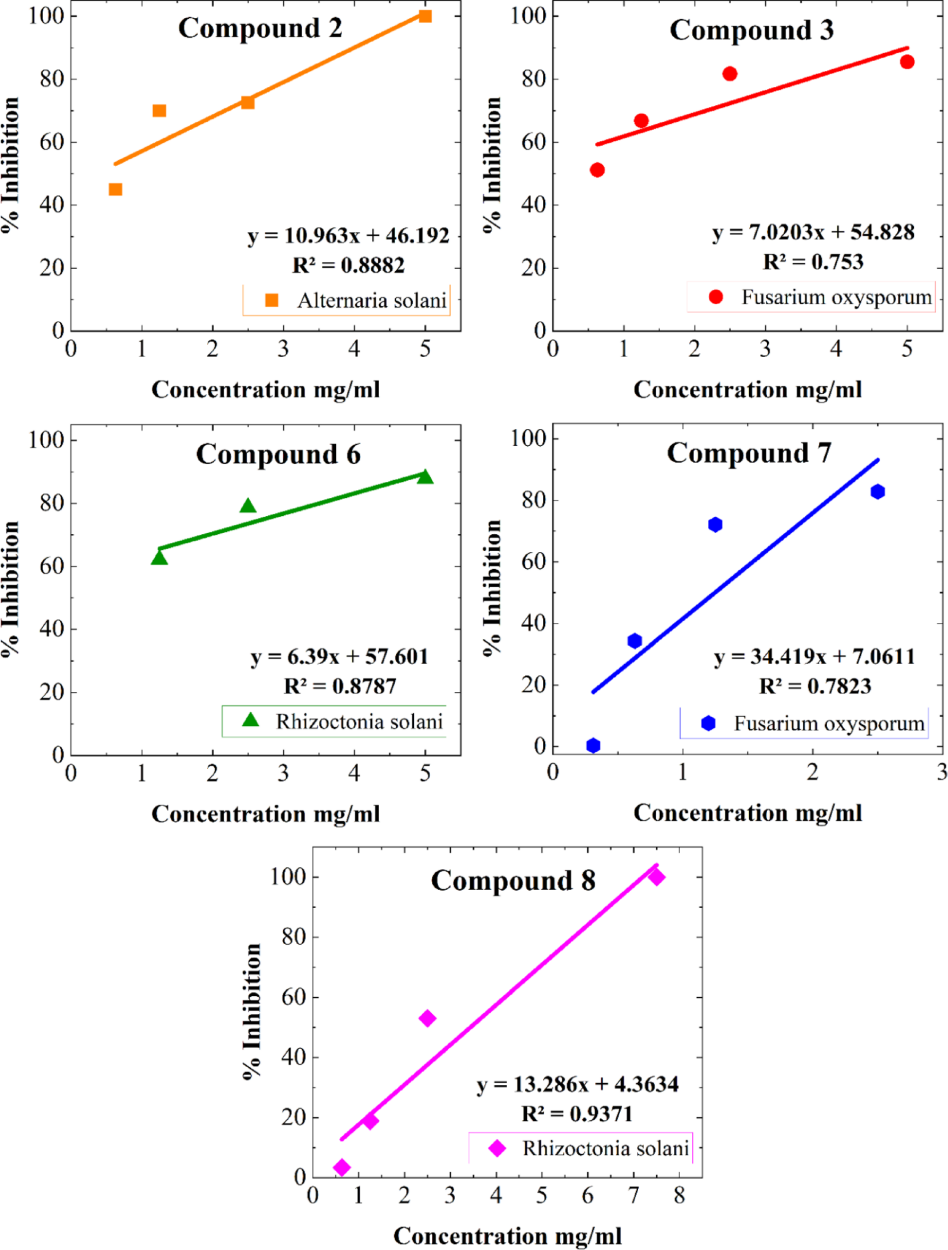
Dose response curves for compounds 2, 3, 6, 7 and 8.

Results show that compounds **2** and **3** have recorded the best IC_50_ values among the tested compounds, recording IC_50_ values at 0.35 and < 0.63 mg/mL concentration, respectively (Table 2, 3 and Fig. 3, 4). On the other hand, compounds **2** and **8** have exerted 100% growth inhibition against the tested fungi at **5** and 7.5 mg/mL concentrations respectively while compounds **7**, **3** and **6** have inhibited the fungal growth with 82.8–87.8% at 2.5, 5 and 5 mg/mL concentrations respectively which reflects their promising antifungal activities. Coumarins (1,2-benzopyrones or 2*H*-1-benzopyran2-ones) are well-characterized bioactive benzopyrone compounds. Their biological activity is basically correlated to their ability to noncovalently interact with different active sites in targeted living cells. As some coumarin derivatives were proven to inhibit fungal growth and exert low cytotoxicity, these compounds were strongly recommended as promising anti-fungal agents in many fields, including medicine, the food industry, and the agriculture sectors^42^. (Table 4).

**Table 2:** Response of *Alternaria solani* for doses of Compound 2.

Tested concentration mg/mL	% Growth inhibition Mean value ± standard error	MIC	IC_50_	
Compound 2	0.63	45.0 ± 0.50	5 mg/mL	0.35 mg/mL
	1.25	70.0 ± 5.7		
	2.5	72.6 ± 0.87		
	5	100 ± 0.0		
Fluconazole	0.1	77.54 ± 0.36	0.4 mg/mL	< 0.1 mg/mL
	0.2	83.33 ± 0.36		
	0.4	100 ± 0.0		
	0.8	100 ± 0.0

**Table 3:** Response of *Fusarium oxysporum* for doses of Compounds 3 & 7.

Tested concentration mg/mL	% Growth inhibition Mean value ± standard error		MIC	IC_50_
Compound 3	0.63	51.19 ± 1.3	> 5 mg/mL	~ 0.63 mg/mL
	1.25	66.79 ± 0.83		
	2.5	81.68 ± 0.0		
	5	85.50 ± 0.38		
Compound 7	0.31	0.36 ± 2.0	> 2.5 mg/mL	1.24 mg/mL
	0.63	34.35 ± 0.76		
	1.25	72.14 ± 0.63		
	2.5	82.82 ± 0.33		
Fluconazole	0.1	85.03 ± 0.27	0.8 mg/mL	< 0.1 mg/mL
	0.2	86.11 ± 0.25		
	0.4	86.77 ± 0.31		
	0.8	100 ± 0.0

**Table 4:** Response of *Rhizoctonia solani* for doses of Compounds 6 & 8.

Tested concentration mg/mL	% Growth inhibition		MIC	IC50
	Mean value ± standard error			
Compound 6	1.25	62.16 ± 1.7	> 5 mg/mL	< 1.25 mg/mL
	2.5	78.72 ± 0.56		
	5	87.84 ± 0.0		
Compound 8	0.63	3.38 ± 1.8	7.5 mg/mL	3.43 mg/mL
	1.25	18.92 ± 0.0		
	2.5	53 ± 2.3		
	7.5	100 ± 0.0		
Fluconazole	0.1	82.41 ± 0.23	0.8 mg/mL	< 0.1 mg/mL
	0.2	85.80 ± 0.28		
	0.4	88.61 ± 0.0		
	0.8	100 ± 0.0		

**Fig. 5:**
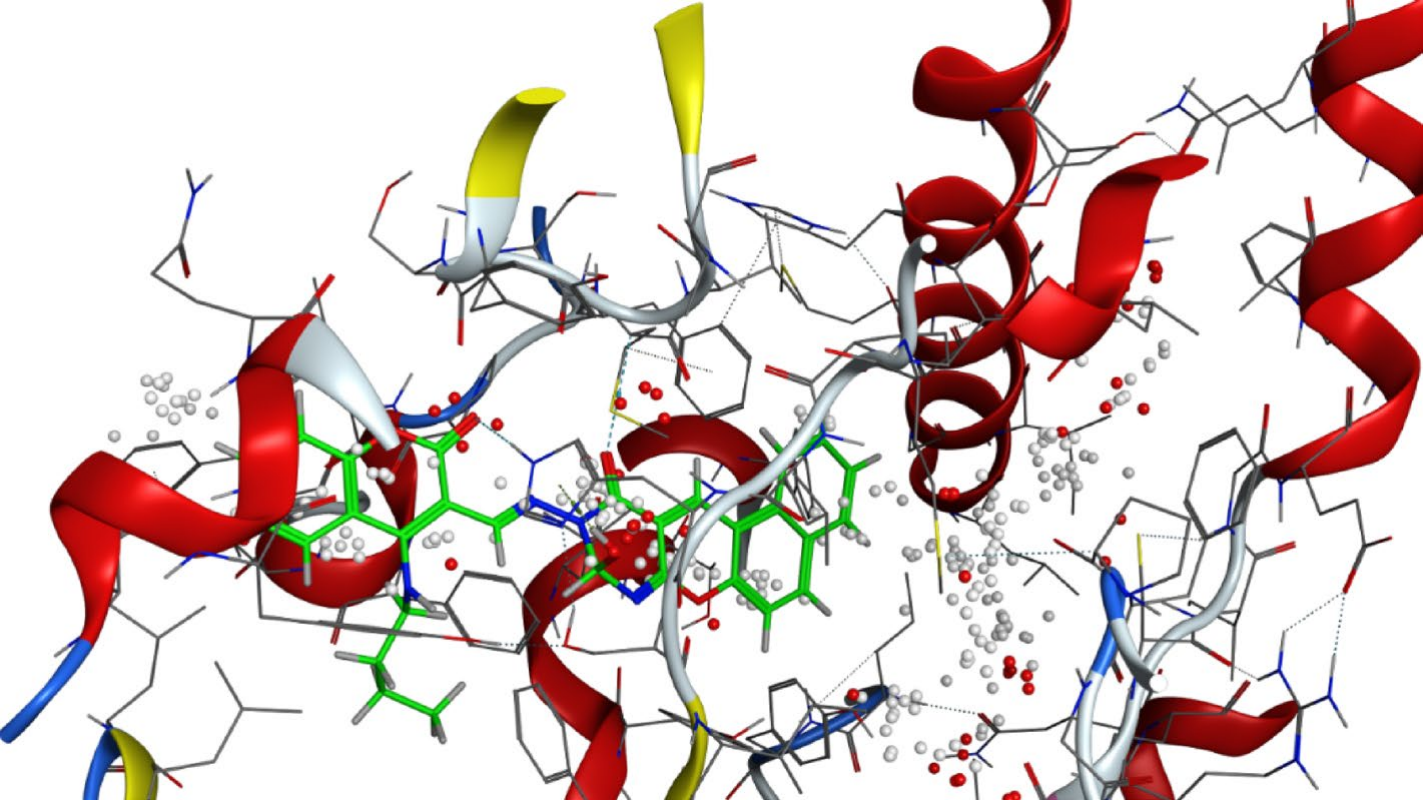
3D binding mode of compound 8 with Sterol 14-alpha demethylase (PDB: 5tz1) active site of the highest binding scores.

Many studies have reported the potential of coumarin compounds to suppress the growth of different fungal pathogens, including soil-borne, plant, and human fungal pathogens such as *Candida. albicans, C. tropicalis*, A. flavus^43^**,** *Aspergillus fumigatus*^43,44^, *Valsa. mali*^45^, *Alternaria alternata*^46^, *Botrytis cinerea* and *Rhizoctonia solani*^47^. (Fig. 5)

**Fig. 6:**
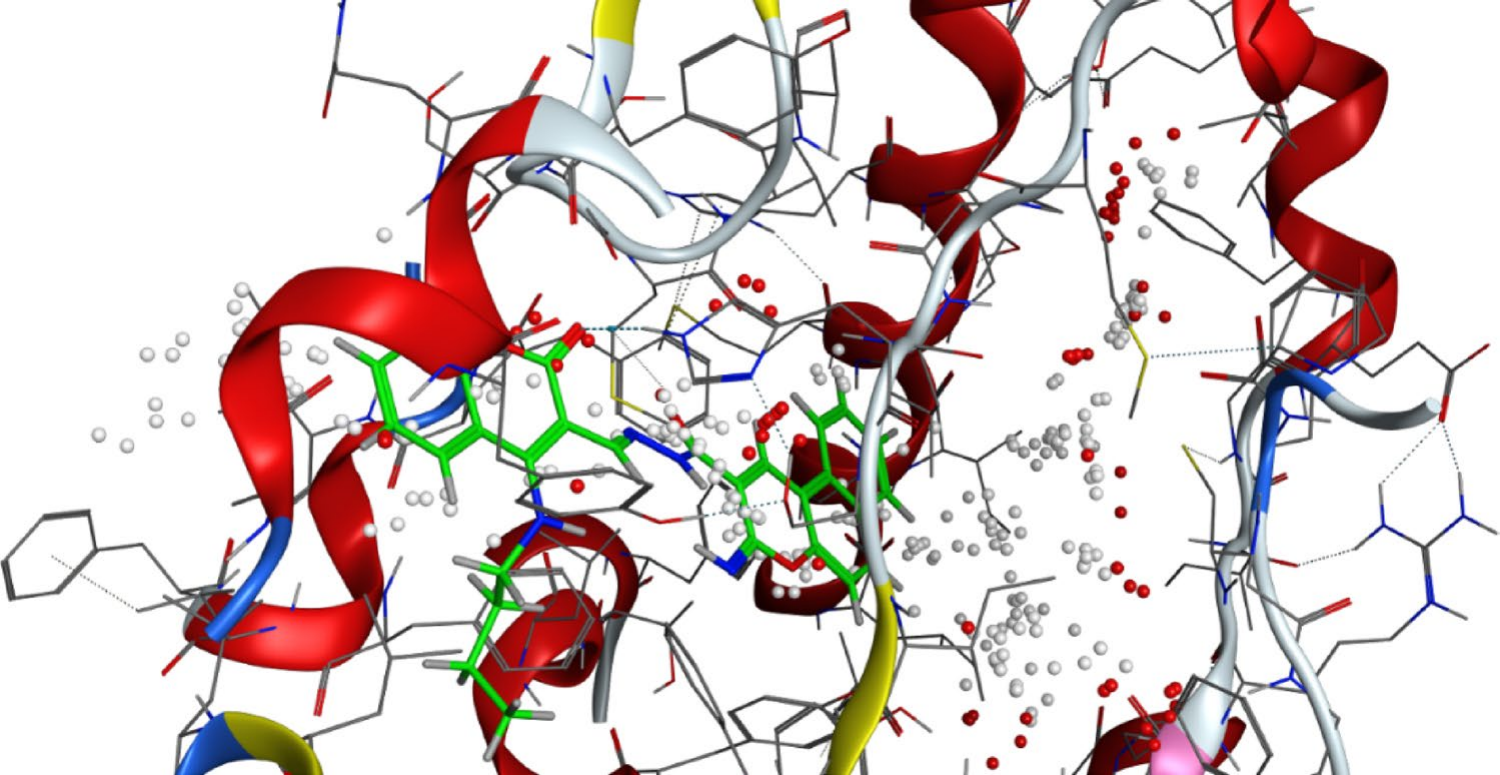
3D binding mode of compound 7 with Sterol 14-alpha demethylase (PDB: 5tz1) active site of the highest binding scores.

**Fig. 7:**
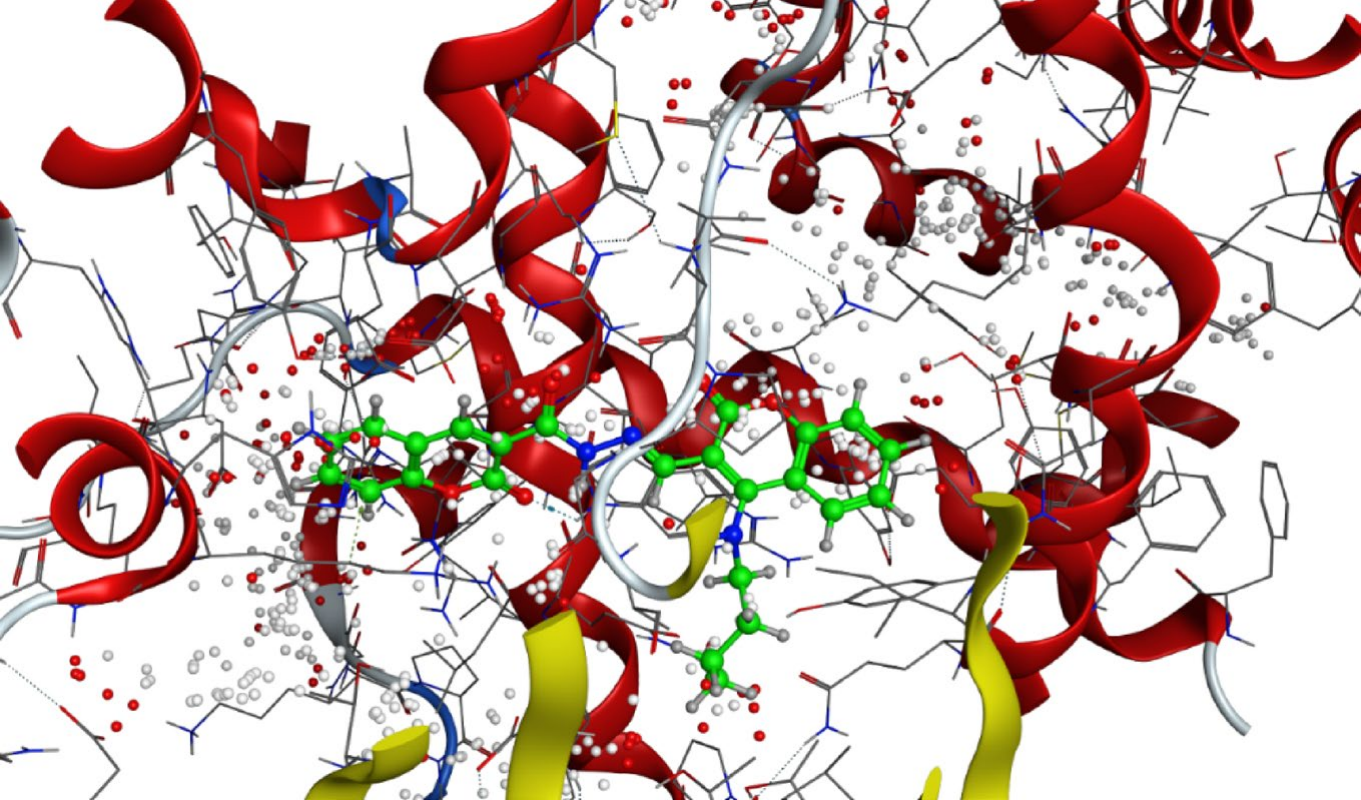
3D binding mode of compound 6 with Chitin synthase (PDB: 7stl) active site of the highest binding scores.

It is well-documented that the antifungal activity of coumarins is strongly structure-dependent^42^**.** Among a series of 8-substituted coumarin derivatives, 8-chloro coumarin and ethyl 8-chloro-coumarin**-**3-carboxylate showed the strongest antifungal activities against important plant fungal pathogens: *Botrytis cinerea*, *Colletotrichum gloeosporioides*, *Fusarium oxysporum*, and *Valsa mali*^48^. Also, among 39 novelly synthesized 3-phenylhydrazone coumarin compounds, 4-chloro-3-phenylhydrazone-coumarin derivative was reported to exert the most potent antifungal effect against Colletotrichum orbiculare and Rhizoctonia solani^47^. Al-Amiery et al.^38^ have detected the inhibition activity of Coumarins 4-((5-mercapto-4-phenyl-4*H*-1,2,4-triazol-3-yl)-methoxy)-2*H*-chromen-2-one and 4-((5-(phenylamino)-1,3,4-thiadiazol-2-yl)-methoxy)-2*H*-chromen-2-one against *Aspergillus niger* and *Candida albicans*. Also, Betti et al*.*^49^ have reported the potential of 2-(coumarin-4-yloxy) acetohydrazid*e* to inhibit these fungi. (Figs. 6 and 7)

The mechanisms underlying the coumarin’s antifungal activity were estimated to involve cell membrane damage possibly^45,47,49^, disruption of cellular functions via induced oxidative stress^49^**,** and disruption of mitochondrial functions^47^. Zhang et al*.*^46^ Have confirmed the induction of obvious structural damage in the treated fungi, resulting in a depletion of the cellular contents. Additionally, they have detected the effect of a coumarin derivative on the pentose phosphate pathway, causing mitochondrial destruction.

#### Molecular docking studies

To gain deeper insight into the antifungal potential and probable mechanism of action of the synthesized compounds, molecular docking studies were conducted against five critical fungal target proteins: EXO-B-(1,3)-GLUCANASE (PDB ID: 1cz1), topoisomerase II-DNA-nucleotide (4GFH), Dihydrofolate Reductase (4h95), Sterol 14-alpha demethylase (5tz1), and Chitin synthase (7stl). These enzymes are key regulators of fungal cell wall integrity and sterol biosynthesis and represent validated targets for antifungal therapy^50–54^.

The docking results, summarized in Table 5, revealed that compounds **6, 7**, and** 8** demonstrated the most favorable binding energies with all five proteins with small rmsd_.

**Table 5:** Docking binding free energies (DG) of the synthesized candidates with five critical fungal target proteins.

Compound	Protein									
	1cz1		4GFH		4h95		5tz1		7stl	
	Docking score ∆G (kcal/mol)	rmsd_ refine	Docking score ∆G (kcal/mol)	rmsd_ refine	Docking score ∆G (kcal/mol)	rmsd_ refine	Docking score ∆G (kcal/mol)	rmsd_ refine	Docking score ∆G (kcal/mol)	rmsd_ refine
2	-6.056	1.119	-5.611	1.397	-5.942	0.8196	-6.247	1.144	-6.388	0.9662
3	-5.798	1.527	-5.363	1.397	-6.087	1.851	-6.265	1.068	-6.174	1.935
6	-7.35	1.525	-6.95	1.622	-7.542	1.235	-8.068	1.687	-8.236	1.276
7	-7.511	1.73	-7.033	1.461	-8.072	1.555	-8.596	1.563	-8.371	1.411
8	-7.853	1.281	-7.802	1.447	-8.197	1.653	-8.982	1.746	-8.757	1.472

refine which represents the root mean square deviation between the refined docking pose of the ligand and the reference binding pose, and is used as an indicator of the reliability and consistency of the predicted binding orientation. Compound** 8** consistently exhibited the strongest binding scores, ranging from − 7.80 to − 8.98 kcal/mol. Notably, compound **8** showed the highest binding affinity with Sterol 14-alpha demethylase (5tz1, − 8.982 kcal/mol) and Chitin synthase (7stl, − 8.757 kcal/mol), indicating strong and stable interactions at their active sites. These in silico results confirm the in vitro antifungal results, in which compound 8 indeed displayed the broader range of activity among the tested derivatives. It exerted an inhibitory effect against four fungal pathogens, namely *Fusarium oxysporum (2.05* ± *0.05 mm)*, *Fusarium semitectum (2.75* ± *0.25 mm)*, *Alternaria solani (3.25* ± *0.25 mm)*, and *Rhizoctonia solani (4.00* ± *0.50 mm)*. The strong correlation between the estimated docking scores and the obtained antifungal activities suggests that compound **8** acts via effective and possibly multi-target inhibition mechanisms. However, the relatively higher IC_50_ value (3.43 mg/mL) suggests that despite strong binding affinity, higher concentrations are needed to achieve effective in vitro inhibition, possibly due to cell penetration or solubility limitations. (Fig. 8)

**Fig. 8:**
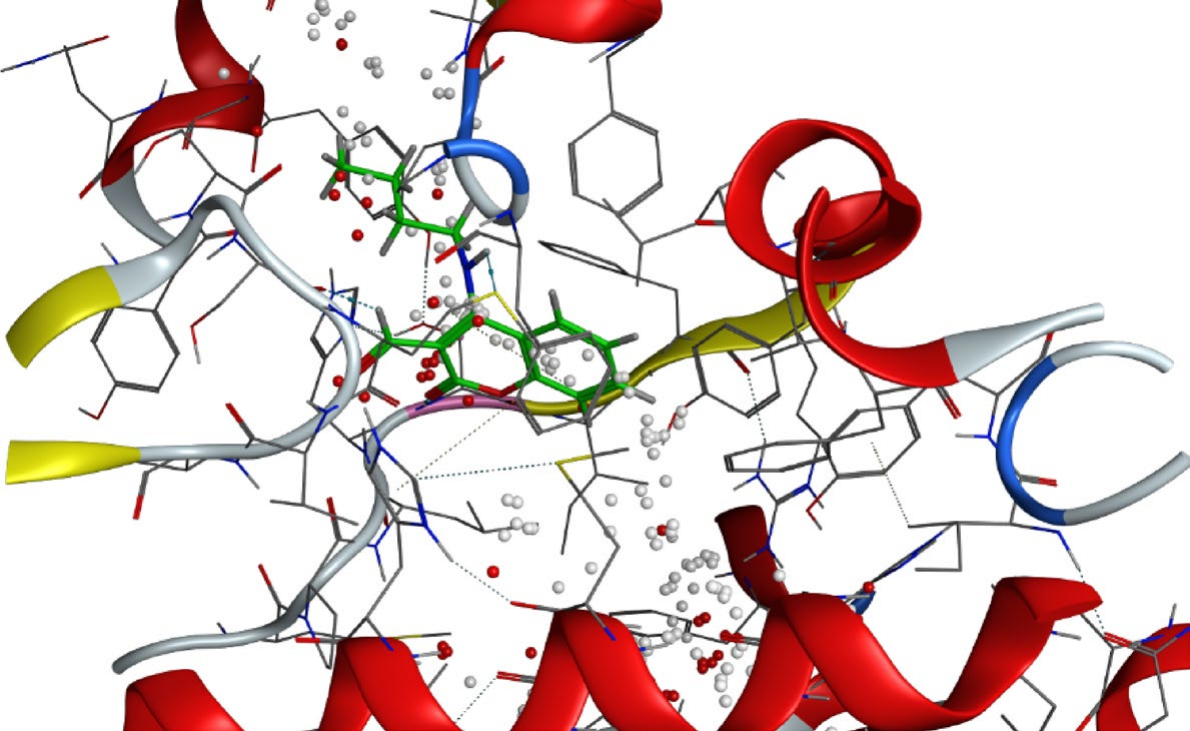
3D binding mode of compound 3 with Sterol 14-alpha demethylase (PDB: 5tz1) active site of the highest binding scores.

**Fig. 9:**
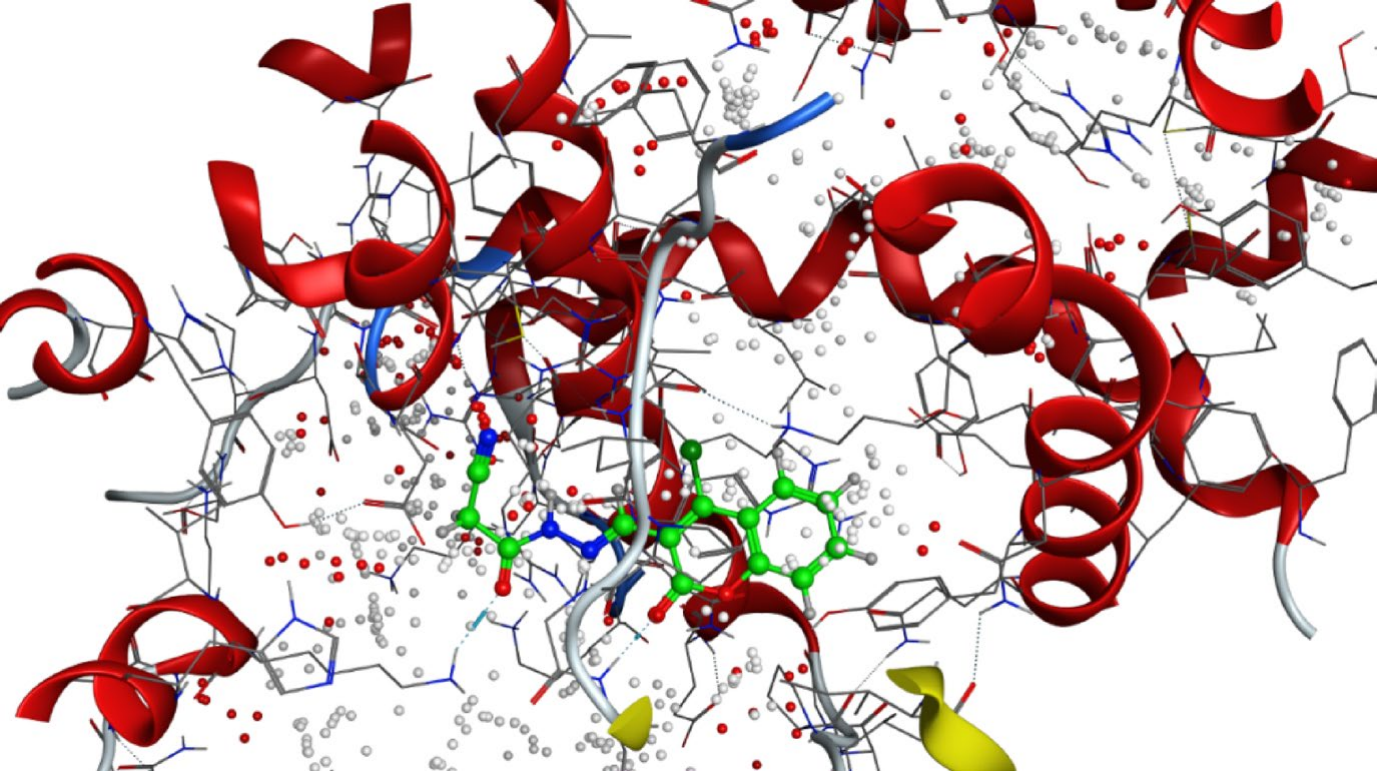
3D binding mode of compound 2 with Chitin synthase (PDB: 7stl) active site of the highest binding scores.

Similarly, compound** 7** exhibited promising docking scores, particularly with 5tz1 (− 8.596 kcal/mol) and 4h95 (− 8.072 kcal/mol), which correspond well with its inhibitory effect against *F. oxysporum (2.50* ± *0.00 mm)* and *F. semitectum (2.00* ± *0.00 mm)*. Its activity against these fungal pathogens and its favorable docking scores imply that compound **7** likely exerts antifungal activity due to its potential to interact with multiple vital fungal pathways. (Fig. 9)

Compound** 6** showed slightly lower docking scores than **7** and **8** (e.g., -8.236 kcal/mol with 7stl). Indeed, it showed a lower antifungal activity in the in vitro study. It exerted a lower inhibitory effect against *F. oxysporum* and *R. solani*, with inhibition zones of 0.75 ± 0.05 mm and 1.50 ± 0.00 mm, respectively. The consistency of these results strongly validates the predictive power of the performed docking studies. It highlights the contribution of specific structural features such as hydrogen bond donors and hydrophobic substituents in enhancing protein–ligand binding.

Interestingly, although compounds** 2** and** 3** showed only modest docking scores (e.g., − 6.056 to − 6.388 kcal/mol for compound** 2**) compared to compound **6**, they exhibited better biological activity, particularly compound** 3**, which showed inhibition against *F. oxysporum (1.75* ± *0.25 mm)* and *R. solani (2.75* ± *0.00 mm)*. These discrepancies between docking and inhibition zone results may be attributed to factors not fully captured by rigid docking simulations, such as passive diffusion through fungal membranes, compound solubility, or interactions with secondary targets.

Overall, these findings suggest that compound** 8** represents the most promising broad-spectrum antifungal candidate based on both computational and biological evaluations, and compound** 7** displays a strong potential as a multi-target inhibitor.

#### Density functional theory (DFT) studies

To elucidate the structural basis behind the observed antifungal activities, quantum chemical descriptors were calculated using Density Functional Theory (DFT) at the B3LYP/6-31G(d,p) level for compounds **2**, **3**, **6**, **7**, and **8**. The obtained quantum chemical parameters, including frontier molecular orbital energies (**HOMO** and **LUMO**), energy gap (**Δ*E***), ionization potential (***I***), electron affinity (**A**), chemical hardness (***η***), electronegativity (***χ***), chemical softness (***S***), electronic chemical potential (***P***), electrophilicity index (**ω**), and dipole moment (**µ**), are presented in Table 6.

**Table 6:** Quantum chemical parameters of the selected compounds with Density Functional Theory (DFT) at B3LYP/6-31G (d,p) basis set.

Compound	2	3	6	7	8
*E* HOMO (eV)	-6.9146	-6.8248	-6.1078	-5.6692	-6.0387
*E* LUMO (eV)	-3.2376	-2.6193	-2.4359	-2.2103	-2.7243
(*∆E*) Energy gap (eV)	3.6770	4.2055	3.6718	3.4588	3.3143
(*I*) Ionization energy (eV)	6.9146	6.8248	6.1078	5.6692	6.0387
(A) Electron affinity (eV)	3.2376	2.6193	2.4359	2.2103	2.7243
(ƞ) Chemical hardness (eV)	1.8385	2.1027	1.8359	1.7294	1.6571
(*χ*) Electronegativity (eV)	5.0761	4.7221	4.2719	3.9397	4.3815
(*S*) Chemical softness (eV^-1^)	0.5439	0.4755	0.5446	0.5782	0.6034
(*P*) Electronic chemical potential (eV)	-5.0761	-4.7221	-4.2719	-3.9397	-4.3815
(ω) Electrophilicity index (eV)	7.0075	5.3021	4.9699	4.4876	5.7924
(µ) Dipole moment (D)	12.0080	14.2160	14.7365	15.0472	8.3959

**Fig. 10:**
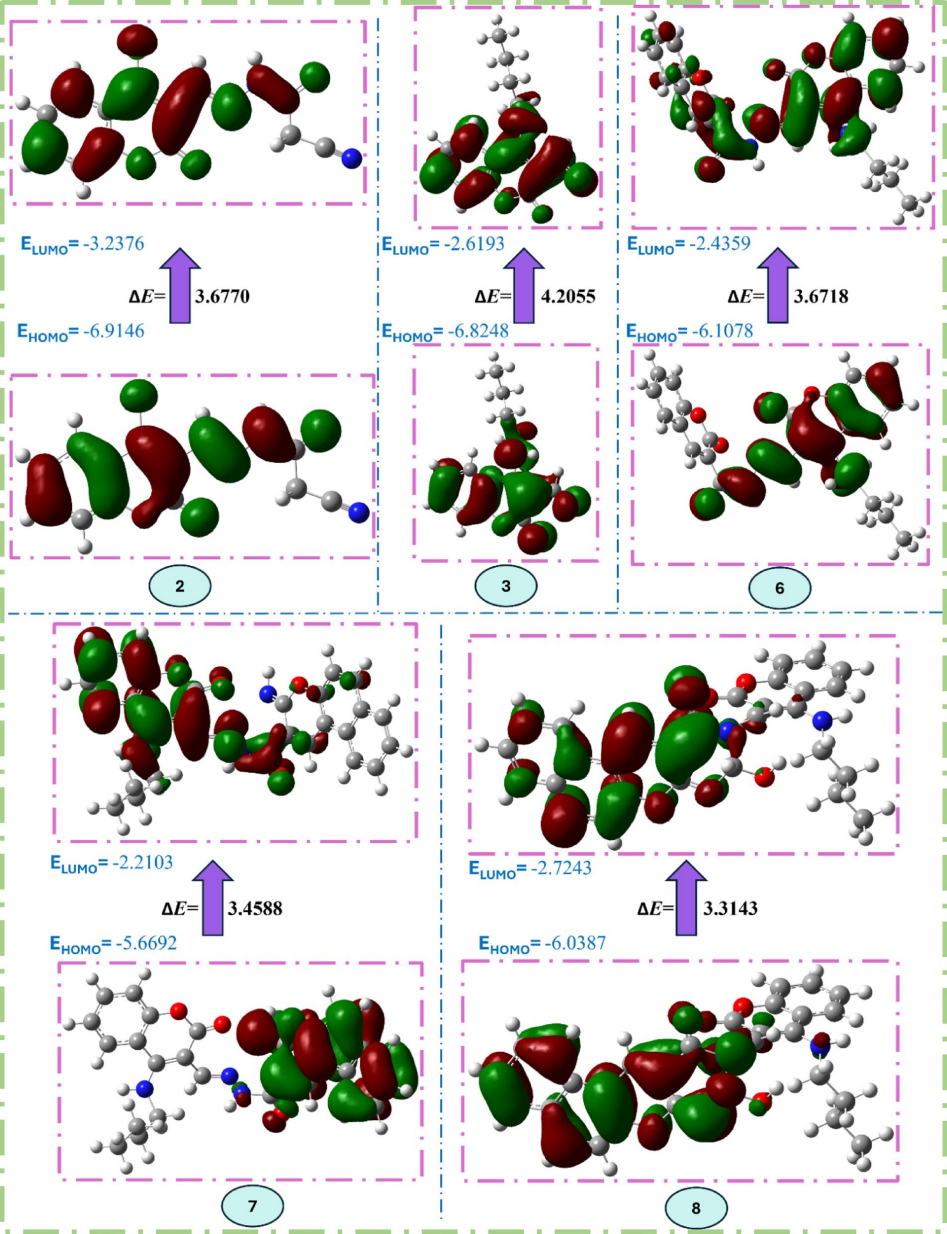
Representation of HOMO and LUMO coefficient distribution and the energy gap in eV of compounds 2, 3, 6, 7, and 8.

The energy gap (**Δ*E***), defined as the energy difference between HOMO and LUMO orbitals, is a critical parameter indicating molecular stability and reactivity and is presented in Fig. 10. Compound **8**, demonstrating the best antifungal activity (IC_50_ = 3.43 mg/mL) and the strongest docking affinities (-7.80 to -8.98 kcal/mol), exhibited the lowest energy gap (**Δ*E*** = 3.3143 eV) among the tested compounds, indicating enhanced molecular reactivity and electron transfer capability, characteristics beneficial for strong interactions with fungal cell targets, which aligns well with its observed high antifungal potency. Similarly, compound **7** also showed a relatively low energy gap (ΔE = 3.4588 eV), correlating well with its broader and stronger antifungal efficacy against *Fusarium oxysporum* and *Fusarium semitectum*, and its favorable docking affinities. The chemical softness (***S***) values revealed an interesting correlation with biological activity. Compound **8** had the highest softness (***S*** = 0.6034 eV^-1^), indicating significant electronic flexibility and an enhanced ability to form effective interactions with biological targets, consistent with its robust antifungal activity and favorable docking interactions. Compound **7**, similarly, showed relatively high chemical softness (***S*** = 0.5782 eV^-1^), in agreement with its observed multi-target inhibitory potential. Conversely, compound **3**, despite exhibiting good experimental antifungal activity (IC_50_ < 0.63 mg/mL), possessed the lowest softness (***S*** = 0.4755 eV^-1^) and highest energy gap (**Δ*E*** = 4.2055 eV), suggesting that additional factors such as solubility and membrane permeability could significantly influence its bioactivity beyond electronic interactions alone. Frontier orbital energies (HOMO and LUMO) further justified the experimental data. Compound **7** showed the highest HOMO energy (-5.6692 eV), reflecting greater electron-donating capacity, aligning with its strong docking affinities and robust antifungal activities. Compound **8**, with balanced HOMO (-6.0387 eV) and LUMO (-2.7243 eV) energies, displayed optimal electronic features that support its consistent antifungal potency against multiple pathogens. Additionally, the electrophilicity index (**ω**), reflecting electron-accepting capabilities, was highest for compound **2** (**ω** = 7.0075 eV), possibly explaining its notable antifungal performance (IC_50_ = 0.35 mg/mL) despite relatively moderate docking scores (-6.056 to -6.388 kcal/mol). Compound **6**, showing intermediate antifungal effectiveness (IC_50_ < 1.25 mg/mL against R. solani), possessed moderate quantum chemical properties (**Δ*E*** = 3.6718 eV, ***S*** = 0.5446 eV^-1^), consistent with its observed intermediate biological activity. Its moderate softness and energy gap likely reflect limited reactivity and interaction flexibility with fungal targets compared to compounds **7** and **8**. The dipole moment (***µ***) is another critical parameter influencing antifungal efficacy due to its role in molecular polarity and membrane permeation. Compound** 8** notably had the lowest dipole moment (8.3959 D), which might enhance membrane penetration and intracellular accessibility, partially explaining its broader antifungal activity compared to compounds with higher dipole moments, such as **7** (***µ*** = 15.0472 D) or **3** (***µ*** = 14.2160 D). Overall, the integrated analysis clearly shows a correlation between quantum chemical parameters derived from DFT calculations and observed antifungal activity. Specifically, lower energy gaps, increased softness, optimal electrophilicity, and suitable dipole moments may provide supportive insights into the observed trends in antifungal activity. These theoretical insights provide valuable guidance for the rational optimization of coumarin derivatives as antifungal agents. It should be emphasized that DFT-derived electronic descriptors are useful for supporting structure–activity trends; however, they should not be interpreted as direct predictors of biological activity.

## Conclusion

This study demonstrates the successful synthesis of structurally diverse coumarin–cyanoacetohydrazide derivatives through strategic C-4 amino substitution and heterocyclic annulation. The modular synthetic approach afforded access to a range of fused and functionalized coumarin frameworks with good to excellent yields. Screening of their biological activities revealed that several derivatives possess significant antifungal activity. Compounds **2**, **3**, **6**, **7**, and particularly **8** represent emerging potent antifungal candidates. Molecular docking analyses confirmed the high binding affinities of compounds **6**–**8** to multiple important fungal enzymes, in agreement with the obtained in vitro results, while DFT-derived quantum chemical descriptors provided mechanistic insight into their electronic behavior and reactivity. The strong correlation between theoretical parameters, docking scores, and experimentally detected antifungal activities reveals the predictive power of the combined in silico and in vitro approach. Overall, compound **8** represents a promising antifungal lead for further optimization, and the present findings offer a valuable platform for the design of next-generation coumarin-based antifungal agents with enhanced potency and spectrum of activity.

## Experimental

### Chemistry

The progress of the reactions was tracked using TLC (Kieselgel 60 F254, Merck), with spot detection under a 254 nm UV lamp. Melting points of the synthesized compounds were determined using a Mel-Temp II apparatus and are reported without correction. FT-IR spectra were obtained on a Nicolet iS10 FT-IR spectrometer at Ain Shams University’s Faculty of Science. ^1^H-NMR (300, 400, and/or 500 MHz) and ^13^C-NMR (100 and/or 75 MHz) spectra were recorded on a Varian Gemini spectrometer using DMSO-*d*_*6*_ as the solvent and TMS as the internal standard, conducted at both Ain Shams University’s Faculty of Science and the National Research Center. Elemental analysis was performed with a Perkin-Elmer 2400 CHN analyzer at the Microanalytical Center, Faculty of Science, Ain Shams University. Mass spectra were acquired using a Shimadzu GC–MS QP1000EX apparatus at the Faculty of Science, El-Azhar University.

4-Chloro-2-oxo-2*H*-chromene-3-carbaldehyde **1**^40^ and 2-cyanoacetohydrazide^41^, *N*’-((4-chloro -2-oxo-2*H*-chromen-3-yl)methylene)-2-cyanoacetohydrazide **2**^39^ and 2-(4-chlorobenzylidene) malononitrile^55^ were prepared according to literature method.

#### 4-(butylamino)-2-oxo-2*H*-chromene-3-carbaldehyde 3

A mixture of 4-chloro-2-oxo-2*H*-chromene-3-carbaldehyde **1** (0.01 mol, 2.08 g), *n*-butylamine (0.01 mol, 0.98 mL) was stirred for 12 h at ambient temperature in ethanol (20 mL). The formed precipitate was filtered off and then recrystallized from ethanol to obtain **3** as white crystals; m.p.: 120–122 °C, yield (47%). FT-IR (KBr, *ν*/cm^-1^): 3232 (NH), 3066 (C-H_arom_.), 2955, 2927, 2871 (C-H_aliph_.), 1694 (C = O), 1624 (C = C). ^1^H-NMR )DMSO-*d*_*6*_) δ (ppm): 11.79 (br. s, 1H, NH, exchangeable with D_2_O), 9.86 (s, 1H, -CHO), 8.21, 8.19 (dd, 1H, Ar–H, *J* = 8.3, *J* = 1.5 Hz), 7.73, 7.71, 7.96 (td, 1H, Ar–H, *J* = 7.8, *J* = 1.2 Hz), 7.37–7.31 (m, 2H, Ar–H), 3.89 (q, 2H, NCH_2_, *J* = 6.4 Hz), 1.70 (quint, 2H, NCH_2_CH_2_, *J* = 7.2 Hz), 1.46 (sext, 2H, NCH_2_CH_2_CH_2_, *J* = 7.2 Hz), 0.94 (t, 3H, CH_3_, *J* = 7.2 Hz). MS m/z (%): 245.01 (M^•+^; 34.71). Anal. Calcd. for C_14_H_15_NO_3_ (245.28): C, 68.56; H, 6.16; N, 5.71. Found: C, 68.45; H, 6.19; N, 5.78.

#### *N*’-((4-(butylamino)-2-oxo-2*H*-chromen-3-yl)methylene)-2-cyanoacetohydrazide 4

**Method (A)**

An equimolar mixture of *N*’-((4-chloro-2-oxo-2*H*-chromen-3-yl)methylene)-2-cyanoacetohydrazide **2** (0.01 mol, 2.89 g) and *n*-butylamine (0.01 mol, 0.98 mL) in dioxane (20 mL) was refluxed for 1 h. The formed precipitate on hot during the reflux was filtered off and then recrystallized from ethanol to give **4** with yield (98%).

**Method (B)**

An equimolar mixture of 4-(butylamino)-2-oxo-2*H*-chromene-3-carbaldehyde **3** (0.01 mol, 2.45 g) and 2-cyanoacetohydrazide (0.01 mol, 0.99 g) in ethanol (20 mL) was refluxed for 12 h. The formed precipitate on hot during the reflux was filtered off and then recrystallized from ethanol to give **4** with yield (87%).

**Method (C)**

An equimolar mixture of 3-(2-bromophenyl)-*N*’-((4-(butylamino)-2-oxo-2*H*-chromen-3-yl)methylene)-2-Cyanoacrylohydrazide **10** (0.005 mol, 2.465 g) and hydrazine (1 mL) was refluxed for 2 h in ethanol (20 mL). The formed precipitate during the reflux was filtered off and then recrystallized from ethanol to give **4** with yield (78%).

**Method (D)**

An equimolar mixture of 3-(2-bromophenyl)-*N*’-((4-(butylamino)-2-oxo-2*H*-chromen-3-yl)methylene)-2-Cyanoacrylohydrazide **10** (0.005 mol, 2.465 g) and malononitrile (0.005 mol, 0.33 g) was refluxed for 8 h in ethanol (20 mL). The formed precipitate during the reflux was filtered off and then recrystallized from ethanol to give **4** with yield (25%).

**4:** as yellow crystals, m.p.: 248–250 °C. FT-IR (KBr, *ν*/cm^-1^): 3241 (NH), 3089 (C-H_arom._), 2956, 2930, 2872 (C-H_aliph._), 2255 (C≡N), 1700 (C = O_(coumarin)_), 1665 (C = O_(amide)_), 1635 (C = N), 1594 (C = C). ^1^H-NMR )DMSO-*d*_6_) δ (ppm): 8.21 (d, 1H, Ar–H, *J* = 8.1 Hz), 7.63 (t, 1H, Ar–H, *J* = 7.15 Hz), 7.33 (br. d, 2H, Ar–H, *J* = 7.15 Hz), 3.88–3.87 (m, 2H, NCH_2_), 1.73–1.72 (m, 2H, NCH_2_CH_2_), 1.47–1.40 (m, 2H, NCH_2_CH_2_CH_2_), 0.90 (t, 3H, CH_3_, *J* = 7.15 Hz), for *anti*-isomer [11.72 (br. s, 1H, NH, exchangeable with D_2_O), 10.89 (br. s, 1H, NH, exchangeable with D_2_O), 8.51 (s, 1H, CH = N), 3.75 (s, 2H, CH_2_CN)], for *syn*-isomer [11.58 (br. s, 1H, NH, exchangeable with D_2_O), 9.49 (br. s, 1H, NH, exchangeable with D_2_O), 8.43 (s, 1H, CH = N), 4.12 (s, 2H, CH_2_CN)]. ^13^C-NMR )DMSO-*d*_6_) δ (ppm): 175.0, 161.1, 157.8, 154.4, 153.2, 147.2, 145.9, 133.0, 127.4, 123.7, 118.7, 117.5, 115.8, 90.1, 47.5, 47.2, 32.2, 31.9, 24.5, 19.6, 13.6. MS m/z (%): 326.12 (M^•+^; 13.26). Anal. Calcd. for C_17_H_18_N_4_O_3_ (326.36): C, 62.57; H, 5.56; N, 17.17. Found: C, 62.63; H, 5.59; N, 17.12.

#### *N’*-((4-(butylamino)-2-oxo-2*H*-chromen-3-yl)methylene)-2-imino-2*H*-chromene-3-carbohydrazide 5 and *N’*-((4-(butylamino)-2-oxo-2*H*-chromen-3-yl)methylene)-2-oxo-2*H*-chromene-3-carbohydrazide 6.

An equimolar mixture of *N’*-((4-(butylamino)-2-oxo-2*H*-chromen-3-yl)methylene)-2-cyanoacetohydrazide **4** (0.01 mol, 3.26 g) and salicylaldehyde (0.01 mol, 1.06 mL) in the presence of few drops of piperidine in ethanol (25 mL) was refluxed for 2 h. The formed precipitate was filtered off and then recrystallized from ethanol to give **6** with yield (25%), while the filtrate was allowed to evaporate, then the obtained solid was collected and recrystallized from ethanol to obtain to give **5** with yield (45%).

**5**: as orange crystals, m.p.: 270–272 °C. FT-IR (KBr, *ν*/cm^-1^): br. 3225, 3192 (NH), 3019 (C-H_arom_.), 2961, 2871 (C-H_aliph_.), 1692 (C=O_(coumarin)_), 1657 (C = O_(amide)_), 1623 (C=N), 1602 (C=C). ^1^H-NMR )DMSO-*d*_*6*_) δ (ppm): 10.99 (br. s, 1H, NH, exchangeable with D_2_O), 9.91 (br. s, 1H, NH, exchangeable with D_2_O), 8.75 (s, 1H, C_4_-H_(iminocoumarin)_), 8.16 (s, 1H, CH=N), 8.11 (br. s, 1H, NH, exchangeable with D_2_O), 7.70–6.80 (m, 8H, Ar–H), 3.89–3.75 (m, 2H, NCH_2_), 1.66–1.54 (m, 2H, NCH_2_CH_2_), 1.39–1.29 (m, 2H, NCH_2_CH_2_CH_2_), 0.87 (br. t, 3H, CH_3_). MS m/z (%): 430.78 (M^•+^; 10.21). Anal. Calcd. for C_24_H_22_N_4_O_4_ (430.46): C, 66.97; H, 5.15; N, 13.02. Found: C, 67.01; H, 5.11; N, 12.95.

**6:** as yellow crystals, m.p.: 228–230 °C. FT-IR (KBr, *ν*/cm^-1^): 3201 (NH), 3031 (C-H_arom_.), 2959, 2932, 2867 (C-H_aliph_.), 1714 (C=O_(coumarin)_), 1682 (C=O_(amide)_), 1626 (C=N), 1610 (C=C). ^1^H-NMR )DMSO-*d*_*6*_) δ (ppm): 11.70 (br. s, 1H, NH, exchangeable with D_2_O), 11.12 (br. s, 1H, NH, exchangeable with D_2_O), 8.84 (s, 1H, C_4_-H_(coumarin)_), 8.72 (s, 1H, CH=N), 8.25 (br. s, 1H, C_5_-H_(coumarin)_), 7.95 (br. s, 1H, C_5_-H_(coumarin)_), 7.73–7.35 (m, 6H, Ar–H), 3.94 (br. s, 2H, NCH_2_), 1.83–1.73 (m, 2H, NCH_2_CH_2_), 1.52–1.50 (m, 2H, NCH_2_CH_2_CH_2_), 0.94 (br. t, 3H, CH_3_). MS m/z (%): 431.13 (M^•+^; 42.35). Anal. Calcd. for C_24_H_21_N_3_O_5_ (431.45): C, 66.81; H, 4.91; N, 9.74. Found: C, 66.88; H, 4.94; N, 9.79.

#### ***N’***-((4-(butylamino)-2-oxo-2***H***-chromen-3-yl)methylene)-3-imino-3***H***-benzo[f]chromene-2-carbohydrazide 7.

An equimolar mixture of *N’*-((4-(butylamino)-2-oxo-2*H*-chromen-3-yl)methylene)-2-cyanoacetohydrazide **4** (0.01 mol, 3.26 g) and 2-hydroxynaphthaldehyde (0.01 mol, 1.72 g) in the presence of a few drops of piperidine in ethanol (30 mL) was refluxed for 1 h. The formed precipitate on hot during the reflux was filtered off and then recrystallized from ethanol to give **7** as deep yellow crystals; m.p.: 222–224 °C, yield (59%). FT-IR (KBr, *ν*/cm^-1^): 3487, 3320 (NH), 3061 (C-H_arom_.), 2953, 2867 (C-H_aliph_.), 1688 (C=O_(coumarin)_), 1664 (C=O_(amide)_), 1633 (C=N). ^1^H-NMR )DMSO-*d*_*6*_) δ (ppm): 13.58 (br. s, 1H, NH, exchangeable with D_2_O), 12.63 (br. s, 1H, NH, exchangeable with D_2_O), 11.01 (br. s, 1H, NH, exchangeable with D_2_O), 9.11 (s, 1H, C_1_-H_(3-iminobenzochromene)_, 8.47 (d, 1H, Ar–H), 8.42 (s, 1H, CH=N), 8.20 (d, 1H, Ar–H), 8.08 (d, 1H, Ar–H), 7.93 (t, 1H, Ar–H), 7.69 (d, 1H, Ar–H), 7.58–7.29 (m, 5H, Ar–H), 3.96–3.85 (m, 2H, NCH_2_), 1.92–1.80 (br. s, 2H, NCH_2_CH_2_), 1.58–1.52 (br. s, 2H, NCH_2_CH_2_CH_2_), 0.98 (t, 3H, CH_3_). MS m/z (%): 480.31 (M^•+^; 16.27). Anal. Calcd. for C_28_H_24_N_4_O_4_ (480.52): C, 69.99; H, 5.03; N, 11.66. Found: C, 70.07; H, 5.00; N, 11.60.

#### 10-(((4-(butylamino)-2-oxo-2*H*-chromen-3-yl)methylene)amino)-9-hydroxy-9,10-dihydro-11*H*-benzo[5,6]chromeno[2,3-*d*]pyrimidin-11-one 8

A mixture of *N’*-((4-(butylamino)-2-oxo-2*H*-chromen-3-yl)methylene)-3-imino-3*H*-benzo[*f*]chromene-2-carbohydrazide **7** (0.01 mol, 4.80 g), triethyl orthoformate (TEOF) (5 mL) and acetic anhydride (5 mL) was heated under reflux for 12 h. The reflux-induced precipitate was filtered off, followed by recrystallized from ethanol to afford **8** as deep yellow amorphous; m.p.: 276–278 °C, yield (78%). FT-IR (KBr, *ν*/cm^-1^): 3435 (OH), 3260 (NH), 3055 (C-H_arom_.), 2957, 2930, 2870 (C-H_aliph_.), 1677 (br.) (C=O), 1629 (C=N), 1614 (C=C). ^1^H-NMR )DMSO-*d*_6_) δ (ppm): 10.83 (br. s, 1H, NH, exchangeable with D_2_O), 9.07 (s, 1H, C_4_-H_(coumarin)_), 8.66 (s, 1H, CH=N), 8.23–7.37 (m, 10H, Ar–H), 5.74 (s, 1H, C_2_-H_(pyrimidinone)_), 5.13 (br. s, 1H, OH, exchangeable with D_2_O), 3.94 (m, 2H, NCH_2_), 1.76 (m, 2H, NCH_2_CH_2_), 1.45 (m, 2H, NCH_2_CH_2_CH_2_), 0.93 (t, 3H, CH_3_). MS m/z (%): 508.37 (M^•+^; 21.45). Anal. Calcd. for C_29_H_24_N_4_O_5_ (508.53): C, 68.49; H, 4.76; N, 11.02. Found: C, 68.44; H, 4.73; N, 11.08.

#### *N*-(2-oxo-3-((2-(2-(4-oxothiazolidin-2-ylidene)acetyl)hydrazineylidene)methyl)-2*H*-chromen-4-yl)acetamide 9

An equimolar mixture of *N’*-((4-(butylamino)-2-oxo-2*H*-chromen-3-yl)methylene)-2-cyanoacetohydrazide **4** (0.01 mol, 3.26 g) and thioglycolic acid (0.01 mol, 0.69 mL) in acetic acid (20 mL) was refluxed for 12 h. The formed precipitate on hot during the reflux was filtered off and then recrystallized from ethanol to give **9** as brown amorphous; m.p.: 354–356 °C, yield (36%). FT-IR (KBr, *ν*/cm^-1^): 3231 (br.) (NH), 3061 (C-H_arom_.), 2927 (C-H_aliph_.), 1740 (C=O_(1,3-thiazolidin-4-one)_), 1688 (C=O_(amide)_) 1623 (C=N), 1608 (C=C). ^1^H-NMR )DMSO-*d*_*6*_) δ (ppm): 12.04 (br. s, 1H, NH, exchangeable with D_2_O), 9.64 (br. s, 1H, NH, exchangeable with D_2_O), 9.62 (br. s, 1H, NH, exchangeable with D_2_O), 8.93 ( s, 1H, CH = N), 8.12 (d, 1H, Ar–H, *J* = 7.9 Hz), 7.74–7.42 (m, 3H, Ar–H), 5.81 (s, 1H, CH_(olefinic)_), 3.94 (s, 2H, SCH_2_), 2.05 (s, 3H, CH_3_). MS m/z (%): 386.82 (M^•+^; 12.08). Anal. Calcd. for C_17_H_14_N_4_O_5_S (386.38): C, 52.85; H, 3.65; N, 14.50. Found: C, 52.89; H, 3.69; N, 14.45.

#### 3-(2-bromophenyl)-*N’*-((4-(butylamino)-2-oxo-2*H*-chromen-3-yl)methylene)-2-

**Cyanoacrylohydrazide 10**.

An equimolar mixture of *N’*-((4-(butylamino)-2-oxo-2*H*-chromen-3-yl)methylene)-2-cyanoacetohydrazide **4** (0.01 mol, 3.26 g) and 2-bromobenzaldehyde (0.01 mol, 1.16 mL) in ethanol (20 mL) in the presence of few drops of triethylamine was refluxed for 2 h. the precipitate that developed on hot during the reflux was filtered off and subjected to recrystallization from ethanol to give **10** as orange crystals; m.p.: 230–232 °C, yield (30%). FT-IR (KBr, *ν*/cm^-1^): 3276 (NH), 3044 (C-H_arom._), 2958, 2927, 2858 (C-H_aliph._), 2217 (C≡N), 1712 (C=O_(coumarin)_), 1684 (C=O_(amide)_), 1631 (C=N), 1598 (C=C). ^1^H-NMR )DMSO-*d*_6_) δ (ppm): 12.07 (br. s, 1H, NH, exchangeable with D_2_O), 11.07 (br. s, 1H, NH, exchangeable with D_2_O), 8.91 (s, 1H, CH = N), 8.40 (s, 1H, CH_(olefinic)_), 8.25 (d, 1H, Ar–H, *J* = 8.9 Hz), 7.99 (d, 1H, Ar–H, *J* = 7.6 Hz), 7.84 (d, 1H, Ar–H, *J* = 7.9 Hz), 7.69–7.48 (m, 3H, Ar–H), 7.38–7.34 (m, 2H, Ar–H), 3.93(q, 2H, NCH_2_, *J* = 6.6 Hz), 1.83 (quint, 2H, NCH_2_CH_2_, *J* = 7.2 Hz), 1.51 (sextet, 2H, NCH_2_CH_2_CH_2_, *J* = 7.2 Hz), 0.95 (t, 3H, CH_3_, *J* = 7.2 Hz). ^13^C-NMR )DMSO-*d*_6_) δ (ppm): 168.3, 161.1, 158.02, 155.0, 150.0, 149.5, 137.6, 133.4, 133.3, 132.3, 130.0, 128.3, 127.4, 124.1, 124.5, 123.7, 117.5, 115.0, 114.4, 93.5, 47.6, 31.9, 19.6, 13.6. MS m/z (%): 495.41 (M^•+^  + 2; 30.42), 493.93 (M^•+^; 29.20). Anal. Calcd. for C_24_H_21_BrN_4_O_3_ (493.36): C, 58.43; H, 4.29; Br, 16.20; N, 11.36; Found: C, 58.48; H, 4.34; Br, 16.26; N, 11.30.

#### ethyl-3-(4-(butylamino)-2-oxo-2***H***-chromen-3-yl)-2-cyanoacrylate 11.

**Method (A):**

An equimolar mixture of 3-(2-bromophenyl)-*N*’-((4-(butylamino)-2-oxo-2*H*-chromen-3-yl)methylene)-2-Cyanoacrylohydrazide **10** (0.005 mol, 2.465 g) and ethyl cyanoacetate (0.005 mol, 0.541 mL) was refluxed for 8 h in ethanol (20 mL) in presence of few drops of piperidine. The formed precipitate during the reflux was filtered off and then recrystallized from ethanol to give **11** with yield (58%).

**Method (B):**

An equimolar mixture of *N’*-((4-(butylamino)-2-oxo-2*H*-chromen-3-yl)methylene)-2-cyanoacetohydrazide **4** (0.01 mol, 3.26 g) and ethyl cyanoacetate (0.01 mol, 1.063 mL) was refluxed for 12 h in ethanol (20 mL) in presence of few drops of piperidine. The formed precipitate on hot during the reflux was filtered off and then recrystallized from ethanol to give **11** with yield (41%).

**11**: As yellow crystals, m.p.: 80–82 °C. FT-IR (KBr, *ν*/cm^-1^): 3339 (NH), 3075 (C-H_arom_.), 2955, 2928 (C-H_(aliph_._)_), 2213 (C≡N), 1729 (C=O_(ester)_), 1690 (C=O_(coumarin)_), 1608 (C=C). ^1^H-NMR )DMSO-*d*_*6*_) δ (ppm): 8.46 (br. s, 1H, NH, exchangeable with D_2_O), 8.30 (s, 1H, CH_(olefinic)_), 8.05 (d, 1H, Ar–H, *J* = 8.0 Hz), 7.53 (t, 1H, Ar–H, *J* = 8.0 Hz,* J* = 7.6 Hz ), 7.27 (t, 1H, Ar–H, *J* = 7.6 Hz ), 7.19 (d, 1H, Ar–H, *J* = 8.0 Hz), 4.22 (q, 2H, OCH_2,_ *J* = 7.2 Hz, *J* = 7.6 Hz), 3.49–3.45 (m, 2H, NCH_2_), 1.61–1.52 (m, 2H, NCH_2_CH_2_), 1.40–1.29 (m, 5H, NCH_2_CH_2_CH_2_ + CH_3_), 0.94 (t, 3H, CH_3_, *J* = 7.2 Hz, *J* = 7.6 Hz). ^13^C-NMR )DMSO-*d*_6_) δ (ppm): 165.7, 159.8, 159.0, 154.4, 153.5, 141.4, 133.4, 124.8, 124.7, 118.4, 117.1, 107.3, 105.3, 61.7, 40.8, 30.9, 20.2, 14.4, 14.1. MS m/z (%): 340.38 (M^•+^; 11.06). Anal. Calcd. for C_19_H_20_N_2_O_4_ (340.38): C, 67.05; H, 5.92; N, 8.23. Found: C, 66.99; H, 5.95; N, 8.28.

#### 3-(3-methylthiophen-2-yl)-2-(4-oxo-1,4-dihydrochromeno[4,3-***c***]pyrazole-1-carbonyl)acrylamide 12.

An equimolar mixture of *N’*-((4-(butylamino)-2-oxo-2*H*-chromen-3-yl)methylene)-2-cyanoaceto hydrazide **4** (0.01 mol, 3.26 g) and 3-methylthiophene-2-carbaldehyde (0.01 mol, 1.07 mL) in ethanol (20 mL) was refluxed for 12 h in the presence of a few drops of piperidine. the precipitate that developed on hot during the reflux was filtered off and recrystallized from ethanol to give **12** as green crystals; m.p.: 310–312 °C, yield (48%). FT-IR (KBr, *ν*/cm^-1^): 3402 (br.) (NH_2_), 3059 (C-H_arom._), 2942, 2866 (C-H_aliph._), 1735 (C=O_(coumarin)_), 1706 (C=O_(amide)_), 1605 (C=N or C=C). ^1^H-NMR )DMSO-*d*_6_) δ (ppm): 9.93 (d, 1H, Ar–H, *J* = 8.4 Hz), 8.90 (s, 1H, C_3_-H_(pyrazole)_), 7.82 (t, 1H, Ar–H, *J* = 8.4 Hz, *J* = 7.8 Hz), 7.58 (t, 1H, Ar–H, *J* = 8.1 Hz, *J* = 7.8 Hz), 7.53 (d, 1H, Ar–H, *J* = 8.4 Hz), 7.25, 6.91 (br. s, 2H, NH_2_, exchangeable with D_2_O), 7.08 (d, 1H, Ar–H_(thiophene)_, *J* = 5.1 Hz), 6.69 (d, 1H, Ar–H_(thiophene)_, *J* = 5.1 Hz), 6.60 (s, 1H, CH_(olefinic)_), 2.09 (s, 3H, CH_3_). MS m/z (%): 379.82 (M^•+^; 33.25). Anal. Calcd. for C_19_H_13_N_3_O_4_S (379.39): C, 60.15; H, 3.45; N, 11.08; S, 8.45. Found: C, 60.09; H, 3.42; N, 11.01; S, 8.51.

#### *N’*-((4-(butylamino)-2-oxo-2*H*-chromen-3-yl)methylene)-2-cyano-3,3-dimercaptoacrylo hydrazide 13.

A mixture of *N’*-((4-(butylamino)-2-oxo-2*H*-chromen-3-yl)methylene)-2-cyanoacetohydrazide **4** (0.005 mol, 1.63 g) and carbon disulfide (5 mL) in ethanol (25 mL) was refluxed for 12 h in the presence of few drops of triethylamine. the precipitate that developed on hot during the reflux precipitate was filtered off, followed by recrystallization in ethanol to award **13** as brown amorphous; m.p.: 190–192 °C, yield (65%). FT-IR (KBr, *ν*/cm^-1^): 3433 (br.) (NH), 2957, 2927, 2870 (C-H_aliph._), 2211 (C≡N), 1677 (br.) (C=O), 1625 (C=N). ^1^H-NMR )DMSO-*d*_6_) δ (ppm): 13.06 (br. s, 1H, NH, exchangeable with D_2_O), 10.96 (br. s, 1H, NH, exchangeable with D_2_O), 10.19 (br. s, 2H, SH, exchangeable with D_2_O), 8.55 (s, 1H, CH=N), 8.23 (d, 1H, Ar–H, *J* = 8.1 Hz), 7.65 (t, 1H, Ar–H, *J* = 7.2 Hz, *J* = 7.8 Hz), 7.36–7.34 (m, 2H, Ar–H), 3.94–3.92 (m, 2H, NCH_2_), 1.81 (quint, 2H, NCH_2_CH_2_, *J* = 7.2 Hz), 1.50 (sextet, 2H, NCH_2_CH_2_CH_2_, *J* = 7.2 Hz, *J* = 7.5 Hz), 0.93 (t, 3H, CH_3_, *J* = 7.2 Hz). MS m/z (%): 402.59 (M^•+^; 29.33). Anal. Calcd. for C_18_H_18_N_4_O_3_S_2_ (402.49): C, 53.72; H, 4.51; N, 13.92. Found: C, 53.68; H, 4.54; N, 13.97.

#### 1-(((4-(butylamino)-2-oxo-2*H*-chromen-3-yl)methylene)amino)-4,6-dimethyl-2-oxo-1,2-dihydropyridine-3-carbonitrile 14.

An equimolar mixture of *N’*-((4-(butylamino)-2-oxo-2*H*-chromen-3-yl)methylene)-2-cyanoacetohydrazide **4** (0.01 mol, 3.26 g) and acetylacetone (0.01 mol, 1 mL) was refluxed for 24 h in ethanol (25 mL) in the presence of few drops of piperidine. The formed precipitate on hot during the reflux was filtered off and then recrystallized from ethanol to give **14** as yellow crystals; m.p.: 282–284 °C, yield (71%). FT-IR (KBr, *ν*/cm^-1^): 3267 (NH), 3033 (C-H_arom_.), 2962, 2932, 2874 (C-H_aliph_.), 2213 (C≡N), 1708 (C=O_(coumarin)_), 1651 (C=O_(pyridone)_), 1634 (C=N). ^1^H-NMR )DMSO-*d*_*6*_) δ (ppm): 10.65 (br. s, 1H, NH, exchangeable with D_2_O), 8.84 (s, 1H, CH=N), 8.30 (br. s, 1H, Ar–H), 7.75 (br. s, 1H, Ar–H), 7.47–7.35 (m, 2H, Ar–H), 6.39 (s, 1H, C_5_-H_(pyridone)_), 4.11–3.88 (m, 2H, NCH_2_), 2.34 (s, 6H, 2CH_3_), 1.85–1.64 (m, 2H, NCH_2_CH_2_), 1.52–1.31 (m, 2H, NCH_2_CH_2_CH_2_), 0.90 (t, 3H, CH_3_). MS m/z (%): 390.95 (M^•+^; 17.76). Anal. Calcd. for C_22_H_22_N_4_O_3_ (390.44): C, 67.68; H, 5.68; N, 14.35. Found: C, 67.76; H, 5.65; N, 14.30.

#### 1-(((4-(butylamino)-2-oxo-2***H***-chromen-3-yl)methylene)amino)-5-chloro-2-hydroxy-4-methyl-6-oxo-1,6-dihydropyridine-3-carboxamide 15.

An equimolar mixture of *N’*-((4-(butylamino)-2-oxo-2*H*-chromen-3-yl)methylene)-2-cyanoacetohydrazide **4** (0.01 mol, 3.26 g) and ethyl-2-chloroacetoacetate (0.01mol, 1.38 g) in ethanol (25 mL) was refluxed for 8 h in the presence of a few drops of piperidine. The formed precipitate was filtered off on hot and recrystallized from ethanol to give **15 as** yellowish green crystal; mp. 152–154°C, Yield (74%). FT-IR (KBr, *ν*/cm^-1^): 3393 (OH), 3253 (br.) (NH, NH_2_), 3029 (C-H_arom._), 2961, 2930, 2870 (C-H_aliph._), 1679, 1646 (C=O), 1616 (C=N or C=C). ^1^H-NMR )DMSO-*d*_6_) δ (ppm): 12.13 (br. s, 1H, OH, exchangeable with D_2_O), 11.09 (br. s, 1H, NH, exchangeable with D_2_O), 8.43 (s, 1H, CH=N), 8.25 (d, 1H, Ar–H, *J* = 8.1 Hz), 7.66 (t, 1H, Ar–H, *J* = 7.2 Hz), 7.39–7.34 (m, 2H, Ar–H), 7.24 (br. s, 2H, NH_2_, exchangeable with D_2_O), 3.94–3.92 (q, 2H, NCH_2_), 2.46 (s, 3H, CH_3_), 1.87–1.75 (m, 2H, NCH_2_CH_2_), 1.60–1.43 (m, 2H, NCH_2_CH_2_CH_2_), 0.93 (t, 3H, CH_3_, *J* = 7.5 Hz). MS m/z (%): 446.79 (M^•+^  + 2; 11.43), 444.61 (M^•+^; 8.72). Anal. Calcd. for C_21_H_21_ClN_4_O_5_ (444.87): C, 56.70; H, 4.76; Cl, 7.99; N, 12.59. Found: C, 56.74; H, 4.72; Cl, 8.04; N, 12.62.

#### 6-amino-1-(((4-(butylamino)-2-oxo-2*H*-chromen-3-yl)methylene)amino)-4-(4-chloro

Phenyl)-2-oxo-1,2-dihydropyridine-3,5-dicarbonitrile 16.

An equimolar mixture of *N’*-((4-(butylamino)-2-oxo-2*H*-chromen-3-yl)methylene)-2-cyanoacetohydrazide **4** (0.01 mol, 3.26 g) and 2-(4-chlorobenzylidene)malononitrile^55^ (0.01 mol, 1.88 g) in (20 mL) ethanol in the presence of a few drops of piperidine was refluxed for 24h. the precipitate that developed on hot during the reflux precipitate was filtered off, followed by recrystallization in ethanol to give **16** as brown amorphous; m.p.: 180–182 °C, yield (32%). FT-IR (KBr, *ν*/cm^-1^): 3317 (br.) (NH, NH_2_), 3071 (C-H_arom._), 2933, 2869 (C-H_aliph._), 2212 (C≡N), 1738 (C=O), 1671 (C = O_(pyridone)_), 1607 (C = N or C=C). ^1^H-NMR )DMSO-*d*_6_) δ (ppm): 9.44 (br. s, 1H, NH, exchangeable with D_2_O), 8.67 (s, 1H, CH = N), 7.66–7.36 (m, 8H, Ar–H), 5.37 (br. s, 2H, NH_2_, exchangeable with D_2_O), 3.99 (q, 2H, NCH_2_), 1.68–1.59 (m, 2H, NCH_2_CH_2_), 1.46–1.38 (m, 2H, NCH_2_CH_2_CH_2_), 0.97 (t, 3H, CH_3_, *J* = 7.5 Hz). MS m/z (%): 514.02 (M^•+^  + 2; 16.00), 512.71 (M^•+^; 20.08). Anal. Calcd. for C_27_H_21_ClN_6_O_3_ (512.95): C, 63.22; H, 4.13; Cl, 6.91; N, 16.38. Found: C, 63.29; H, 4.10; Cl, 6.96; N, 16.34.

#### 3-(4-(butylamino)-2-oxo-2***H***-chromen-3-yl)-2-cyanoacrylamide 18.

An equimolar mixture of *N’*-((4-(butylamino)-2-oxo-2*H*-chromen-3-yl)methylene)-2-cyanoacetohydrazide **4** (0.01 mol, 3.26 g) and cyanoacetamide (0.01 mol, 0.84 g) was refluxed for 12 h in ethanol (20 mL) in the presence of few drops of piperidine. The formed precipitate during the reflux was filtered off and then recrystallized from ethanol to give **18** as deep yellow amorphous; m.p.: 130–132 °C, yield (33%). FT-IR (KBr, *ν*/cm^-1^): 3361, 3201 (NH, NH_2_), 3073 (C-H_arom_.), 2956, 2931 (C-H_(aliph_._)_), 2207 (C≡N), 1700 (C = O_(coumarin)_), 1670 (C=O_(amide)_), 1611 (C=C). ^1^H-NMR )DMSO-*d*_*6*_) δ (ppm): 9.53 (br. s, 2H, NH_2_, exchangeable with D_2_O), 8.96 (s, 1H, NH, exchangeable with D_2_O), 8.65 (s, 1H, CH_(olefinic)_), 8.33 (d, 1H, Ar–H), 7.63–7.61 (m, 1H, Ar–H), 7.46–7.35 (m, 2H, Ar–H), 3.67–3.60 (m, 2H, NCH_2_), 1.66–1.54 (m, 2H, NCH_2_CH_2_), 1.47–1.33 (m, 2H, NCH_2_CH_2_CH_2_), 0.95 (t, 3H, CH_3_). MS m/z (%): 311.02 (M^•+^; 17.42). Anal. Calcd. for C_17_H_17_N_3_O_3_ (311.34): C, 65.58; H, 5.50; N, 13.50. Found: C, 65.51; H, 5.52; N, 13.59.

### Antifungal activity

**I- Screening:**

Antifungal activity of compounds was evaluated qualitatively by testing their inhibition potential against five fungal plant pathogens (*Fusarium solani*, *Fusarium oxysporum*, *Fusarium semitectum*, *Alternaria solani* and *Rhizoctonia solani*) in a concentration of 34 mg/mL. Tested compounds were dissolve in DMSO and the antifungal test was carried out by agar well diffusion method described by Riss et al*.*^56^. Warm Czapek-Dox’s agar medium seeded with spore suspension (15 × 10^4^ spore/mL) of the tested fungi was poured in sterilized petri dishes. After solidification, wells (8 mm in diameter) were bunched on the agar medium using sterile cork borer and 100 µl of the tested concentration was inoculated in these wells. Wells inoculated with 100 µl DMSO were used as control. The plates were incubated at 25 °C for 5 days. The inhibitory zone diameter was observed and measured (mm) to determine the antifungal activity.

**II- Estimation of IC**_**50**_ **:**

Depending on the obtained screening results, compounds that showed a detectable antifungal activity (Compounds **2**,** 3**,** 6**,** 7,** and **8**) have been selected to estimate their IC_50_ against the most inhibited fungal pathogen using the percent inhibition of mycelial growth method (PIMG)^57,58^. The tested compounds (dissolved in DMSO) were added to warm Czapek-Dox’s agar medium to obtain them in a final concentration range (0.63–5 mg/mL for compounds **2**,** 3**; 0.63–7.5 mg/mL for compound** 8**; 0.31–2.5 mg/mL for compound **7**; 1.25–5 mg/mL for compound** 6**). Then the medium was poured in sterilized petri dishes and left to solidify. Fluconazole was tested as the antifungal control at 0.1–0.8 mg/mL concentration range. After solidification, plugs of the tested fungi (0.8 cm) were inoculated at the center of the plates, and the plates were incubated at 25 °C for 6 days. The test was carried out in a triplicate manner. The result was taken by measuring the growth diameter for the plates inoculated with the compounds and the control plates, and calculating PIMG as follows:

PIMG = G_1_ – G_2_ / G_1_ × 100.

- G_1_: Diameter of fungal growth in the control plates.
- G_2_: Diameter of fungal growth in plates inoculated with the tested compounds.

IC_50_ (concentration yields 50% growth inhibition was calculated by plotting dose response curve for the mean values of the obtained results.

### Computational procedures

Gaussian(R) 09 D.01^59^ (Semichem Inc., Shawnee Mission, KS, USA) was used to perform density functional theory (DFT) calculations at the B3LYP level^60,61^ in conjunction with the 6-31G(d,p) basis set, and AutoDock Vina^62^ Was used to perform the molecular docking study and theoretical calculations and results of the analyzed compounds. The PyMOL software was used to view the docking results, and Gauss View 6.0.16 (Semichem Inc., Shawnee Mission, KS, USA) was used to visualize the DFT data.

## Supplementary Information

Supplementary Information.

## Data Availability

The data supporting this article have been included as part of the Supplementary Information. Data for compound 2 “N’-((4-Chloro-2-oxo-2H-chromen-3-yl)methylene)-2-cyanoacetohydrazide”, including physical and spectral data (IR, 1H and 13C NMR and Mass spectra) are available at our article “Synthesis, Antioxidant and Antiproliferative Evaluation, Molecular Docking and DFT Studies of Some Novel Coumarin and Fused Coumarin Derivatives” at 10.1002/cbdv.202300706.
