# Supplementary material for: Synthesis of some novel coumarin-based heterocycles, elucidation of their antifungal behavior, molecular docking and computational studies
Source: Sci Rep. 2026 Apr 13;16:12185. doi: 10.1038/s41598-026-43854-5 (PMC13076868; doi:10.1038/s41598-026-43854-5)
Supplement: Supplementary file 1 — Supplementary Information. [file 41598_2026_43854_MOESM1_ESM.pdf]

Synthesis of Some Novel Coumarin-based Heterocycles, Elucidation of their antifungal behavior, Molecular Docking and Computational Studies

Mahmoud F. Ismail[a], Mounir A. I. Salem[a], Magda I. Marzouk[a], Naglaa F. H. Mahmoud[a], Nashwa, H. Abdullah[b] and Mustafa A. S. Gouda[a,*]

**E-mail: mustafagouda@sci.asu.edu.eg*

[a] Department of Chemistry, Faculty of Science, Ain Shams University, 11566, Abbassia, Cairo, Egypt

[b] Botany and Microbiology Department, Faculty of Science, Capital University, 11795, Ain Helwan, Cairo, Egypt

Fax: +(202) 24831836; phone: +(202) 24831836.

***Corresponding author; e-mail: mustafagouda@sci.asu.edu.eg**

Full spectroscopic data.

Spectroscopic data

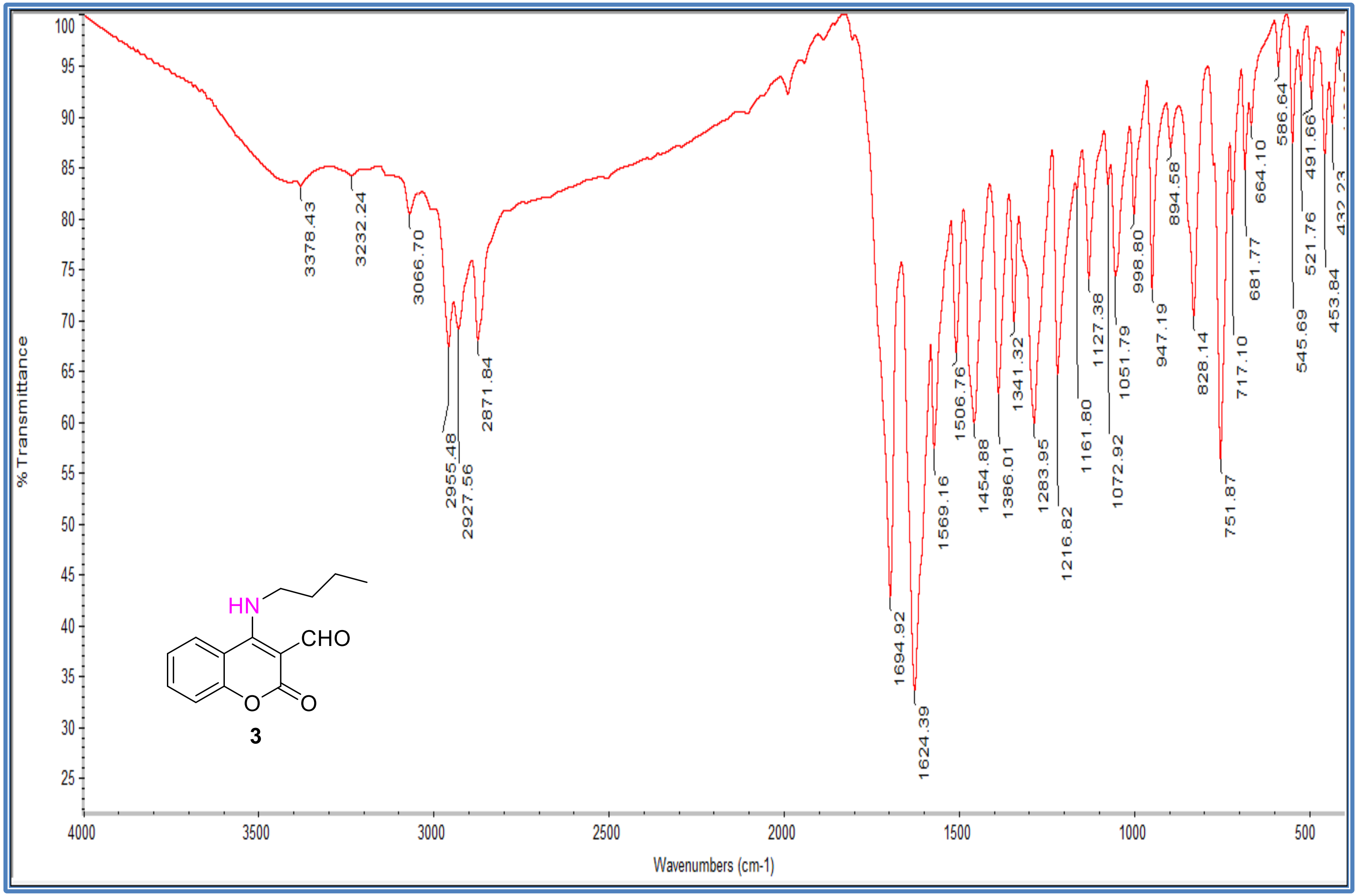

Spectroscopic data

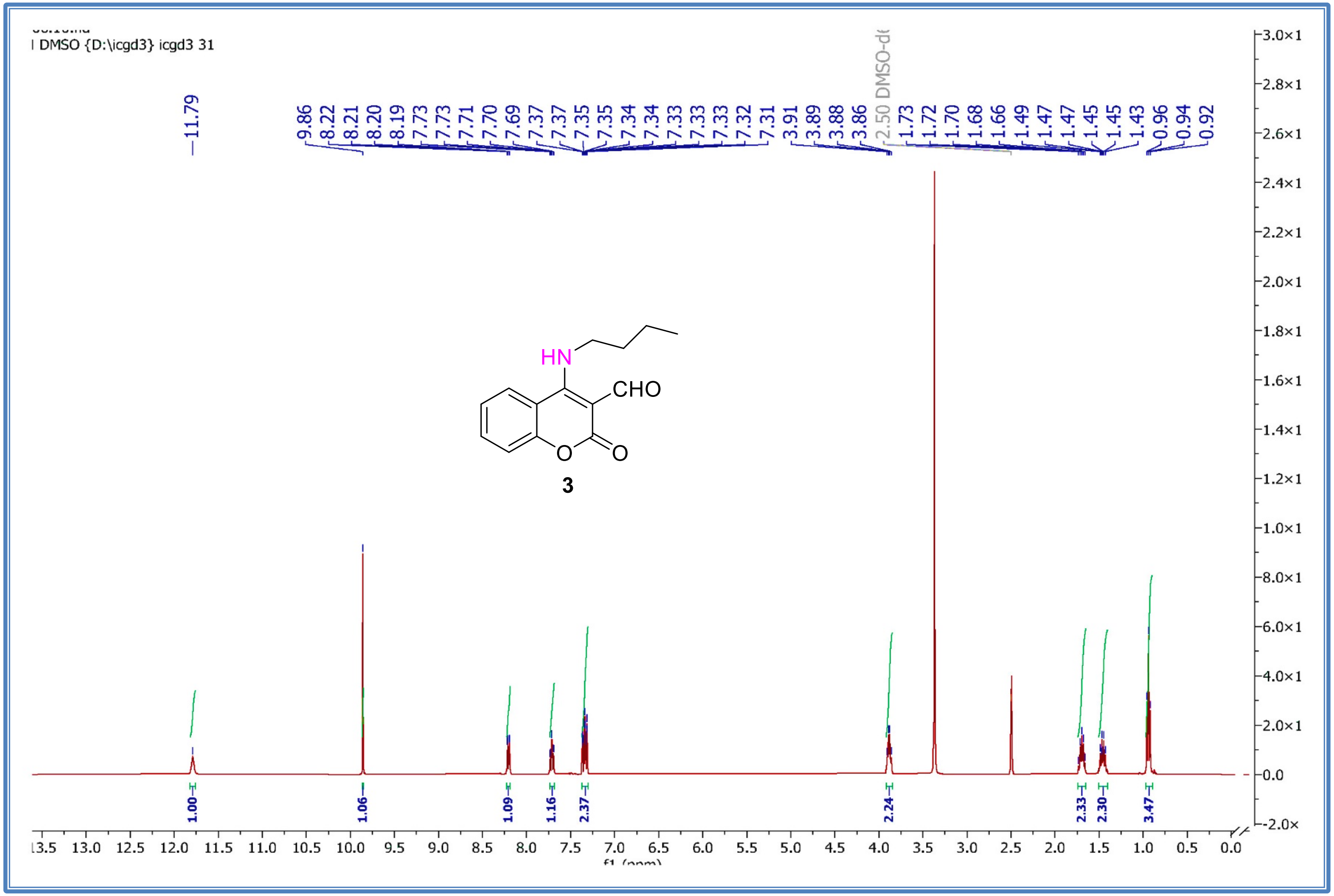

Spectroscopic data

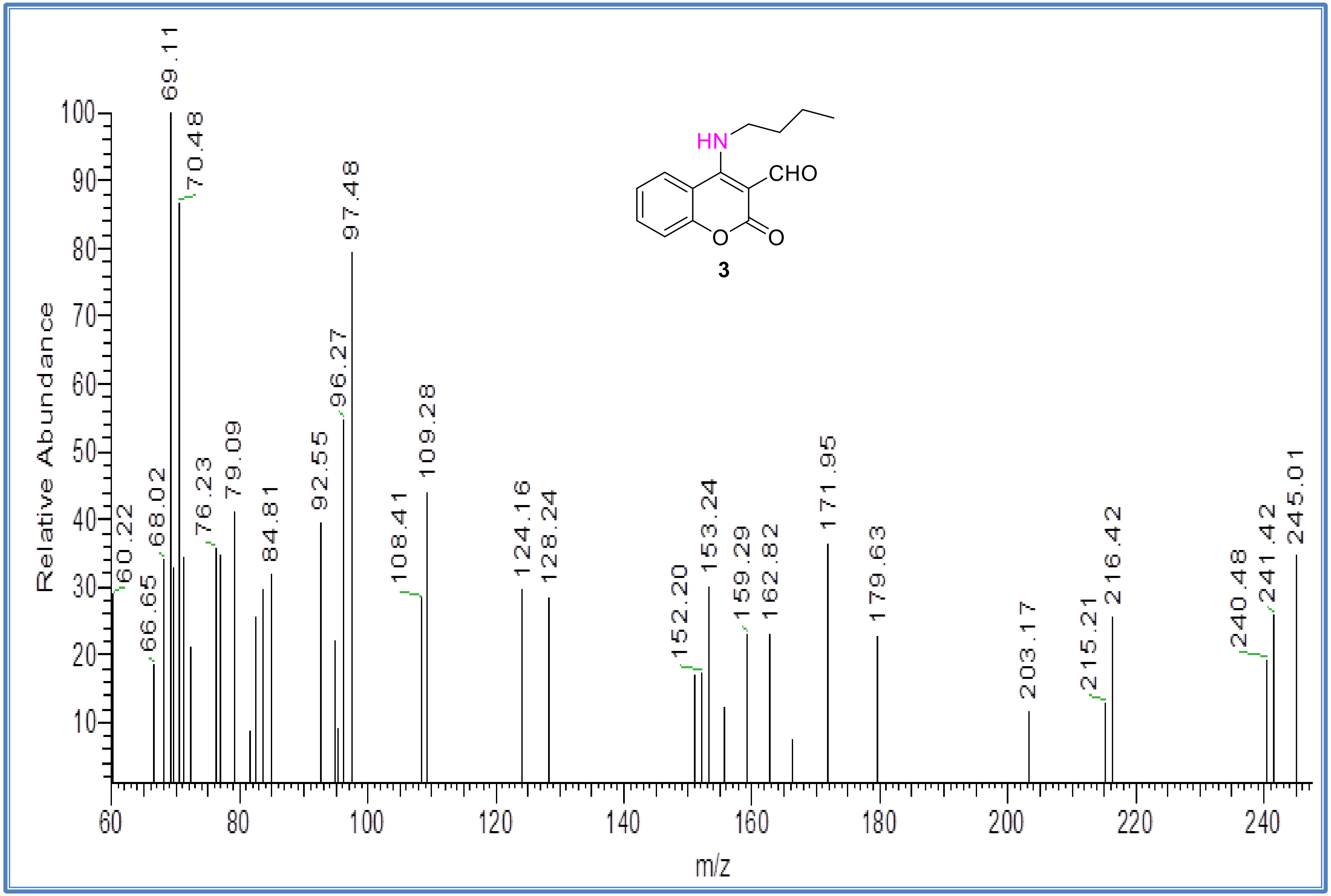

Spectroscopic data

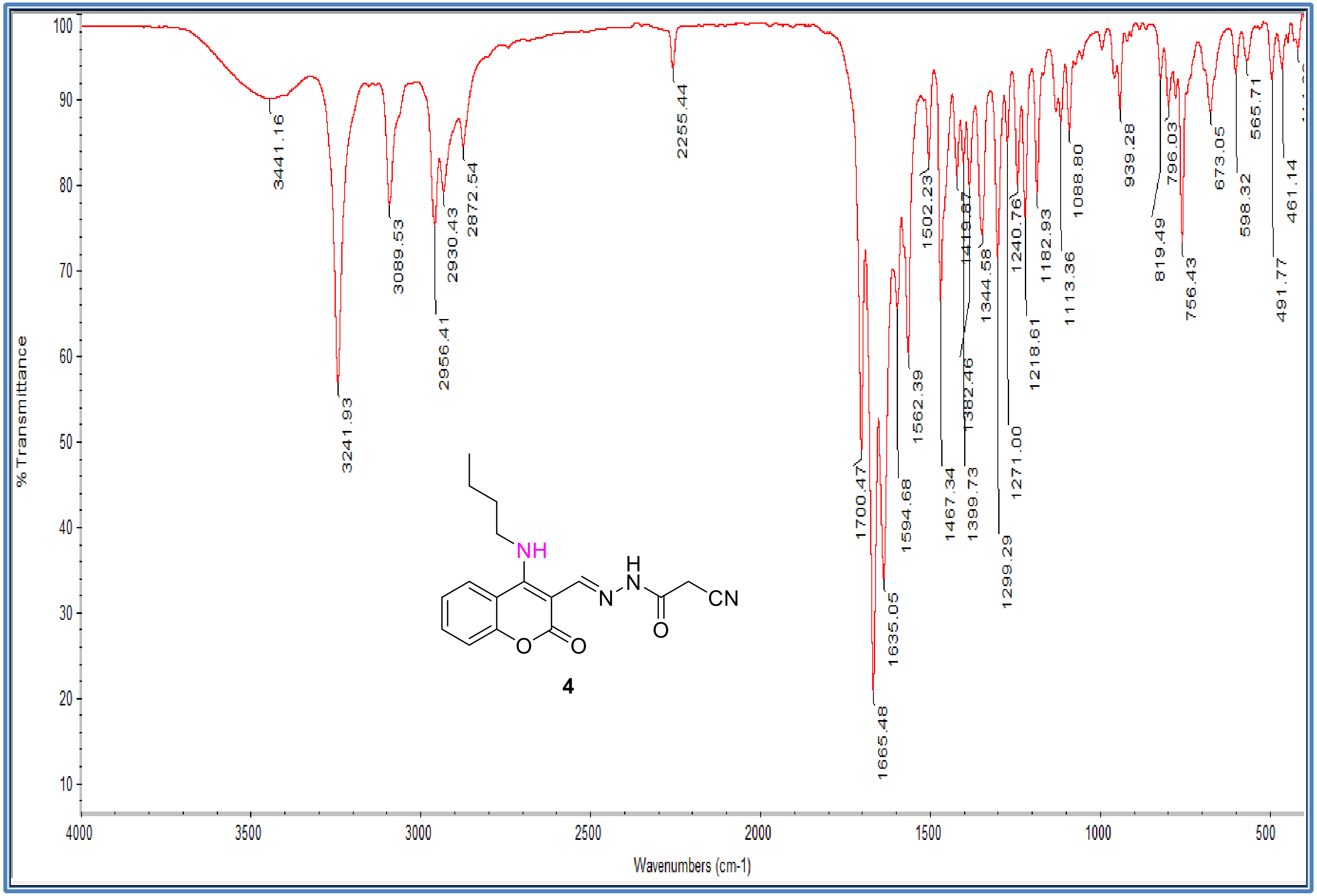

Spectroscopic data

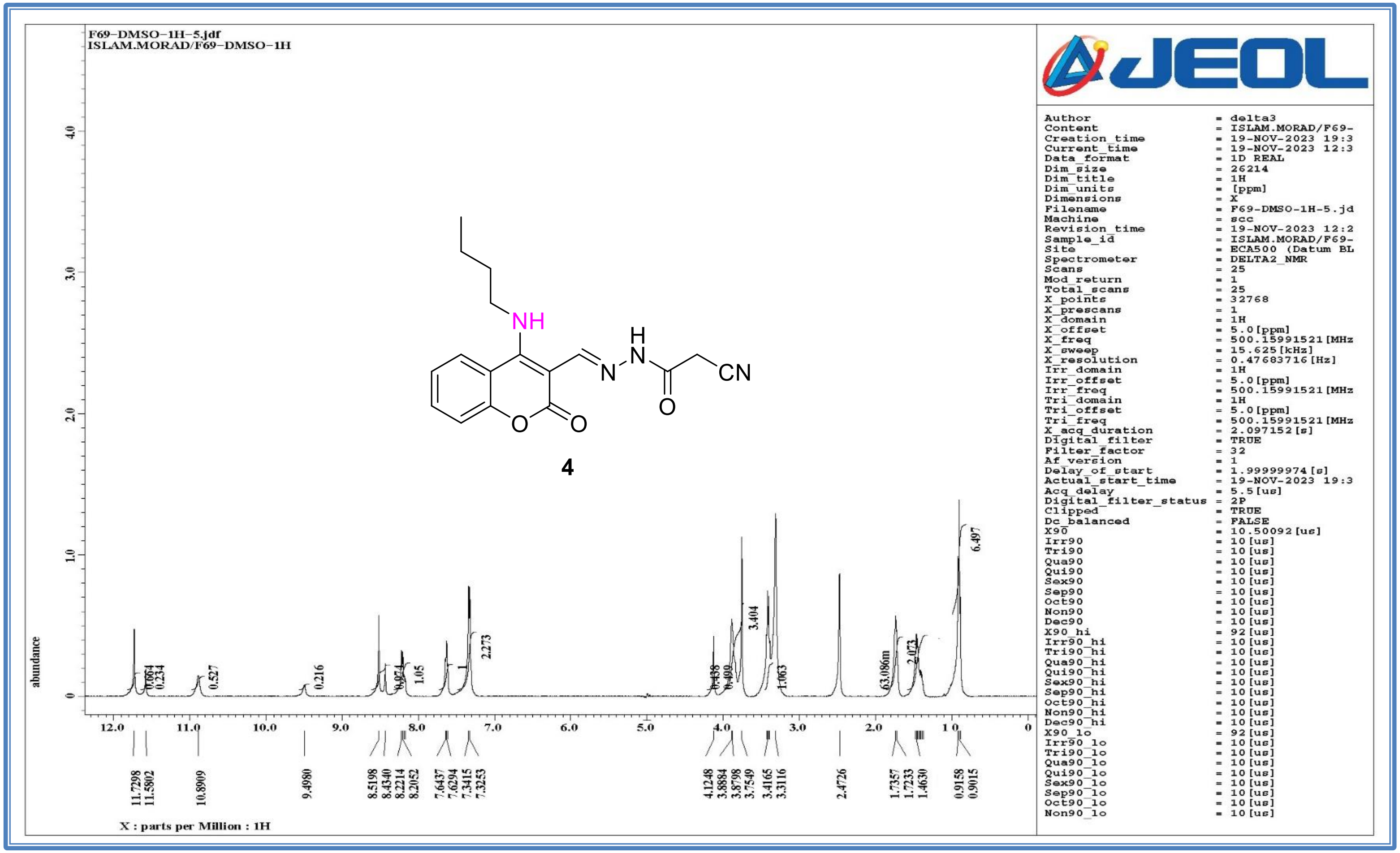

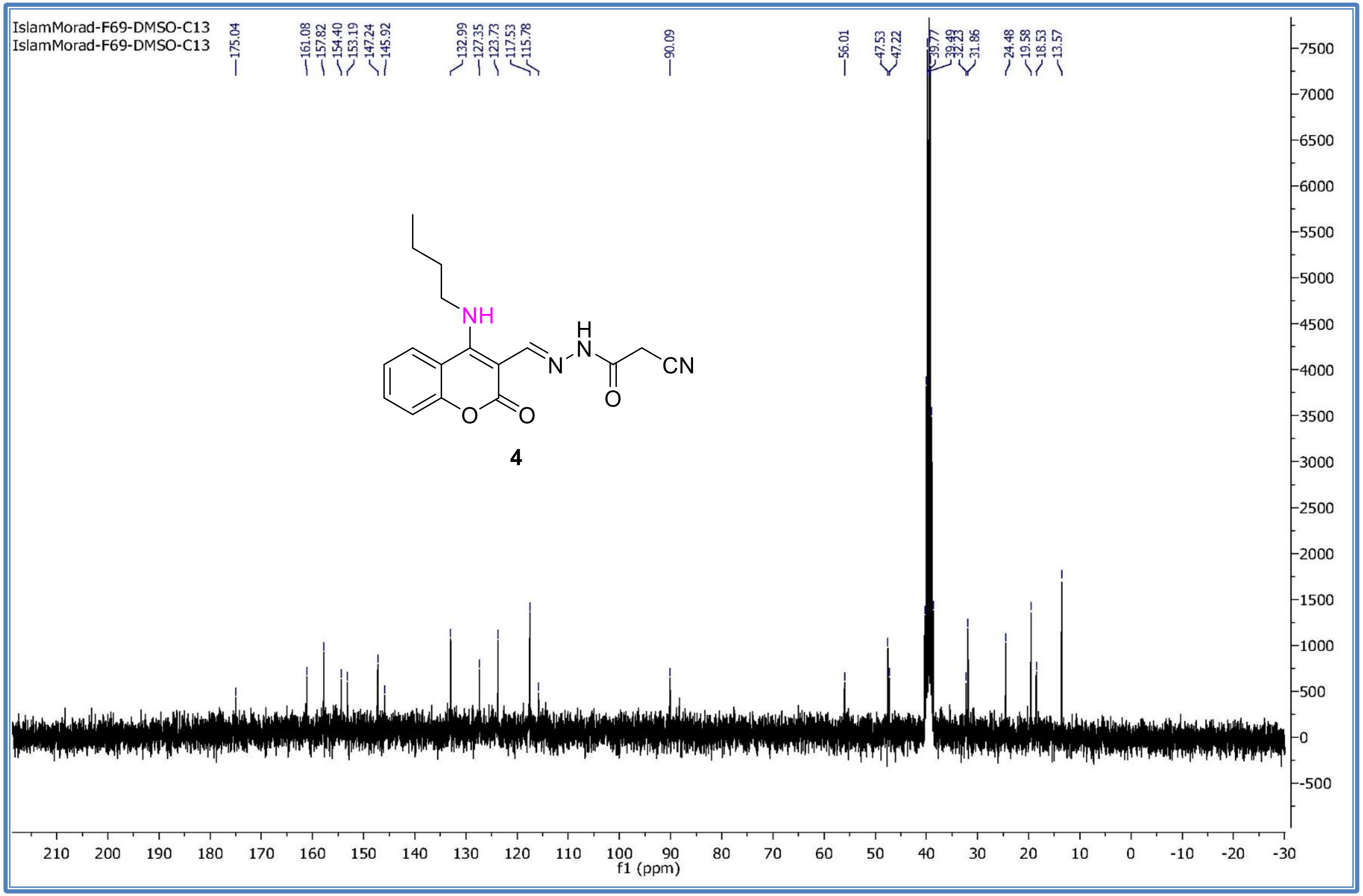

IslamMorad-F69-DMSO-C13
IslamMorad-F69-DMSO-C13
175.04
161.08
157.82
154.40
153.19
147.24
145.92
132.99
127.35
123.73
117.53
115.78
90.09
56.01
47.53
47.22
39.77
39.49
32.23
31.86
24.48
19.58
18.53
13.57
NH
H
N
N
CN
O
O
O
4
f1 (ppm)

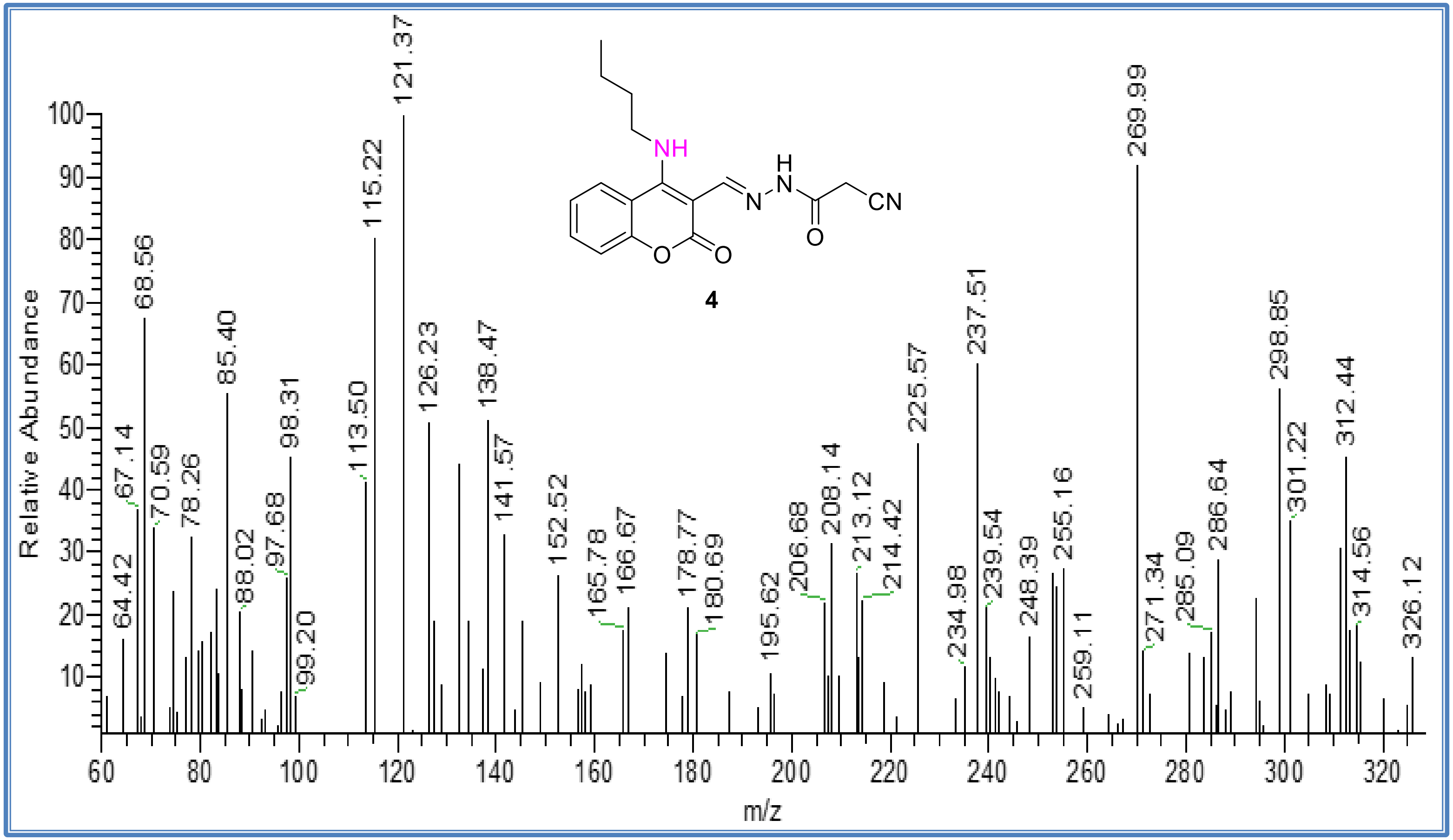
Relative Abundance
m/z
NH
H
N
N
CN
O
O
O
4
121.37
115.22
269.99
68.56
237.51
85.40
298.85
225.57
126.23
138.47
312.44
98.31
113.50
67.14
70.59
78.26
141.57
301.22
152.52
286.64
255.16
97.68
88.02
208.14
213.12
214.42
166.67
178.77
180.69
206.68
239.54
165.78
195.62
248.39
285.09
64.42
314.56
326.12
234.98
271.34
99.20
259.11

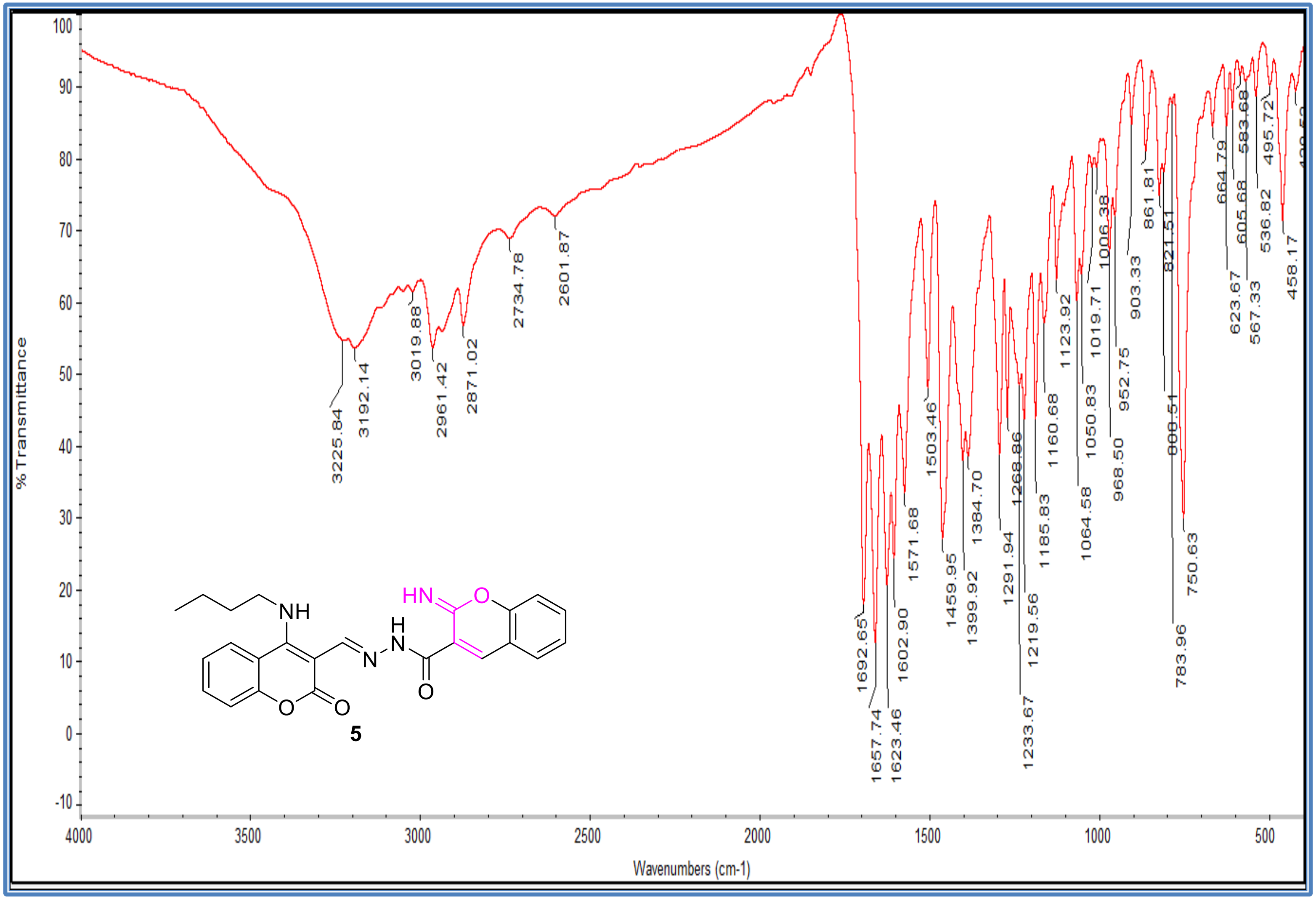
% Transmittance
Wavenumbers (cm-1)
3225.84
3192.14
3019.88
2961.42
2871.02
2734.78
2601.87
1692.65
1657.74
1623.46
1602.90
1571.68
1503.46
1459.95
1399.92
1384.70
1291.94
1268.86
1233.67
1219.56
1185.83
1160.68
1123.92
1064.58
1050.83
1019.71
1006.38
968.50
952.75
903.33
861.81
821.51
808.51
783.96
750.63
664.79
623.67
605.68
583.68
567.33
536.82
495.72
458.17
NH
HN
O
H
N
N
O
O
O
5

Spectroscopic data

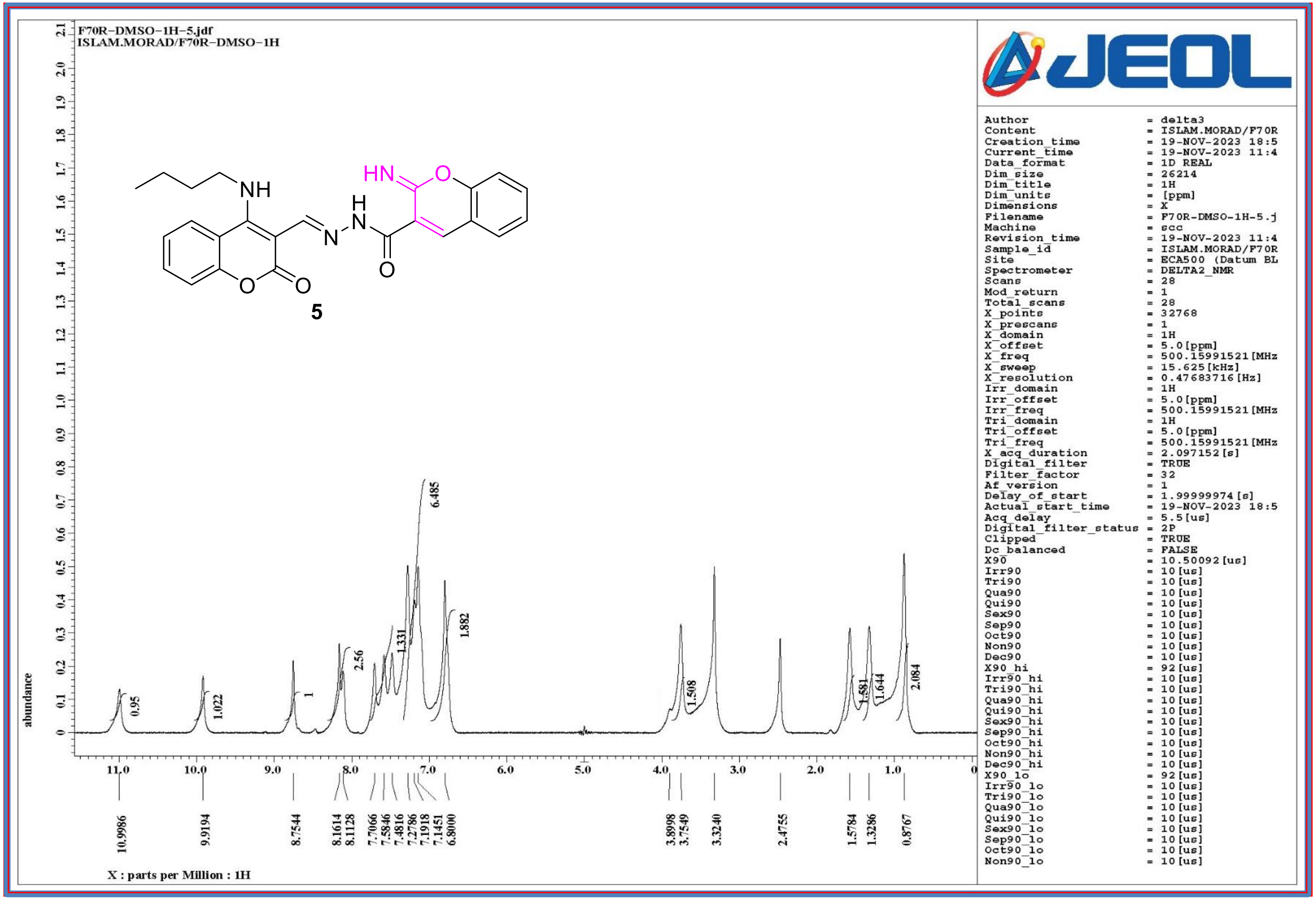

Spectroscopic data

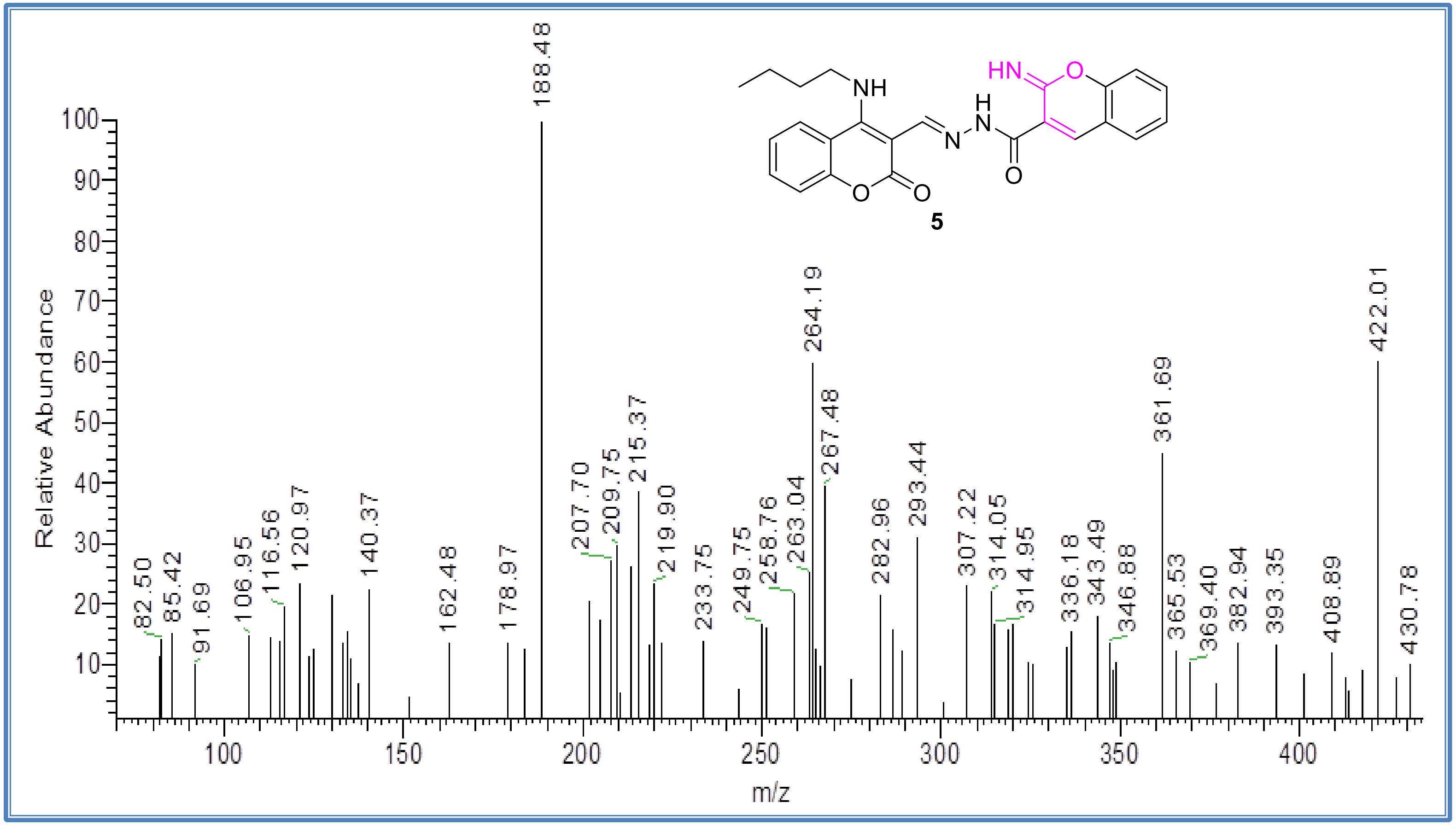

Spectroscopic data

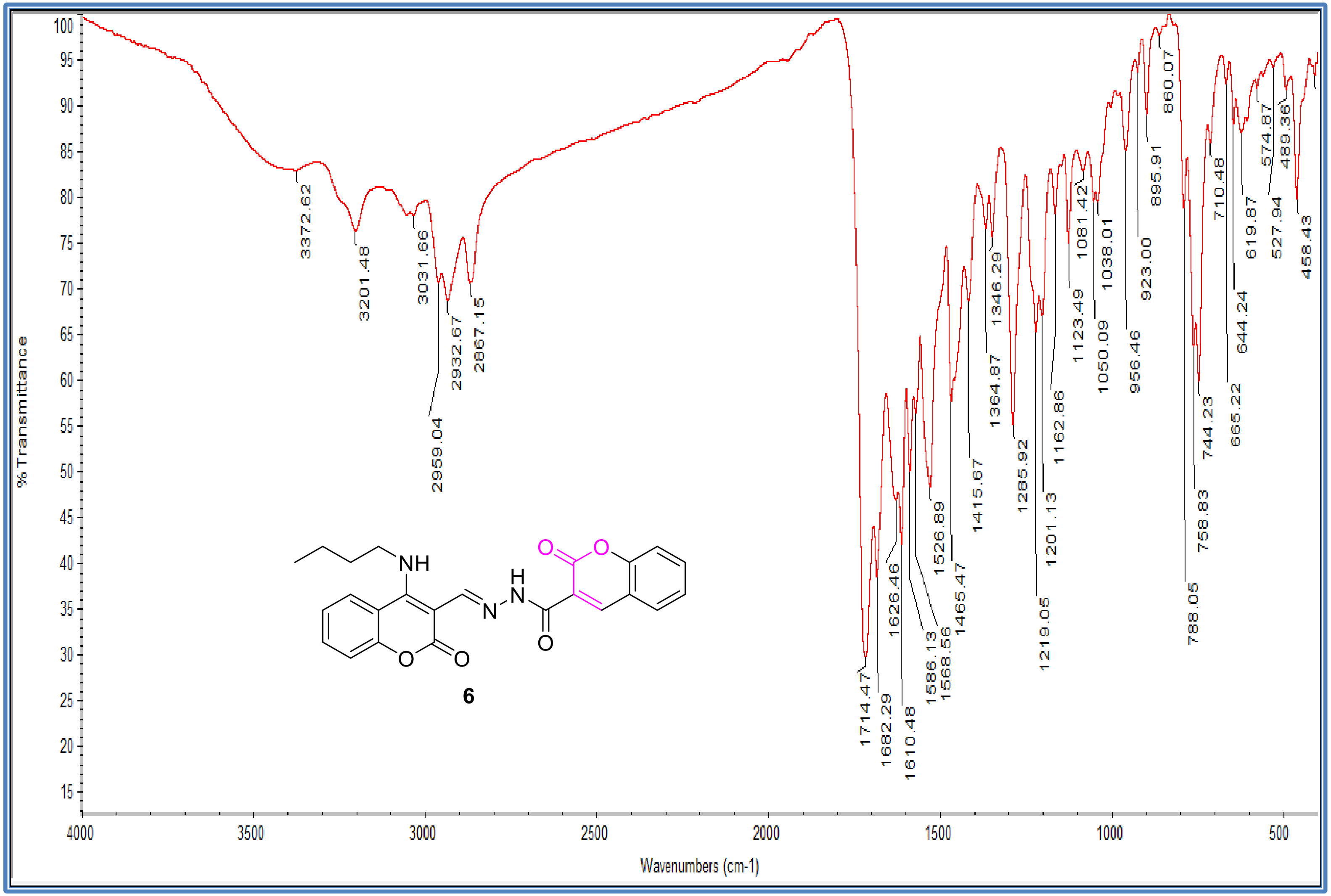

Spectroscopic data

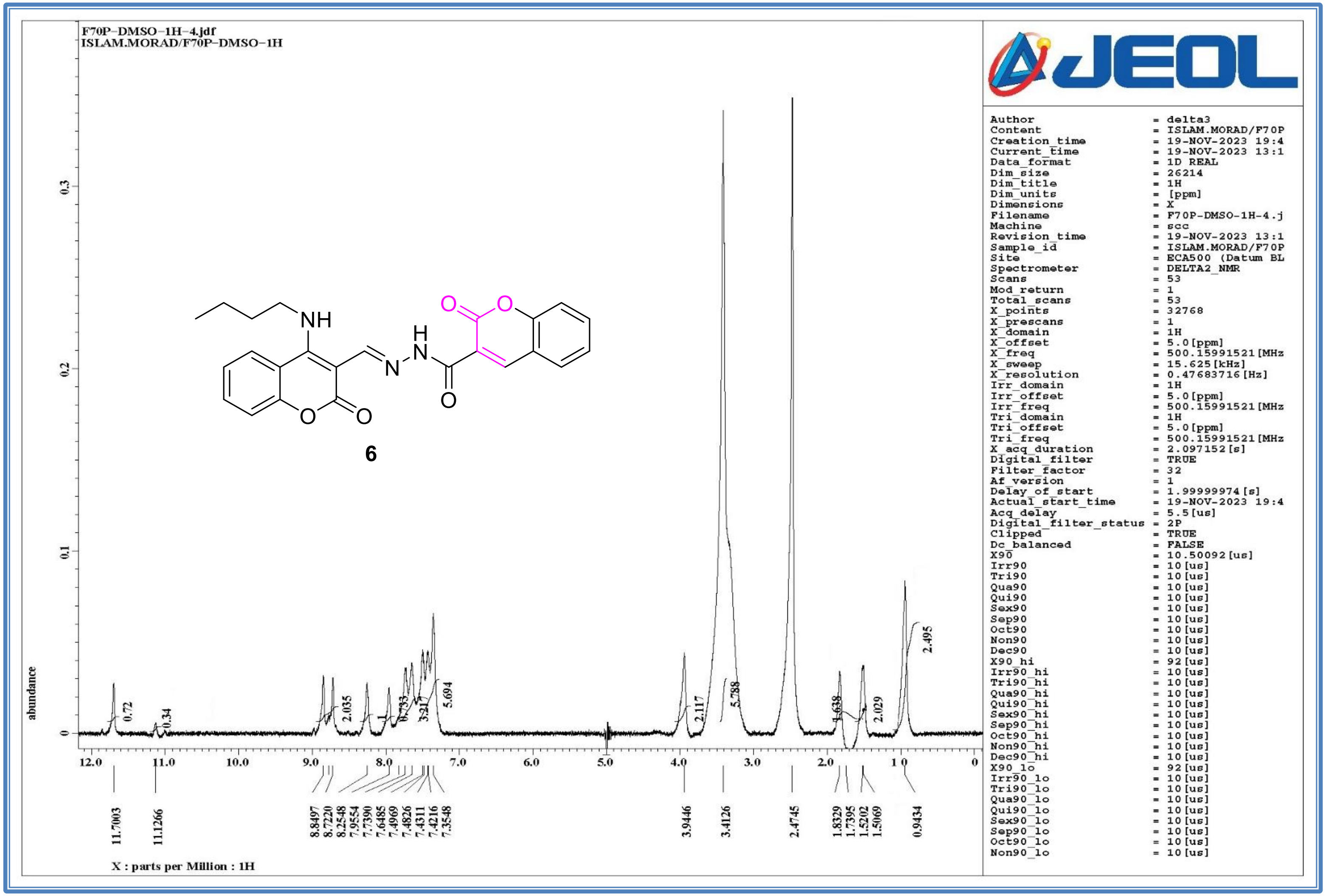

Spectroscopic data

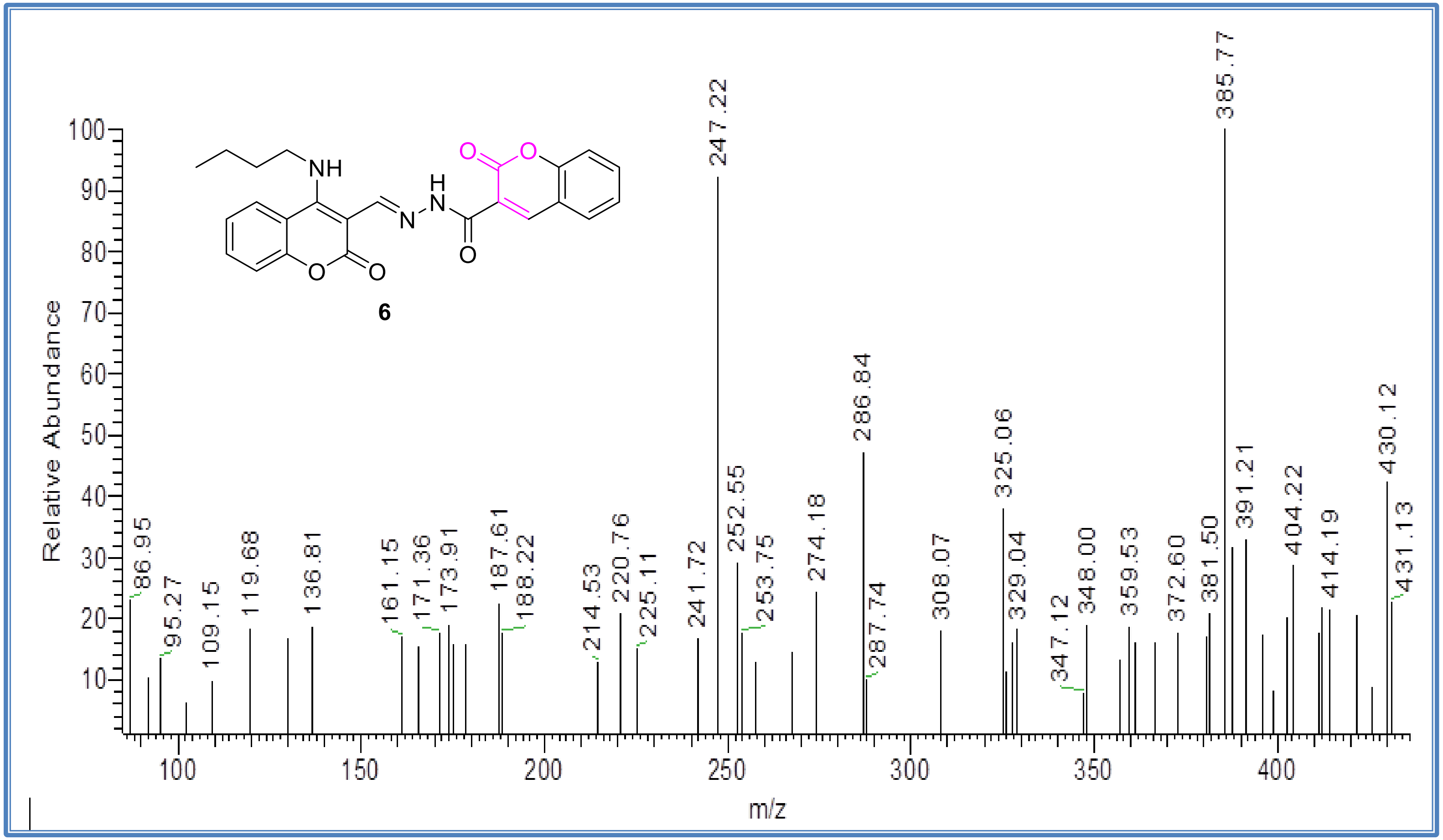

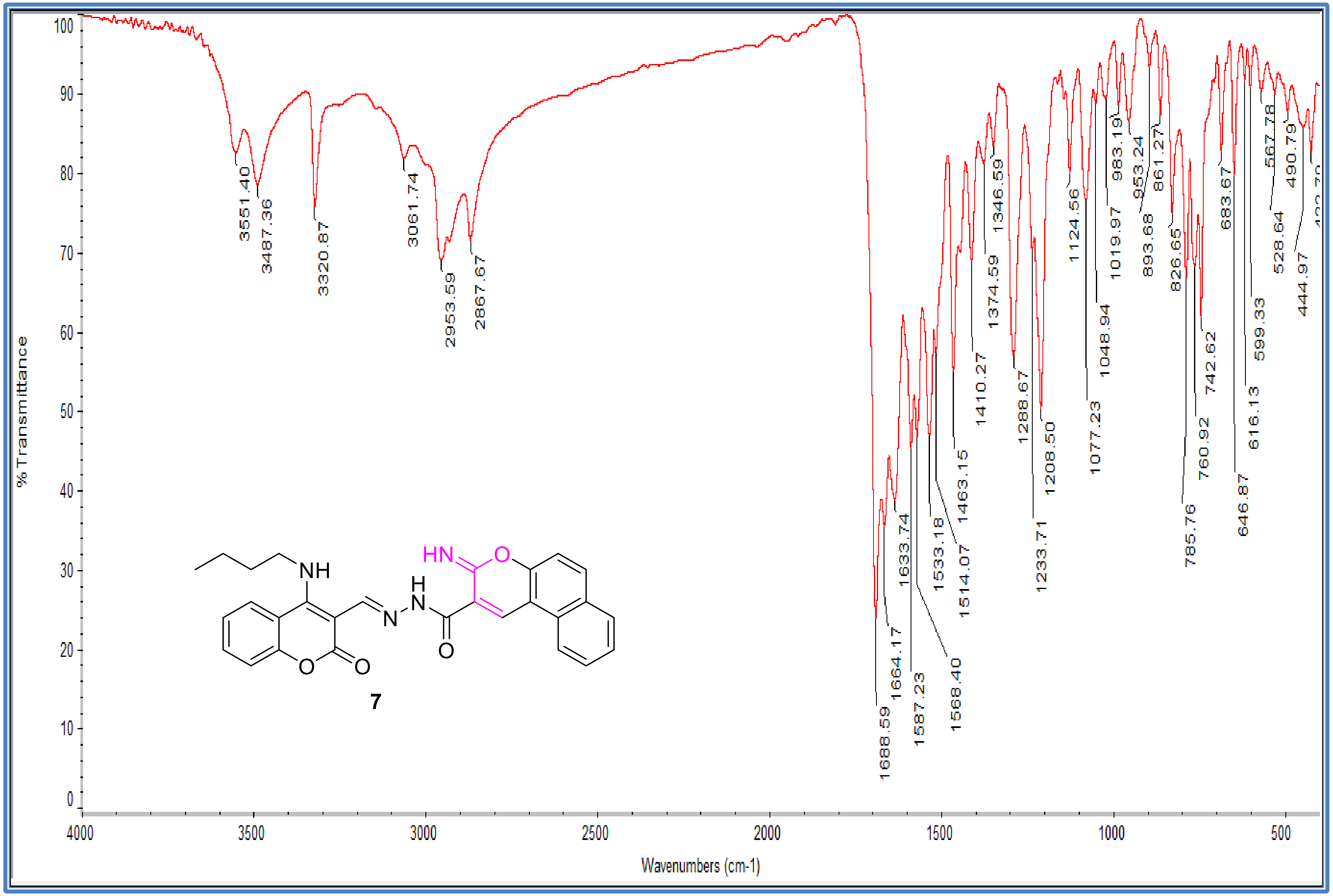

%Transmittance
Wavenumbers (cm-1)
100
90
80
70
60
50
40
30
20
10
0
4000
3500
3000
2500
2000
1500
1000
500
3551.40
3487.36
3320.87
3061.74
2953.59
2867.67
1688.59
1664.17
1633.74
1587.23
1568.40
1533.18
1514.07
1463.15
1410.27
1374.59
1346.59
1288.67
1233.71
1208.50
1124.56
1077.23
1048.94
1019.97
983.19
953.24
893.68
861.27
826.65
785.76
760.92
742.62
683.67
646.87
616.13
599.33
567.78
528.64
490.79
444.97
NH
HN
O
H
N
N
O
O
O
7

Spectroscopic data

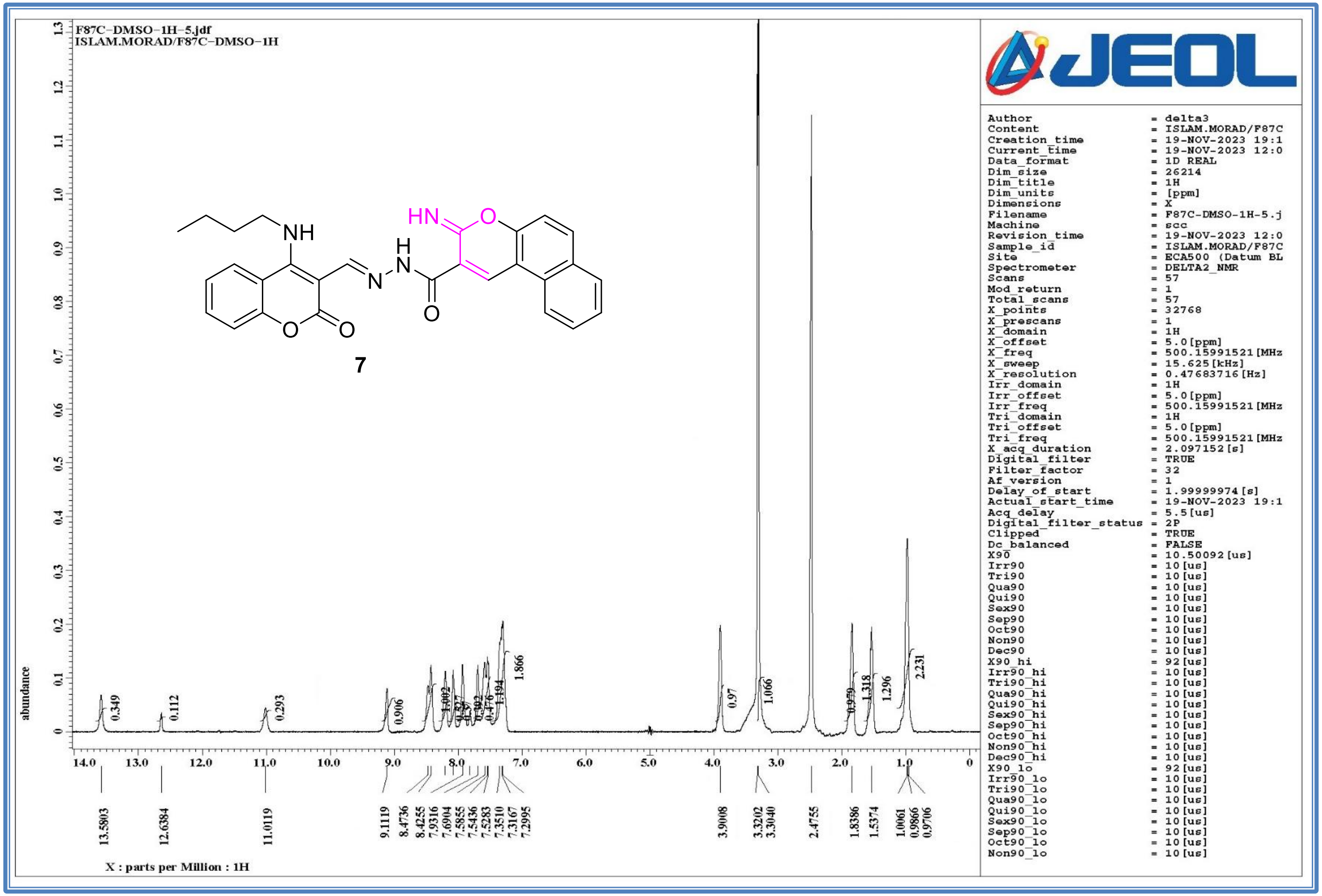

Spectroscopic data

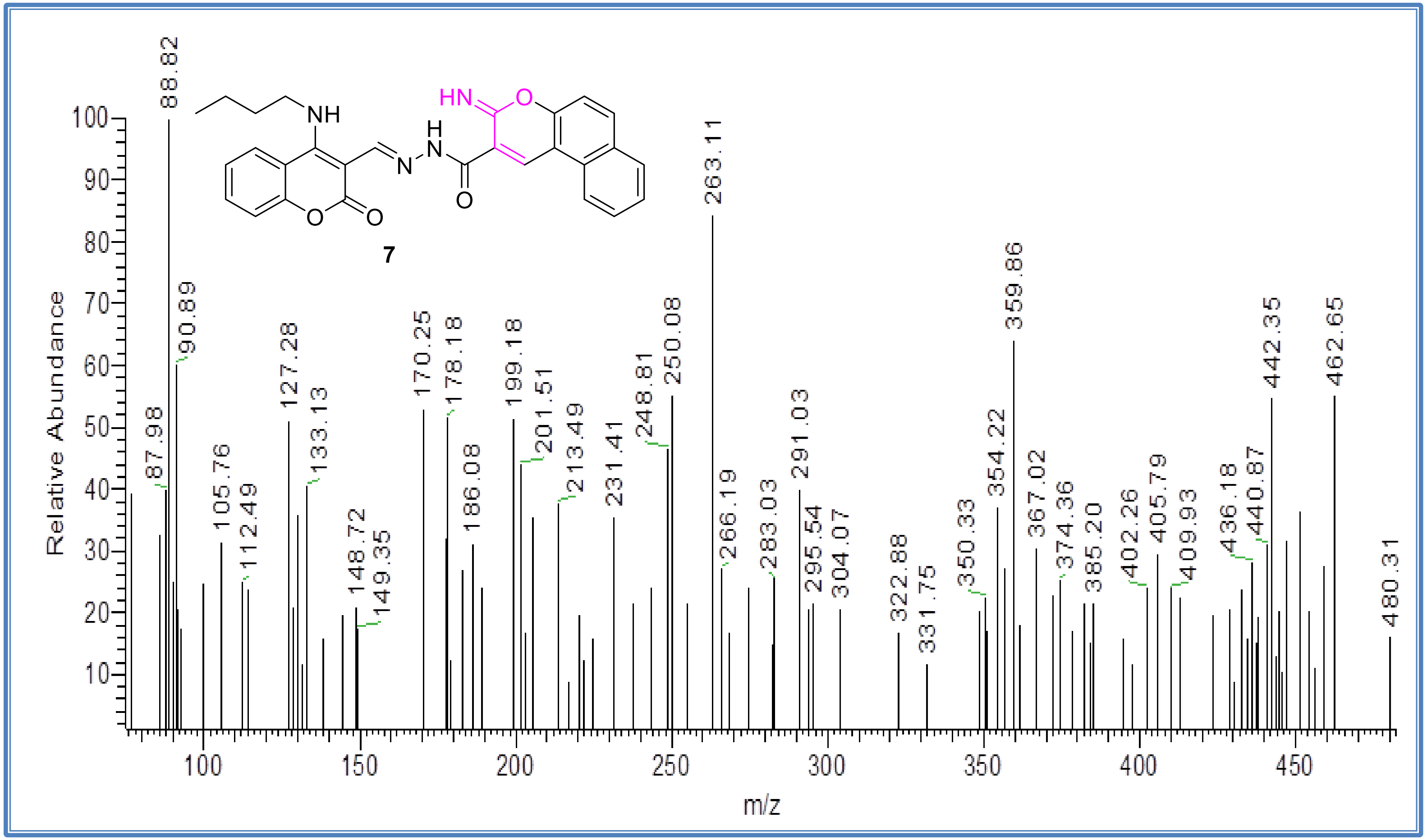

Spectroscopic data

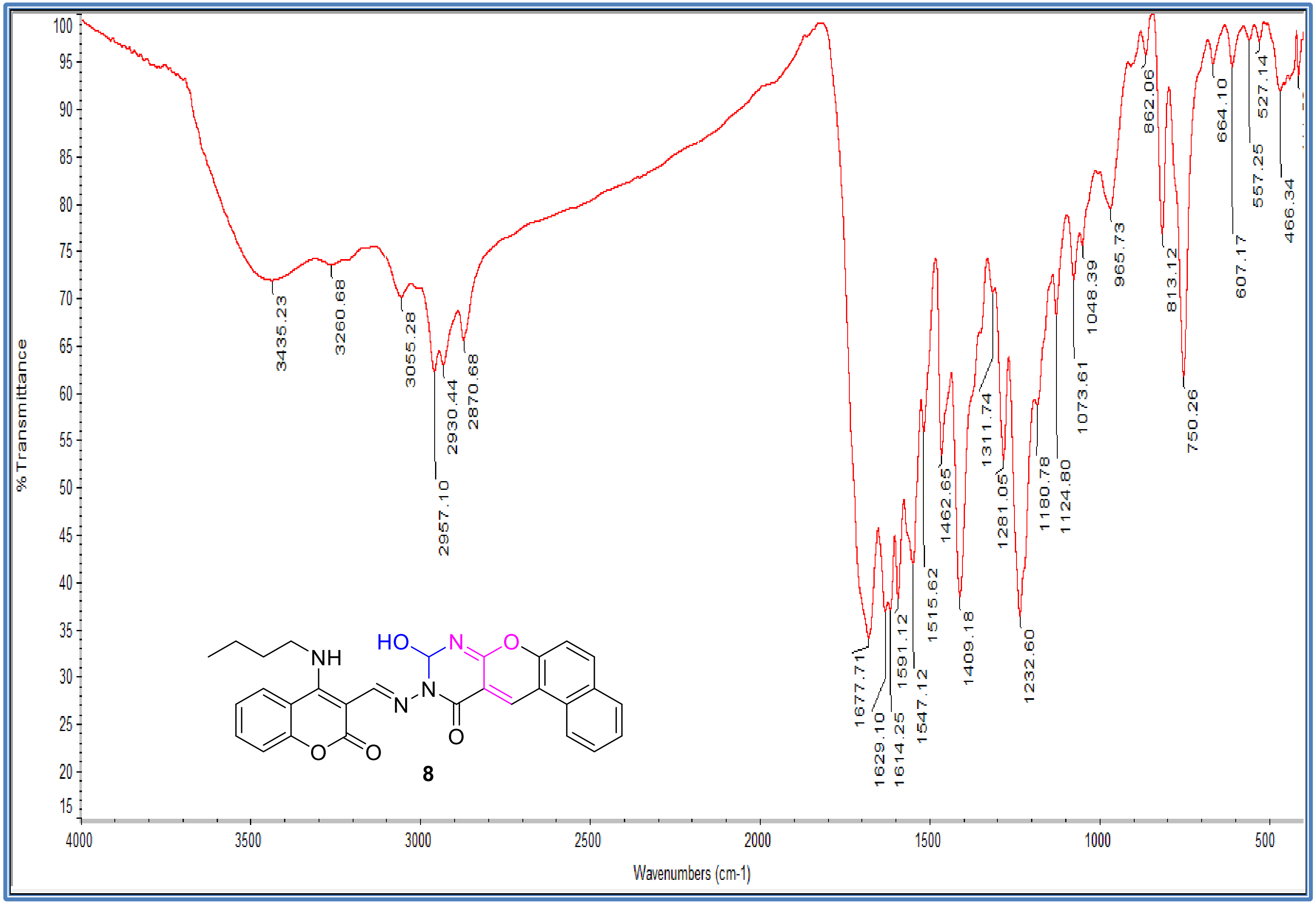

Spectroscopic data

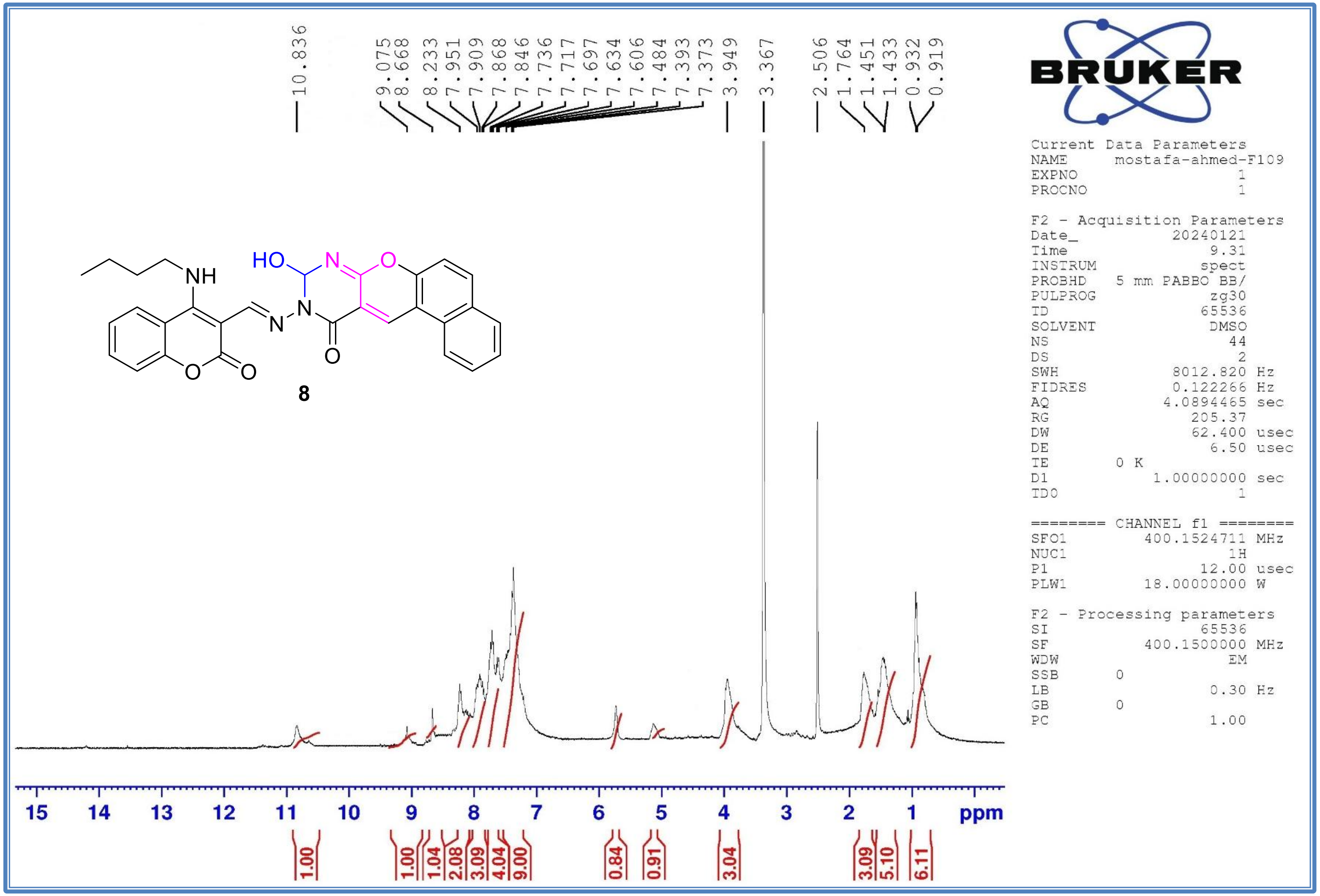

8

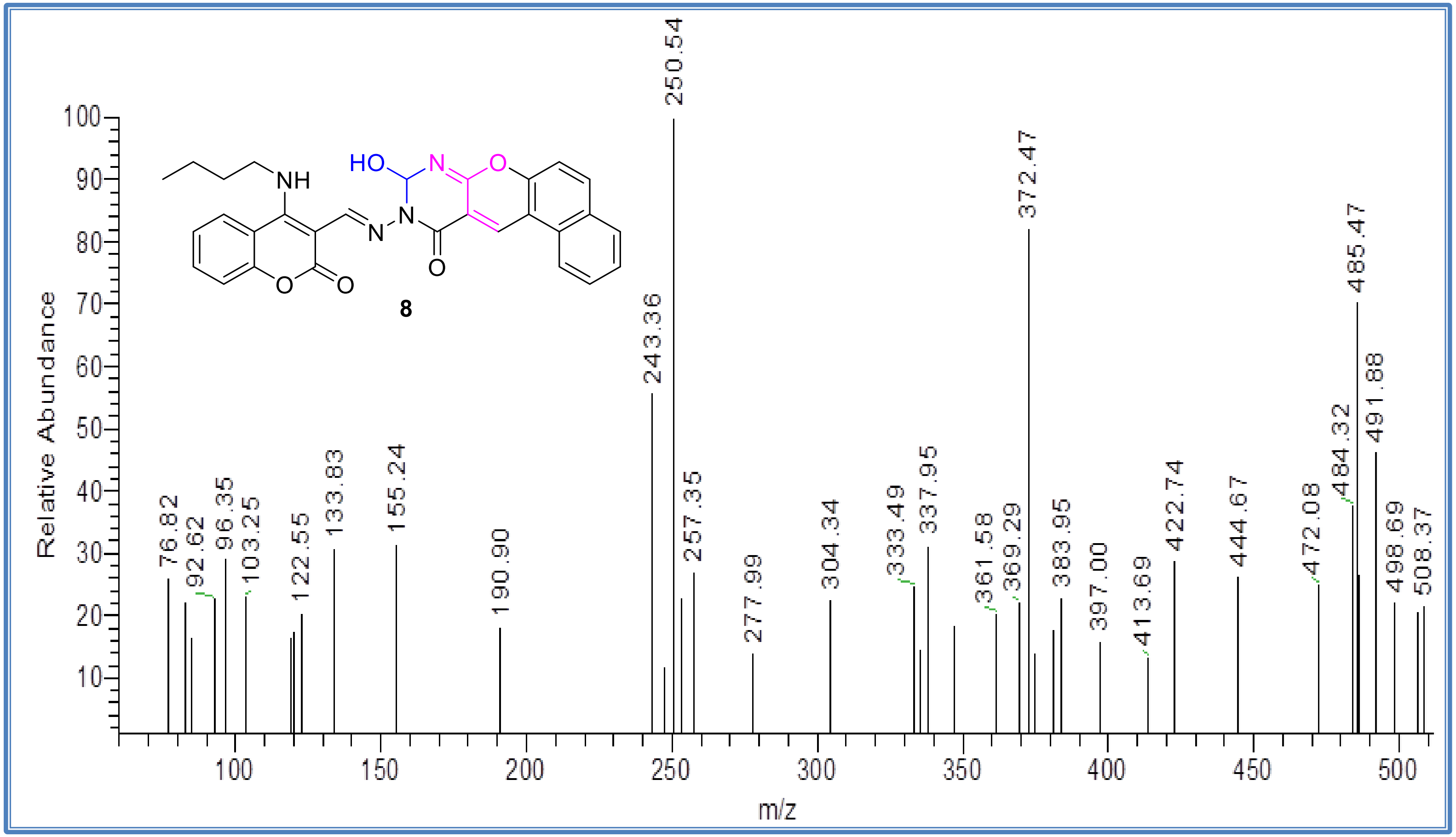

250.54
372.47
485.47
491.88
243.36
484.32
76.82
92.62
96.35
103.25
122.55
133.83
155.24
190.90
257.35
277.99
304.34
333.49
337.95
361.58
369.29
383.95
397.00
413.69
422.74
444.67
472.08
498.69
508.37
HO
N
O
NH
N
N
O
O
O
8
Relative Abundance
m/z
100
90
80
70
60
50
40
30
20
10
150
200
250
300
350
400
450
500

Spectroscopic data

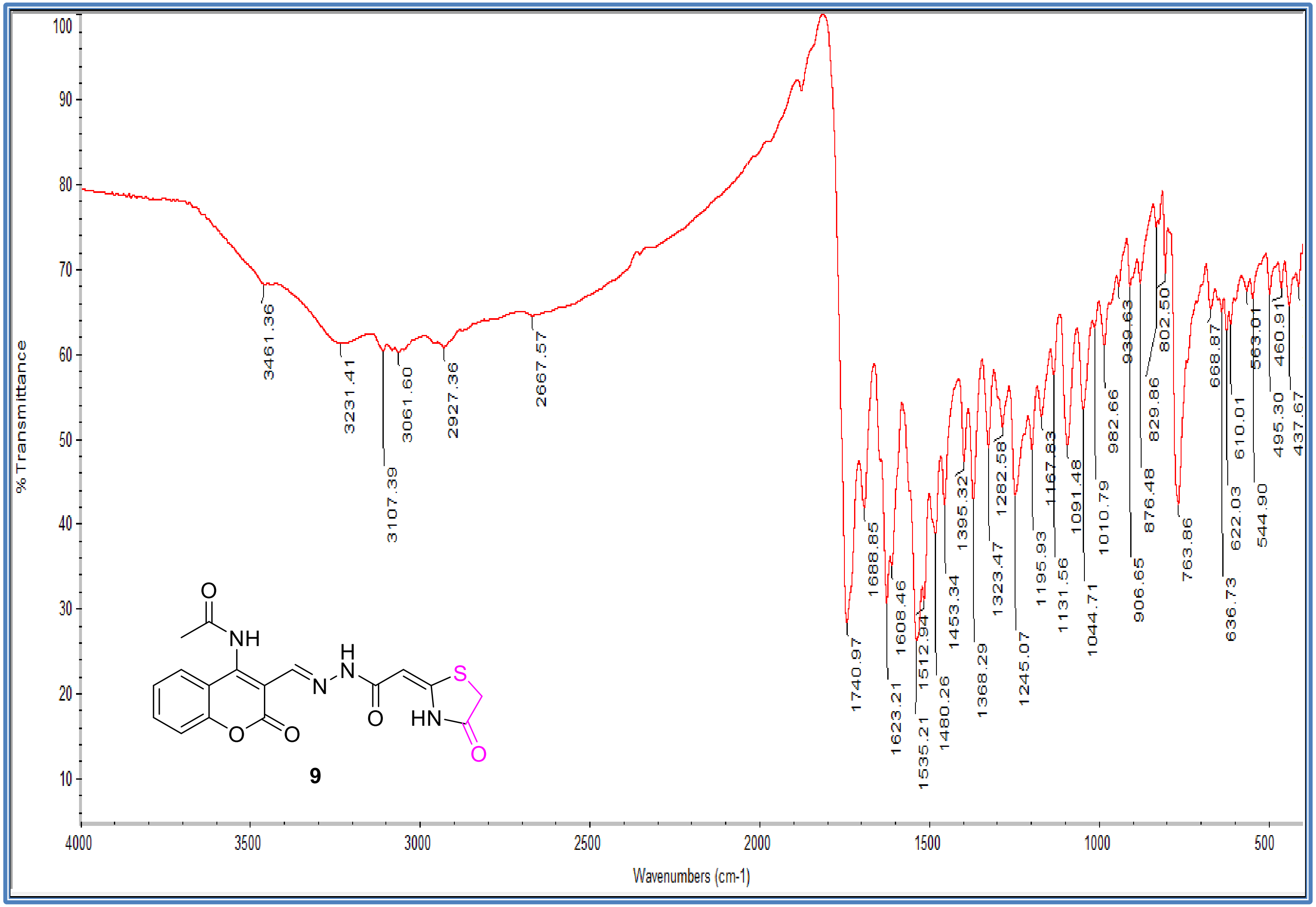

9

Spectroscopic data

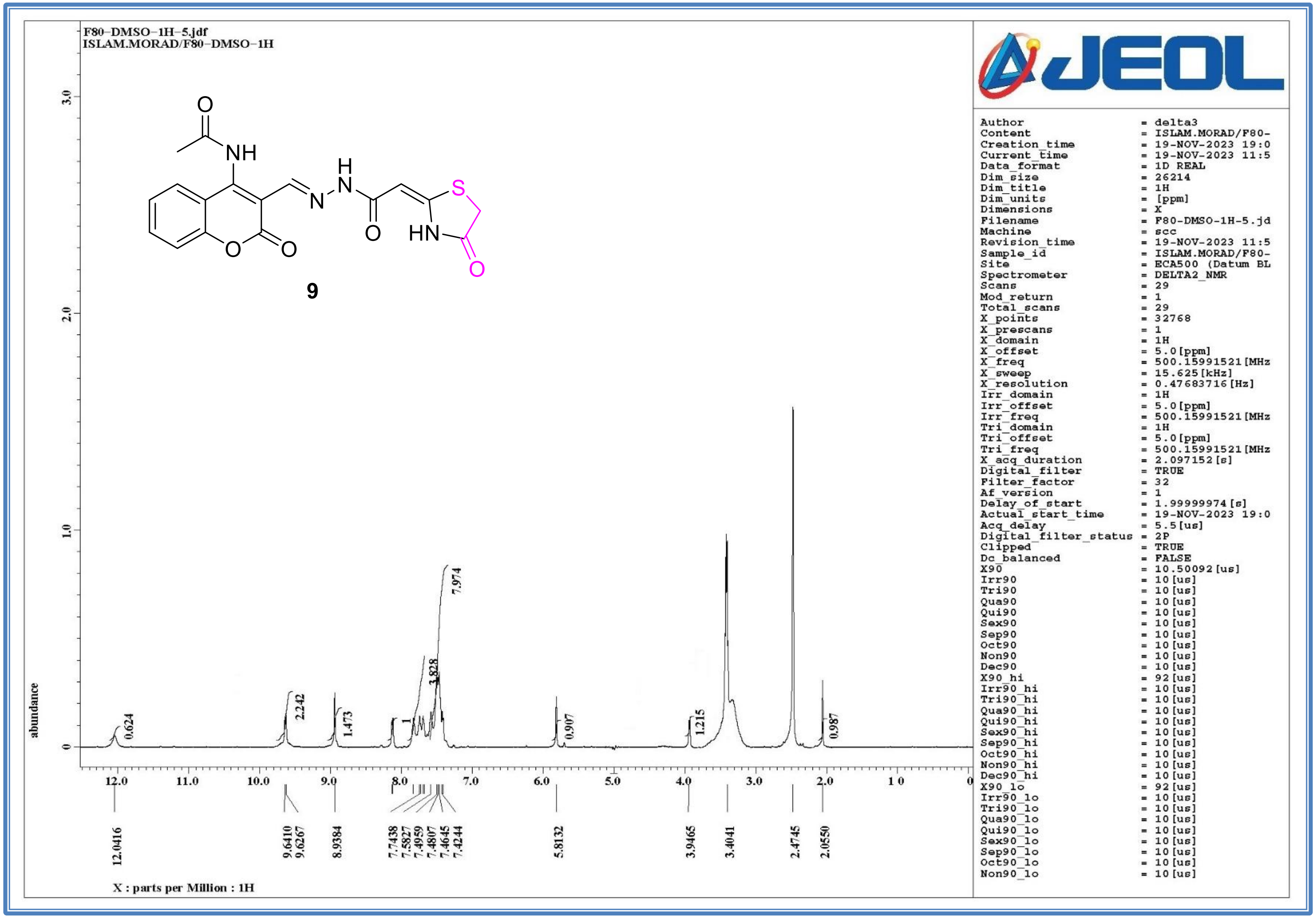

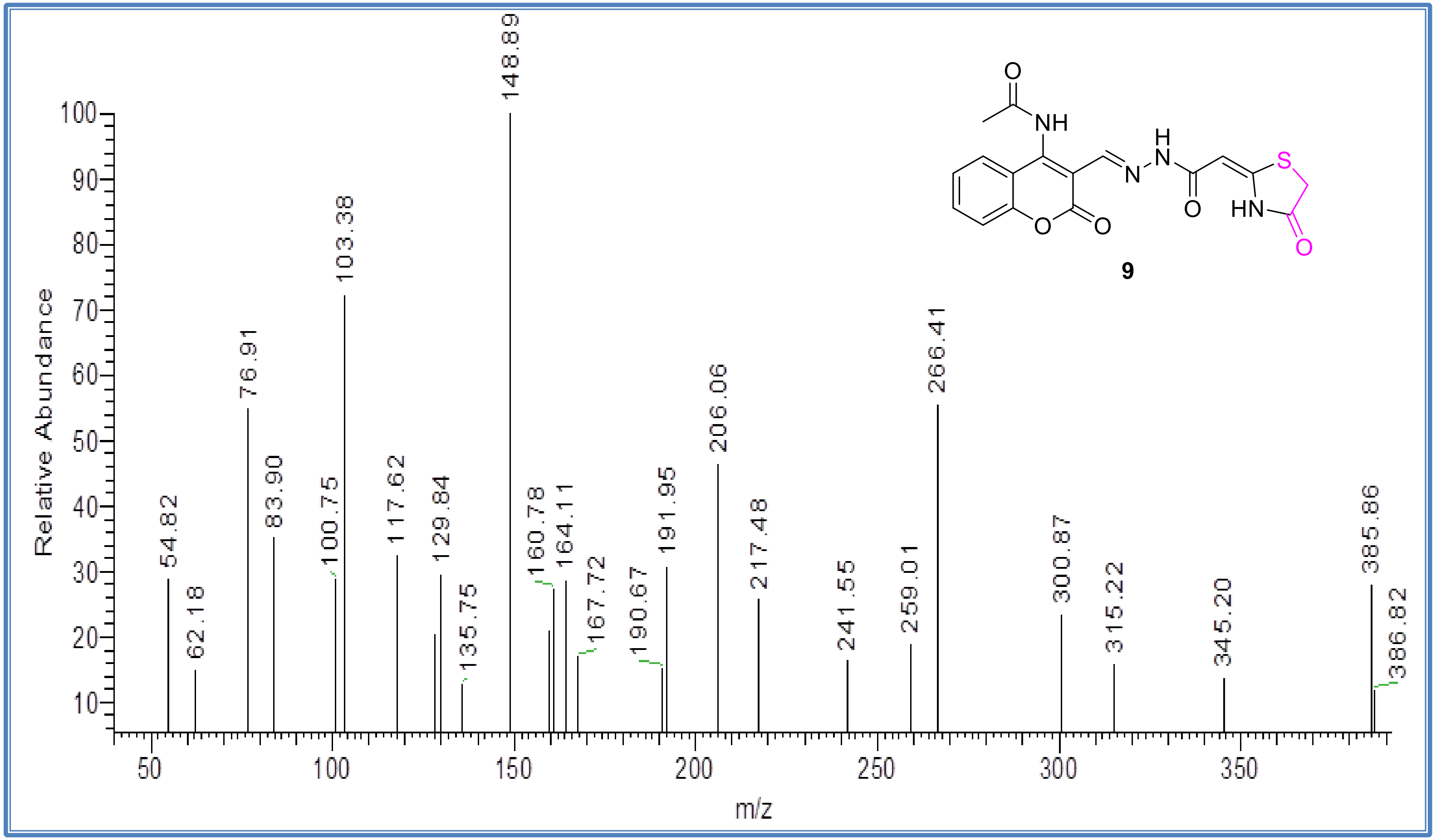
148.89
103.38
76.91
83.90
100.75
117.62
129.84
135.75
160.78
164.11
167.72
190.67
191.95
206.06
217.48
241.55
259.01
266.41
300.87
315.22
345.20
385.86
386.82
54.82
62.18
Relative Abundance
m/z
9

Spectroscopic data

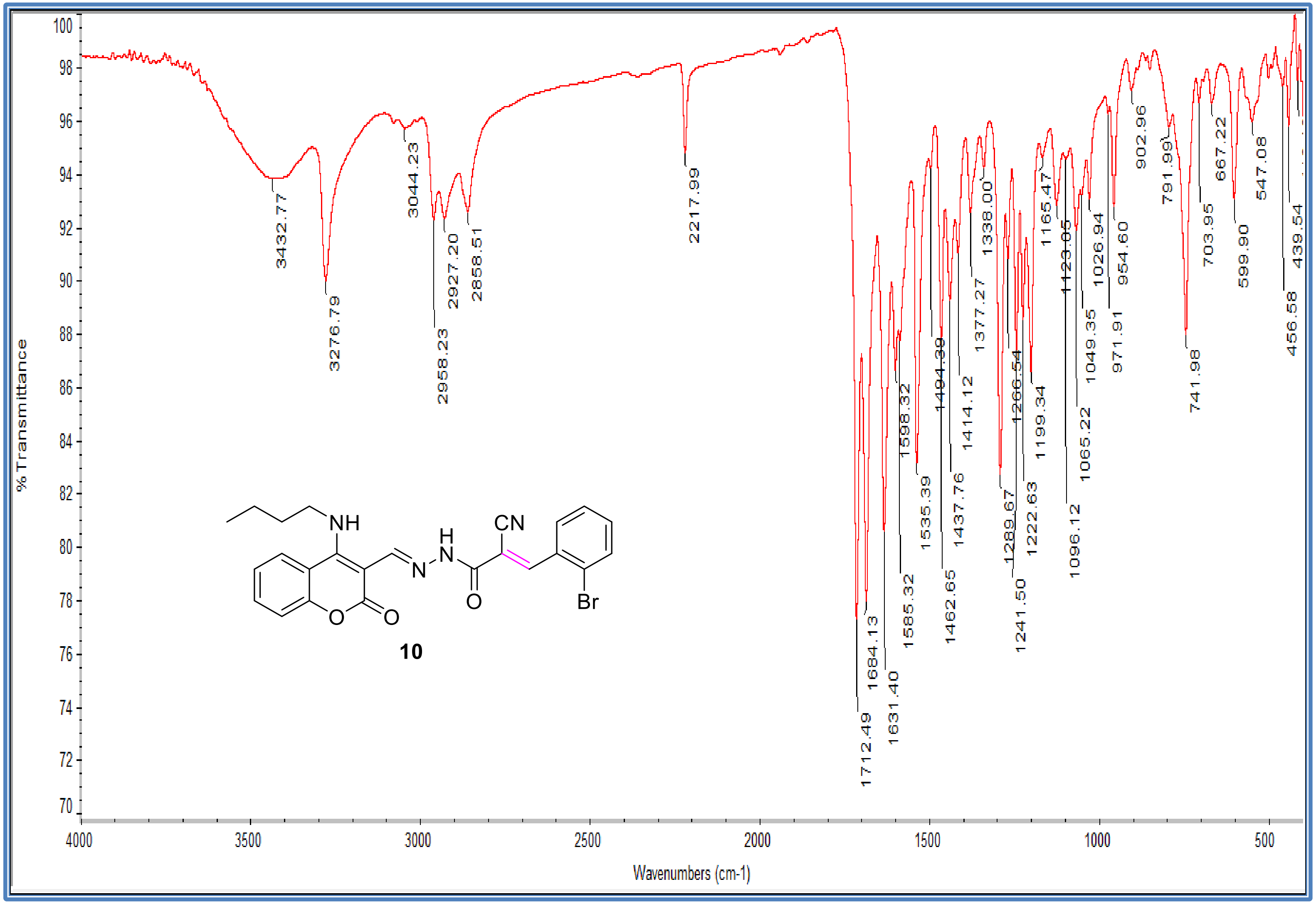

Spectroscopic data

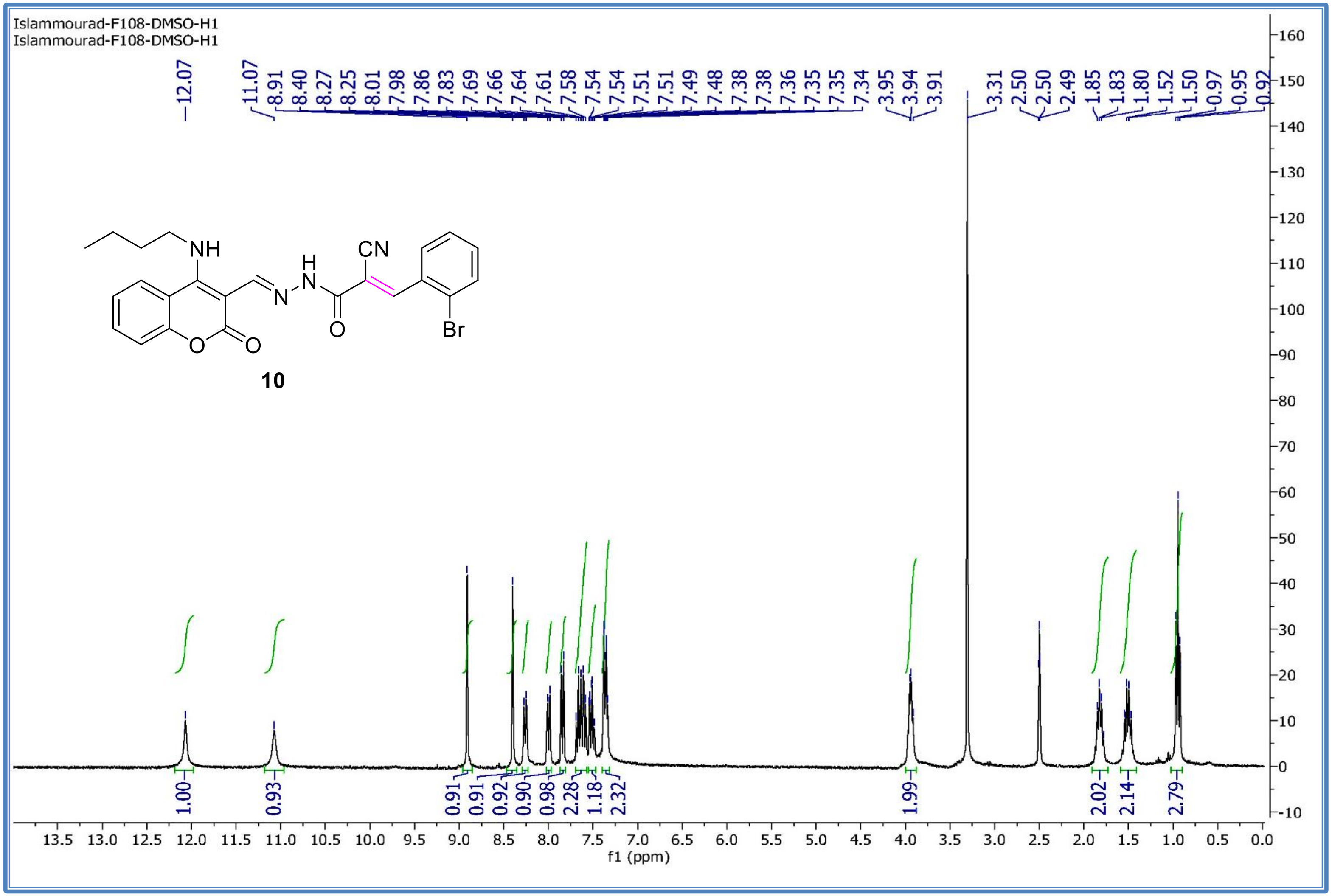

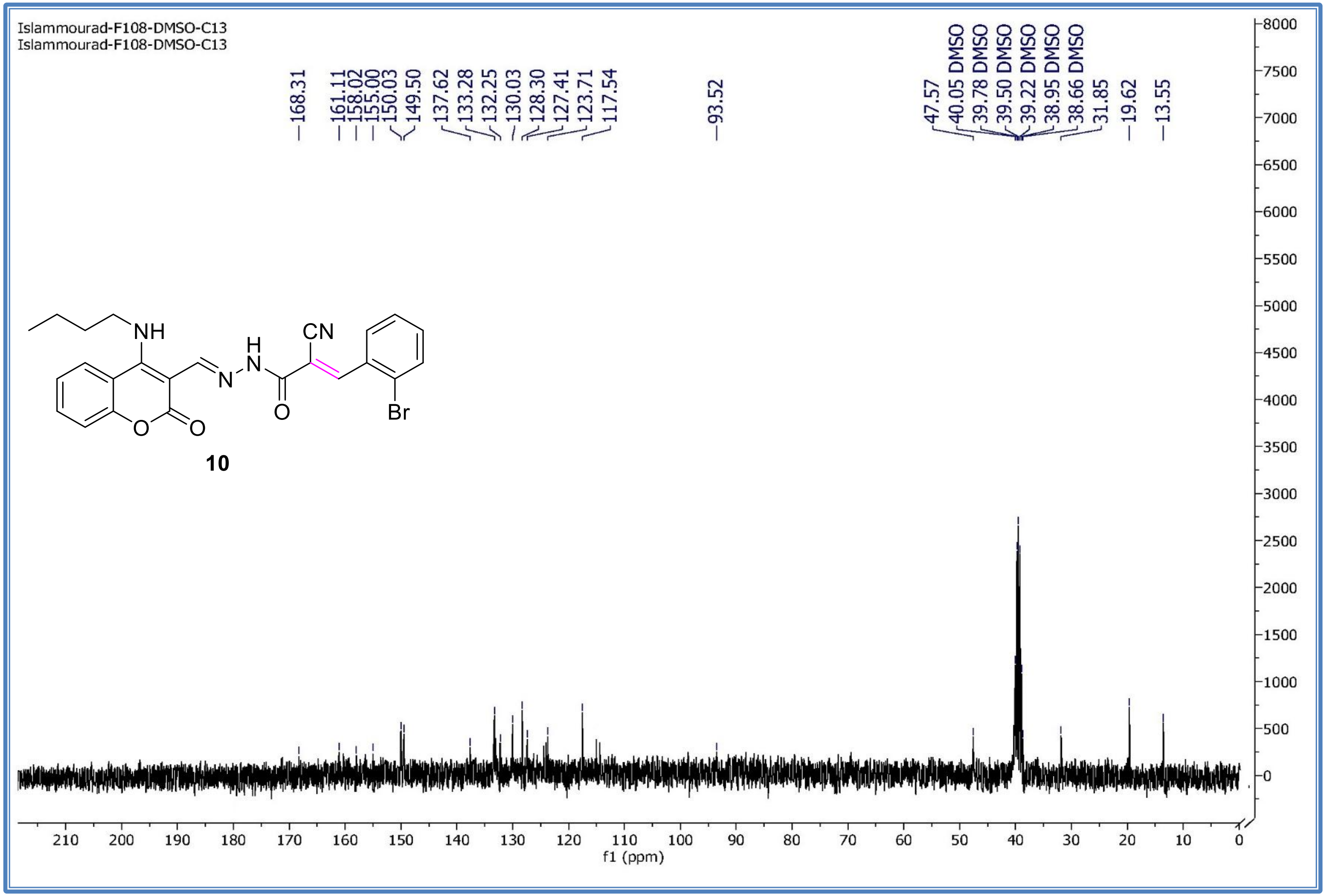
Islammourad-F108-DMSO-C13
Islammourad-F108-DMSO-C13
168.31
161.11
158.02
155.00
150.03
149.50
137.62
133.28
132.25
130.03
128.30
127.41
123.71
117.54
93.52
47.57
40.05 DMSO
39.78 DMSO
39.50 DMSO
39.22 DMSO
38.95 DMSO
38.66 DMSO
31.85
19.62
13.55
NH
H
N
CN
N
O
O
O
Br
10
8000
7500
7000
6500
6000
5500
5000
4500
4000
3500
3000
2500
2000
1500
1000
500
0
210
200
190
180
170
160
150
140
130
120
110
100
90
80
70
60
50
40
30
20
10
0
f1 (ppm)

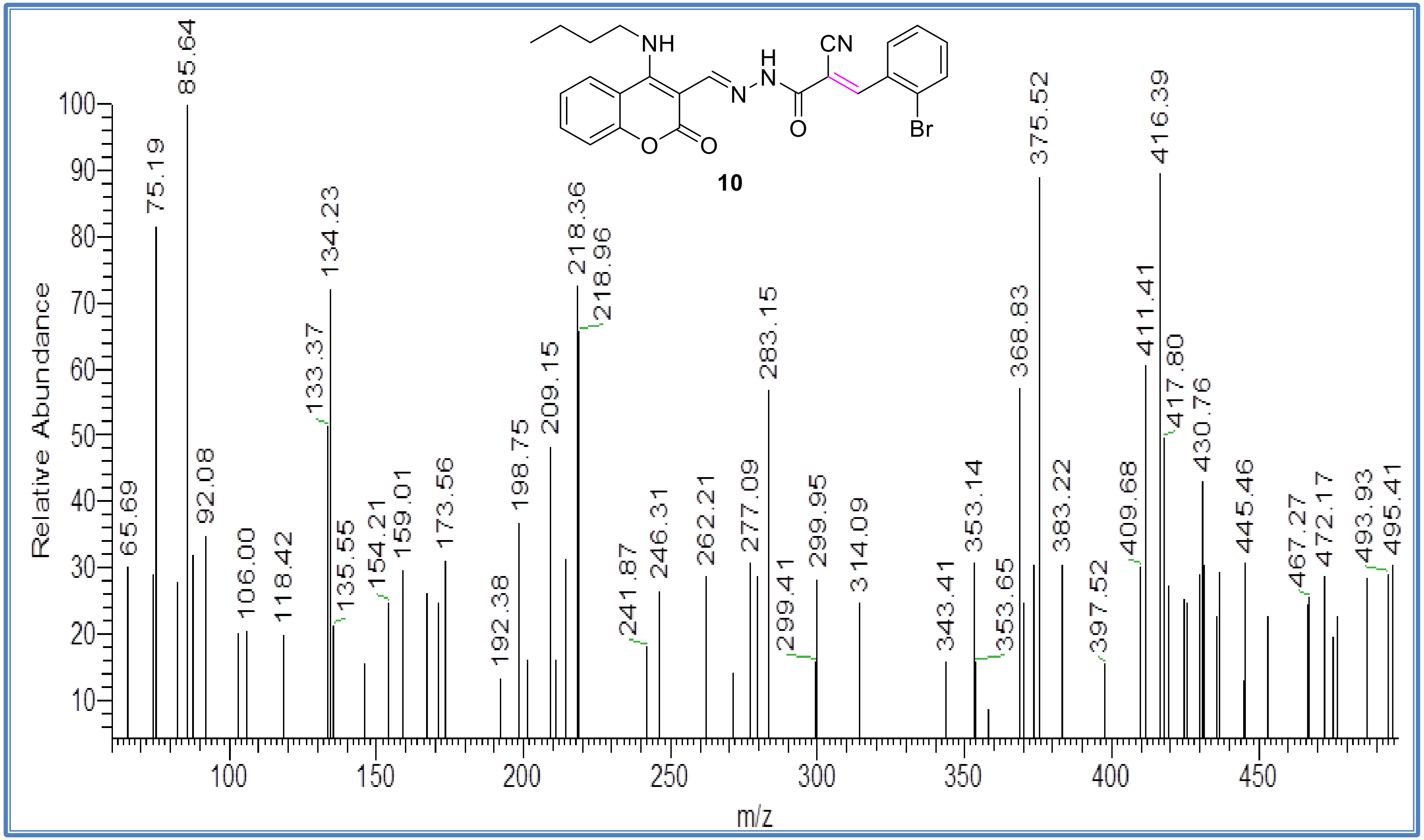
NH
H
N
N
CN
O
O
O
Br
10
Relative Abundance
m/z
100
90
80
70
60
50
40
30
20
10
150
200
250
300
350
400
450
85.64
75.19
134.23
218.36
218.96
133.37
283.15
375.52
416.39
368.83
411.41
417.80
430.76
209.15
198.75
92.08
65.69
106.00
118.42
135.55
154.21
159.01
173.56
192.38
241.87
246.31
262.21
277.09
299.41
299.95
314.09
343.41
353.14
353.65
383.22
397.52
409.68
445.46
467.27
472.17
493.93
495.41

Spectroscopic data

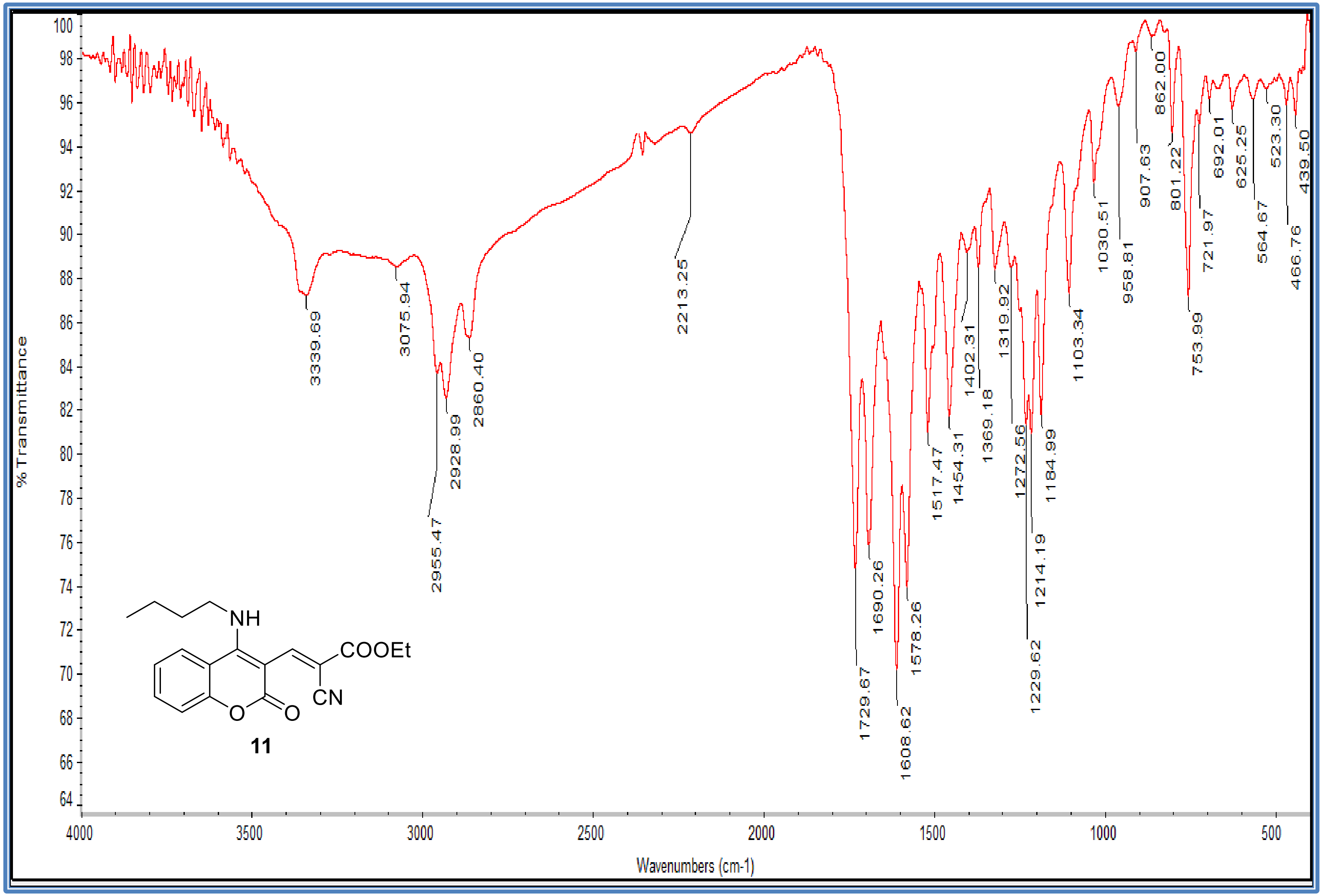

Spectroscopic data

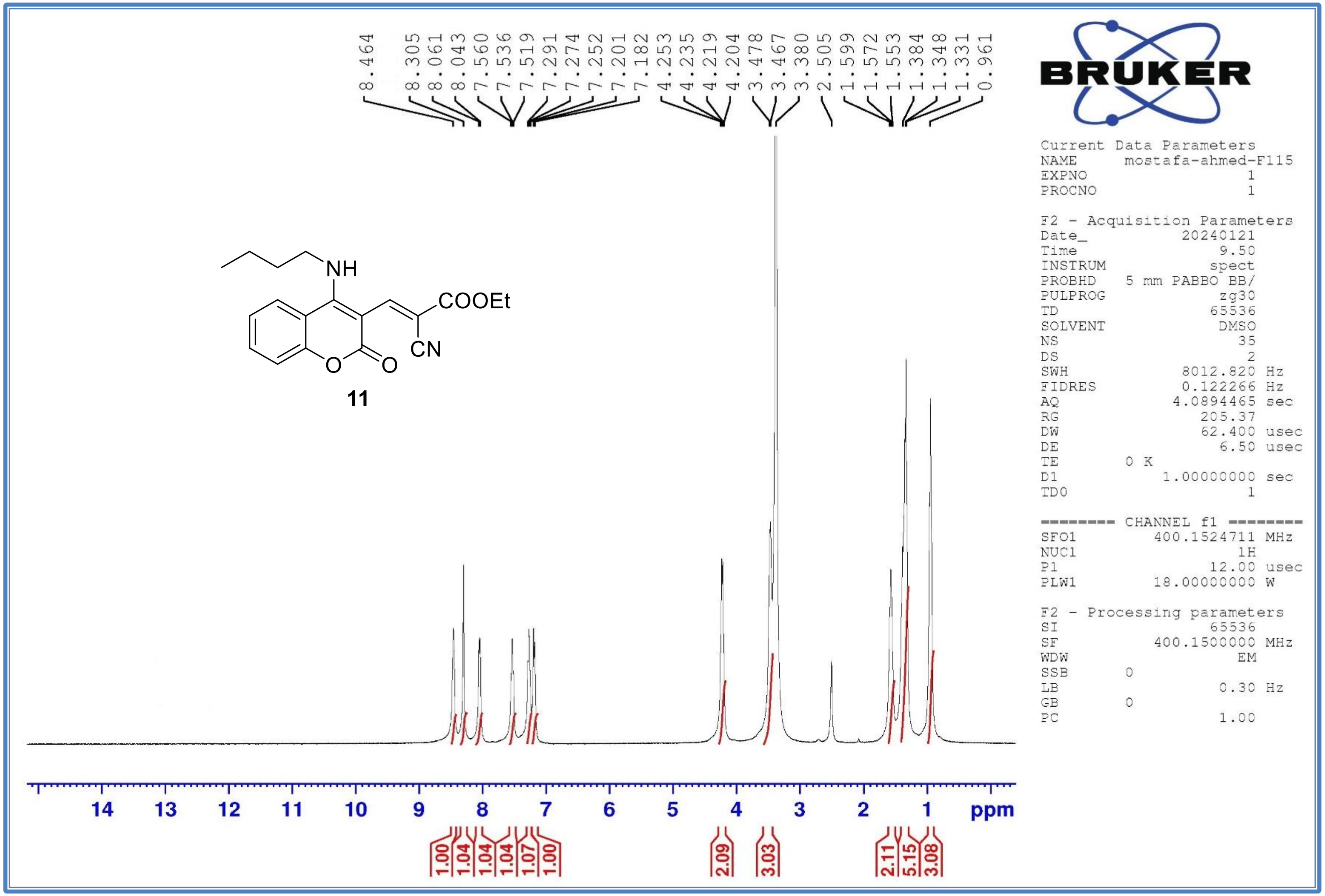

Spectroscopic data

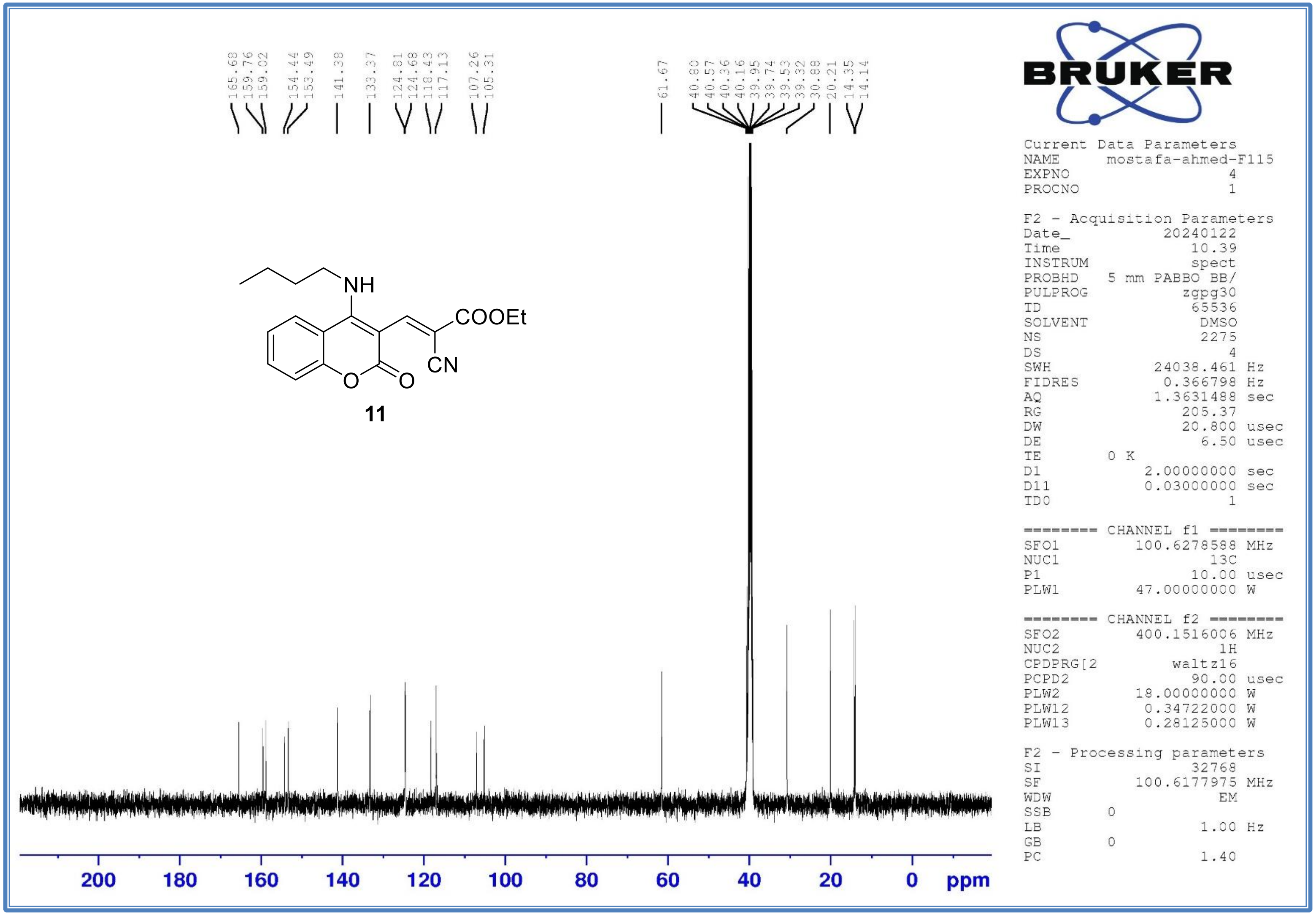

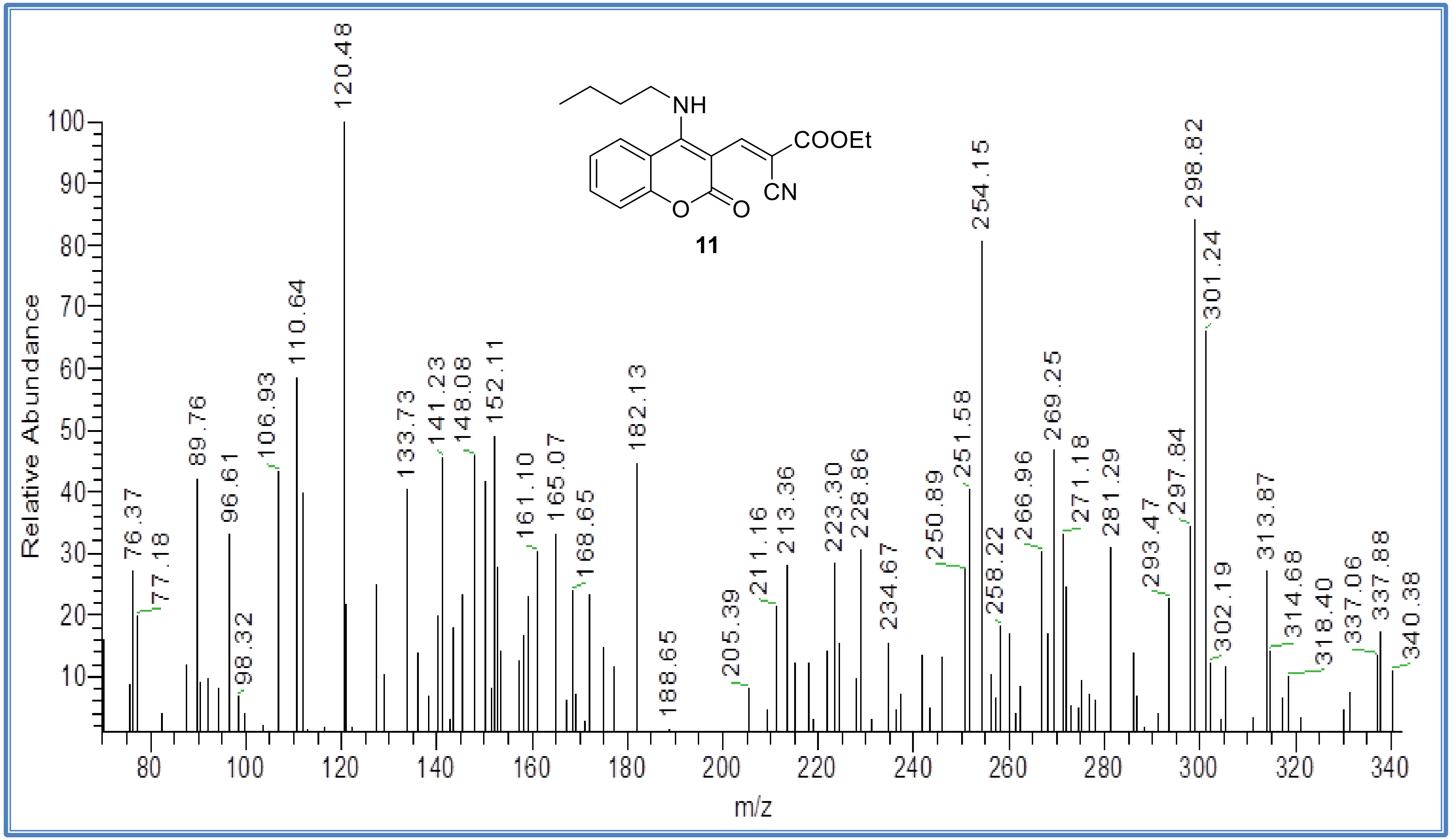
NH
COOEt
CN
O
O
11
Relative Abundance
m/z
120.48
110.64
106.93
89.76
96.61
98.32
76.37
77.18
133.73
141.23
148.08
152.11
161.10
165.07
168.65
182.13
188.65
205.39
211.16
213.36
223.30
228.86
234.67
250.89
251.58
254.15
258.22
266.96
269.25
271.18
281.29
293.47
297.84
298.82
301.24
302.19
313.87
314.68
318.40
337.06
337.88
340.38

Spectroscopic data

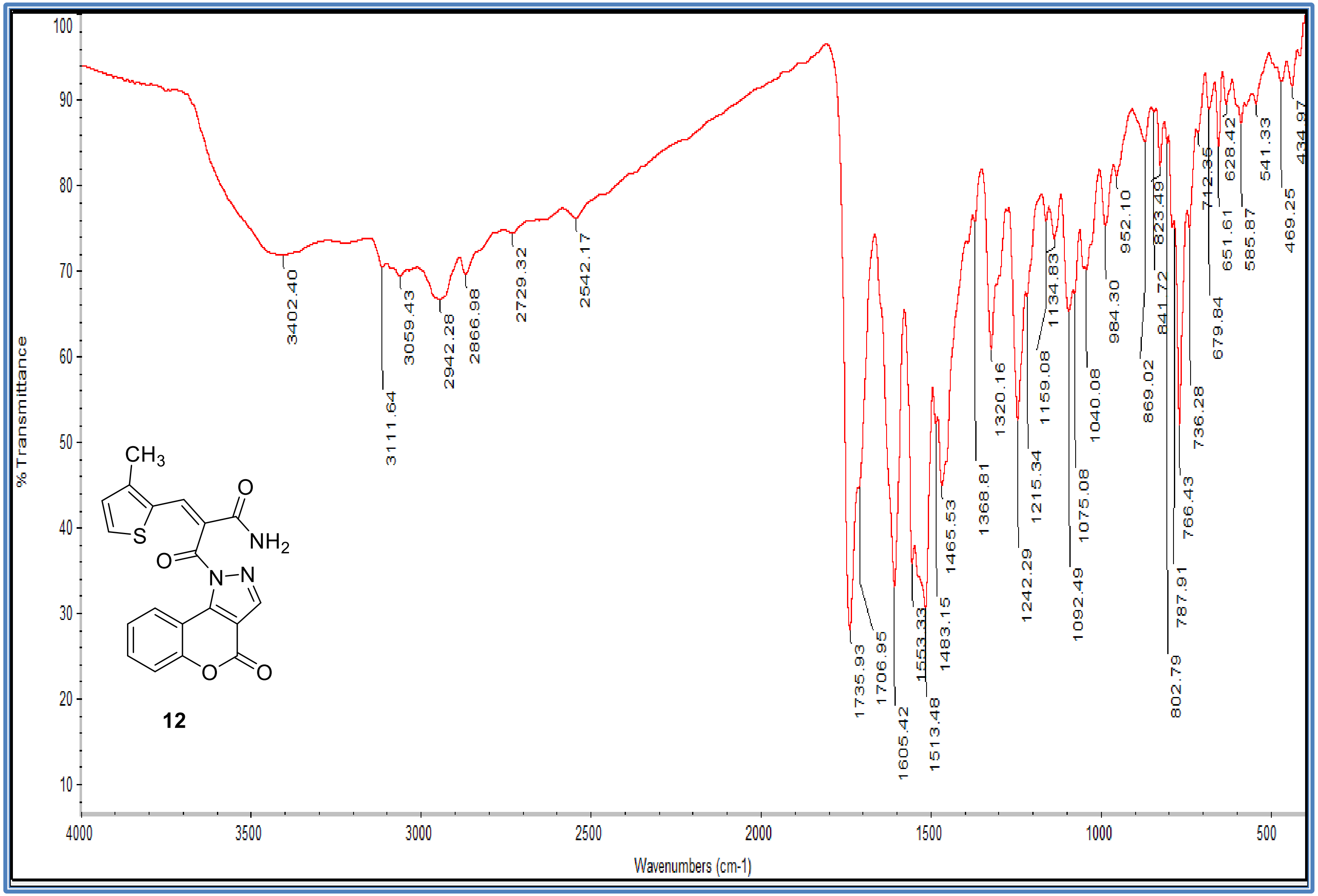

12

Spectroscopic data

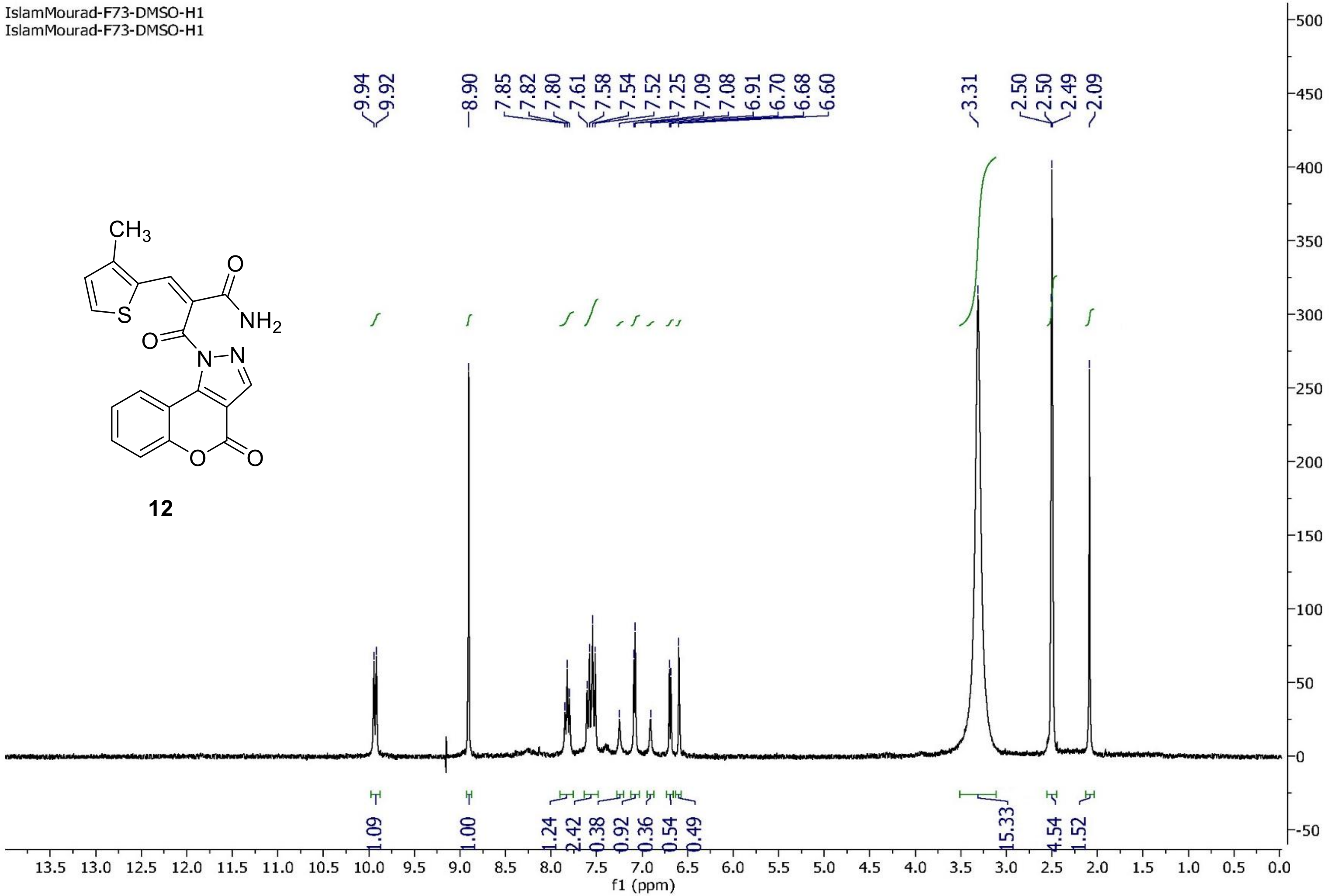

12

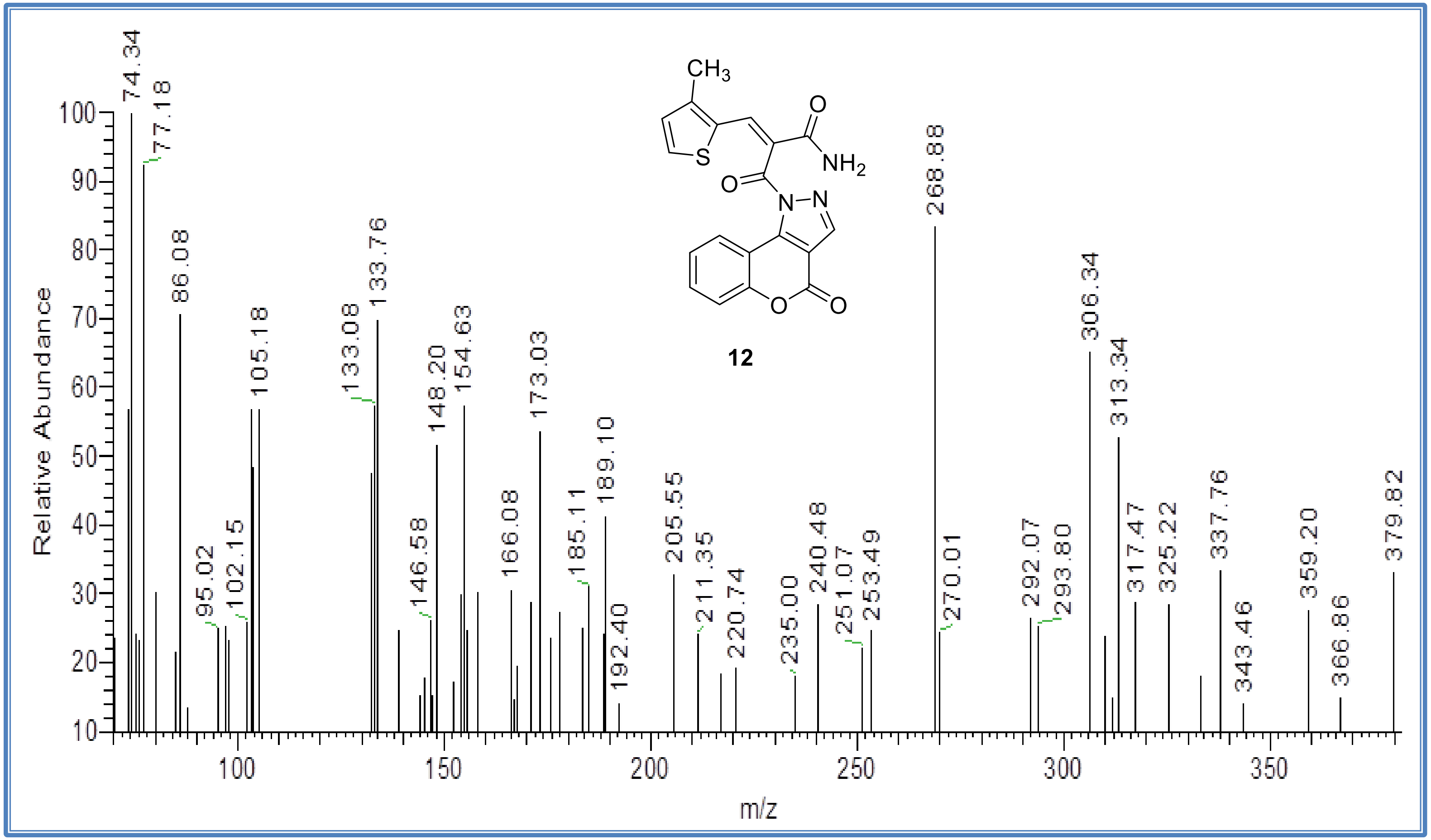
Relative Abundance
m/z
74.34
77.18
86.08
95.02
102.15
105.18
133.08
133.76
146.58
148.20
154.63
166.08
173.03
185.11
189.10
192.40
205.55
211.35
220.74
235.00
240.48
251.07
253.49
268.88
270.01
292.07
293.80
306.34
313.34
317.47
325.22
337.76
343.46
359.20
366.86
379.82
CH3
O
NH2
S
O
N
N
O
O
12

Spectroscopic data

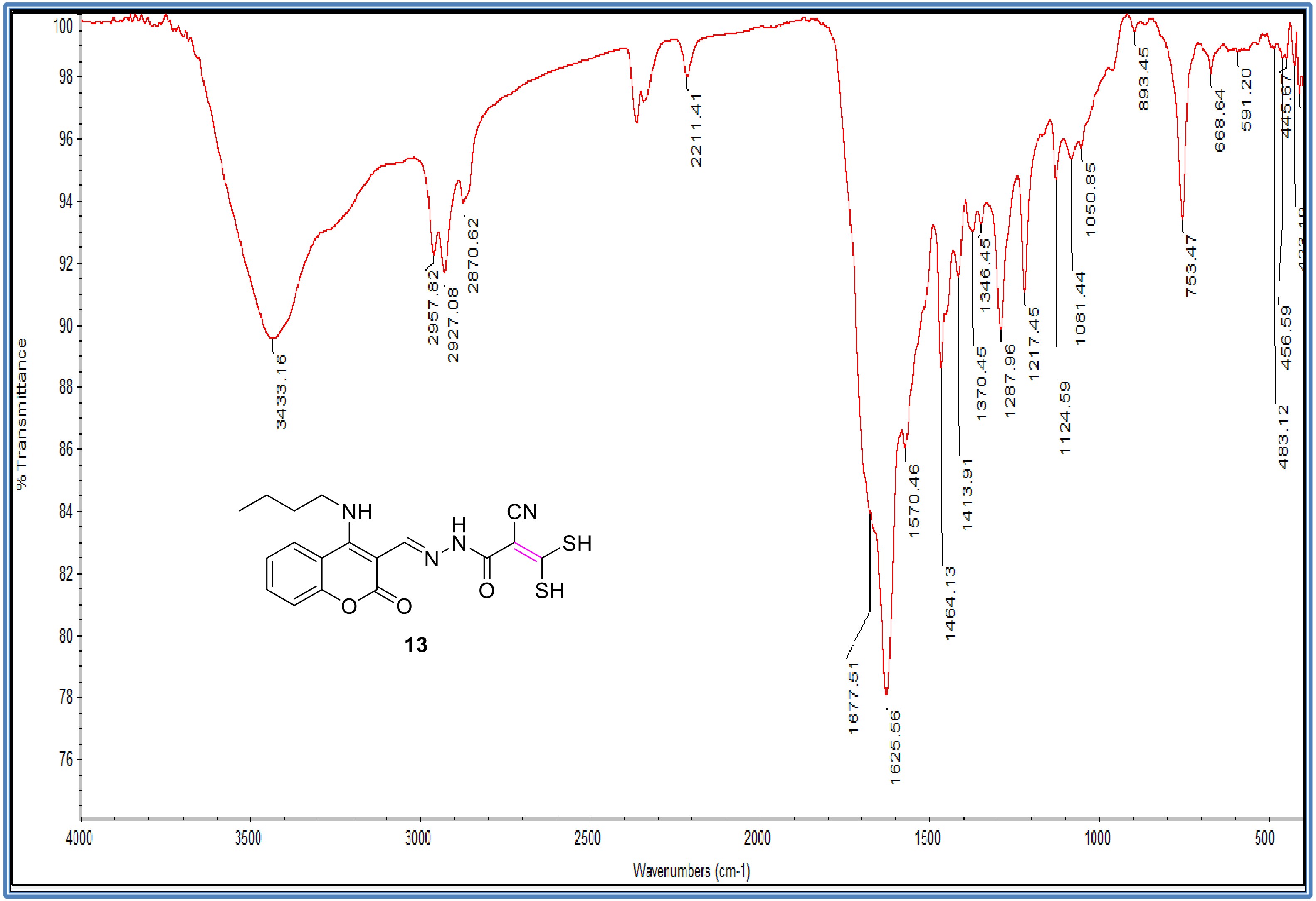

Spectroscopic data

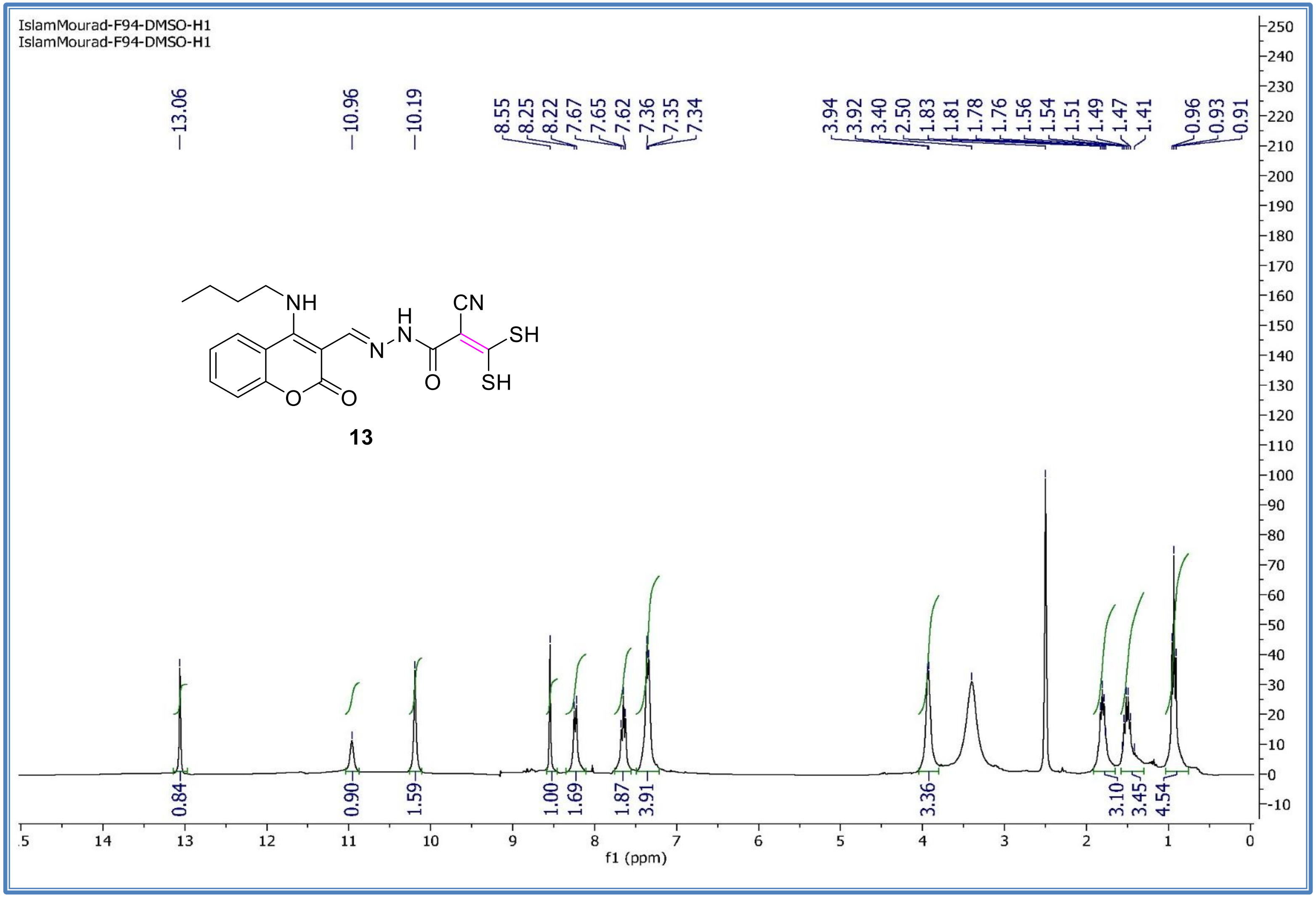

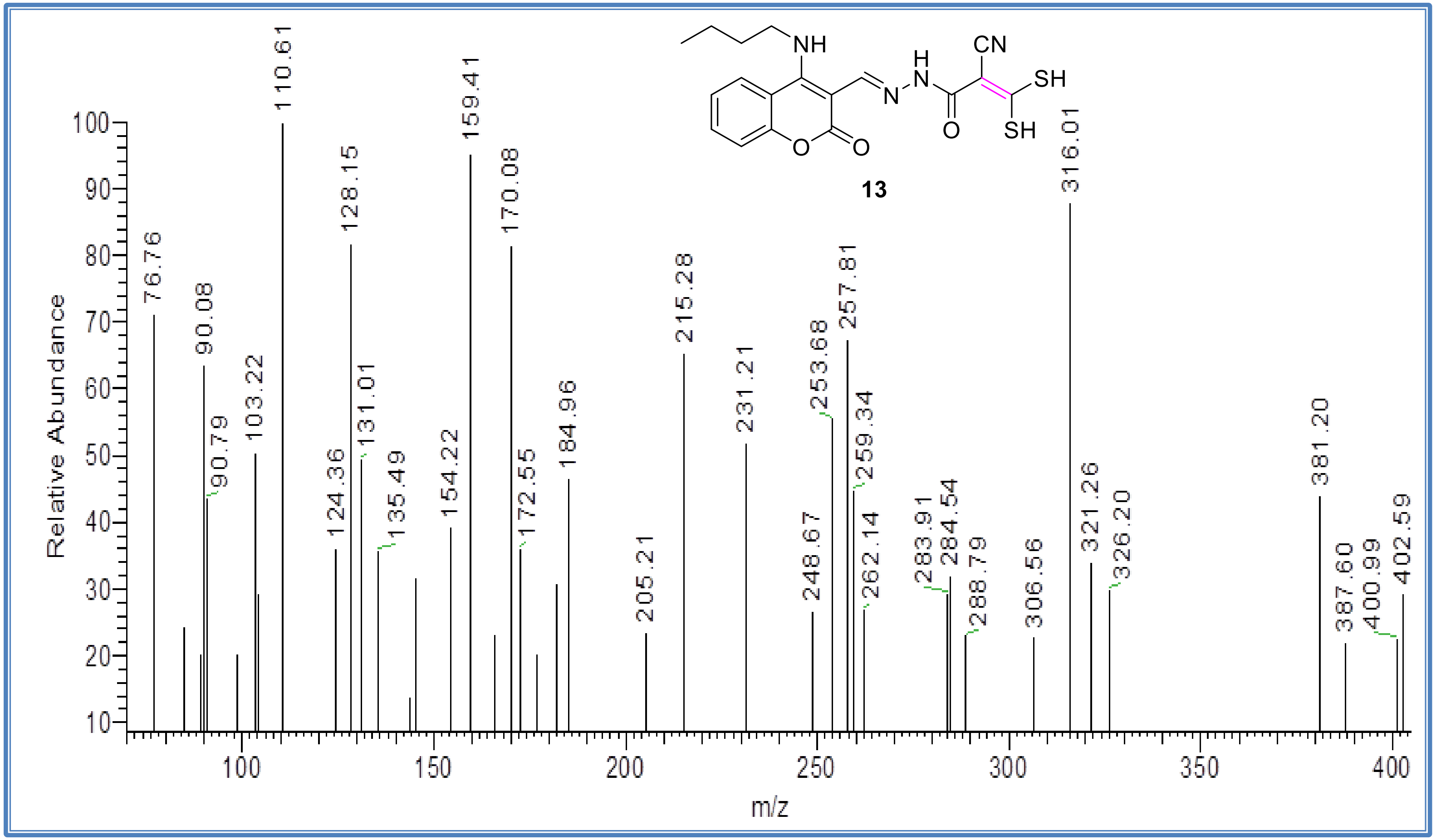
NH
H
N
N
CN
SH
SH
O
O
O
13
Relative Abundance
m/z
76.76
90.08
90.79
103.22
110.61
124.36
128.15
131.01
135.49
154.22
159.41
170.08
172.55
184.96
205.21
215.28
231.21
248.67
253.68
257.81
259.34
262.14
283.91
284.54
288.79
306.56
316.01
321.26
326.20
381.20
387.60
400.99
402.59

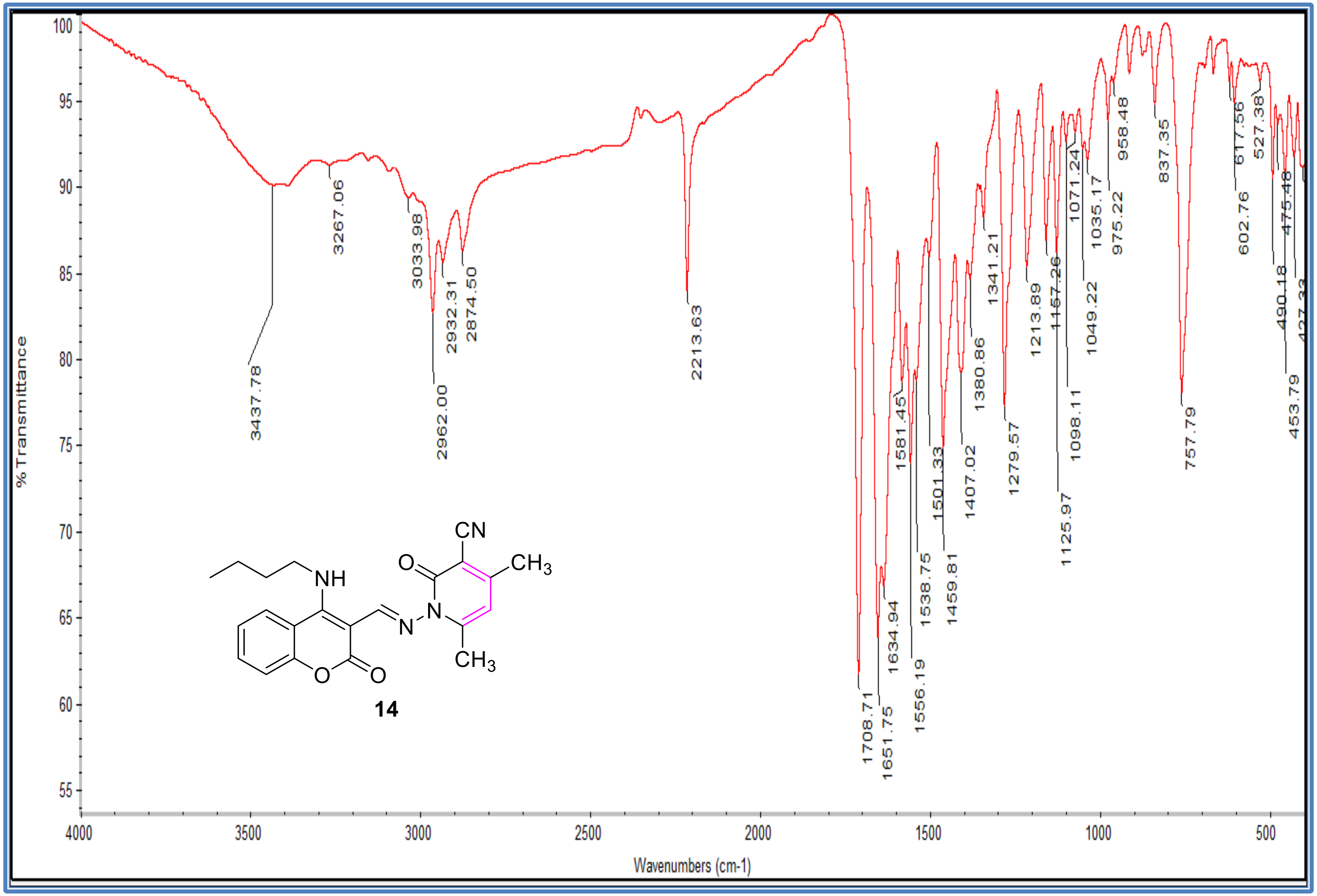

% Transmittance
100
95
90
85
80
75
70
65
60
55
Wavenumbers (cm-1)
4000
3500
3000
2500
2000
1500
1000
500
3437.78
3267.06
3033.98
2962.00
2932.31
2874.50
2213.63
1708.71
1651.75
1634.94
1581.45
1556.19
1538.75
1501.33
1459.81
1407.02
1380.86
1341.21
1279.57
1213.89
1157.26
1125.97
1098.11
1071.24
1049.22
1035.17
975.22
958.48
837.35
757.79
617.56
602.76
527.38
490.18
475.48
453.79
NH
CN
O
CH3
N
N
CH3
O
O
14

Spectroscopic data

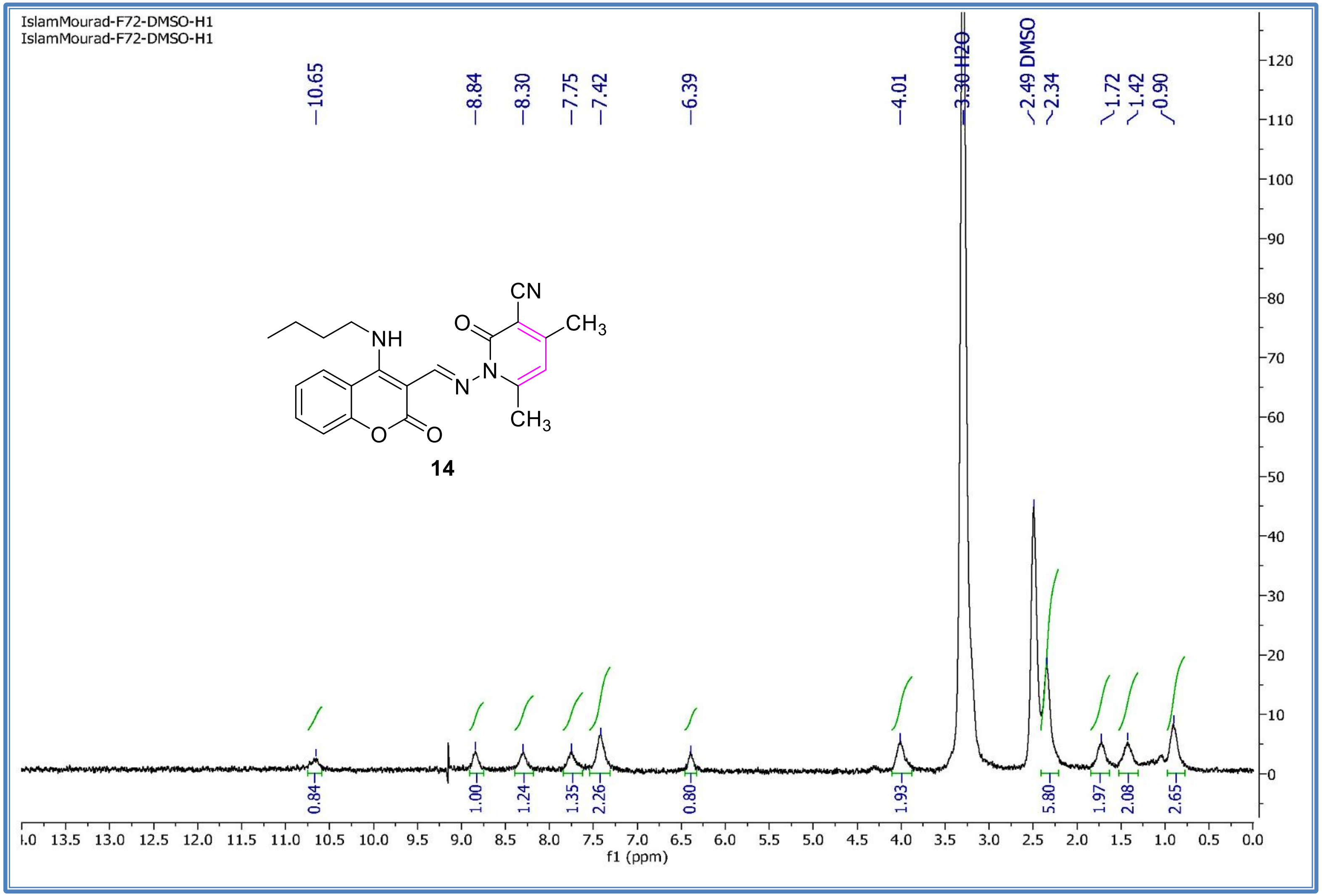

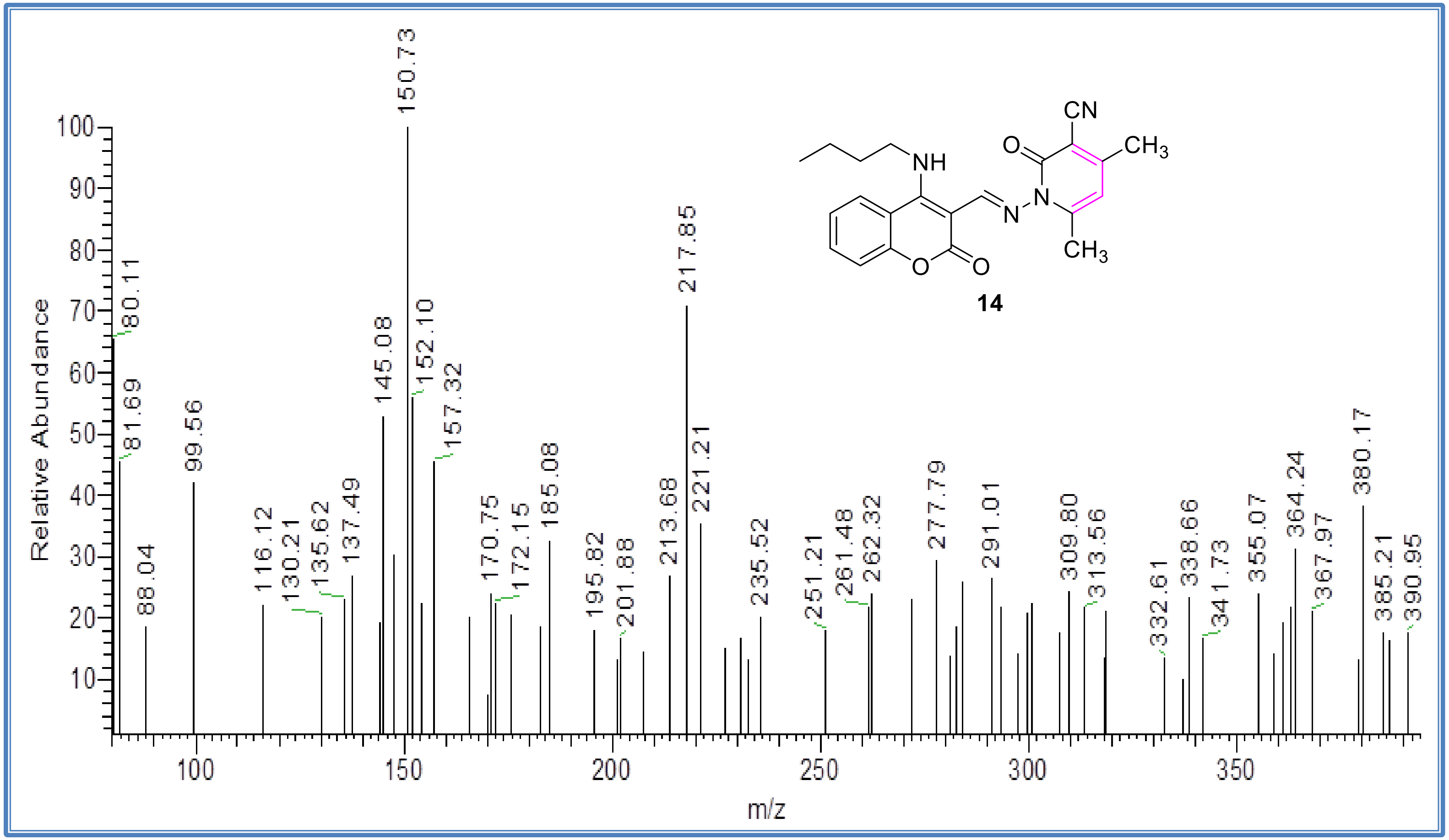

Relative Abundance
m/z
150.73
217.85
80.11
81.69
99.56
88.04
116.12
130.21
135.62
137.49
145.08
152.10
157.32
170.75
172.15
185.08
195.82
201.88
213.68
221.21
235.52
251.21
261.48
262.32
277.79
291.01
309.80
313.56
332.61
338.66
341.73
355.07
364.24
367.97
380.17
385.21
390.95
CN
CH3
NH
O
N
CH3
14

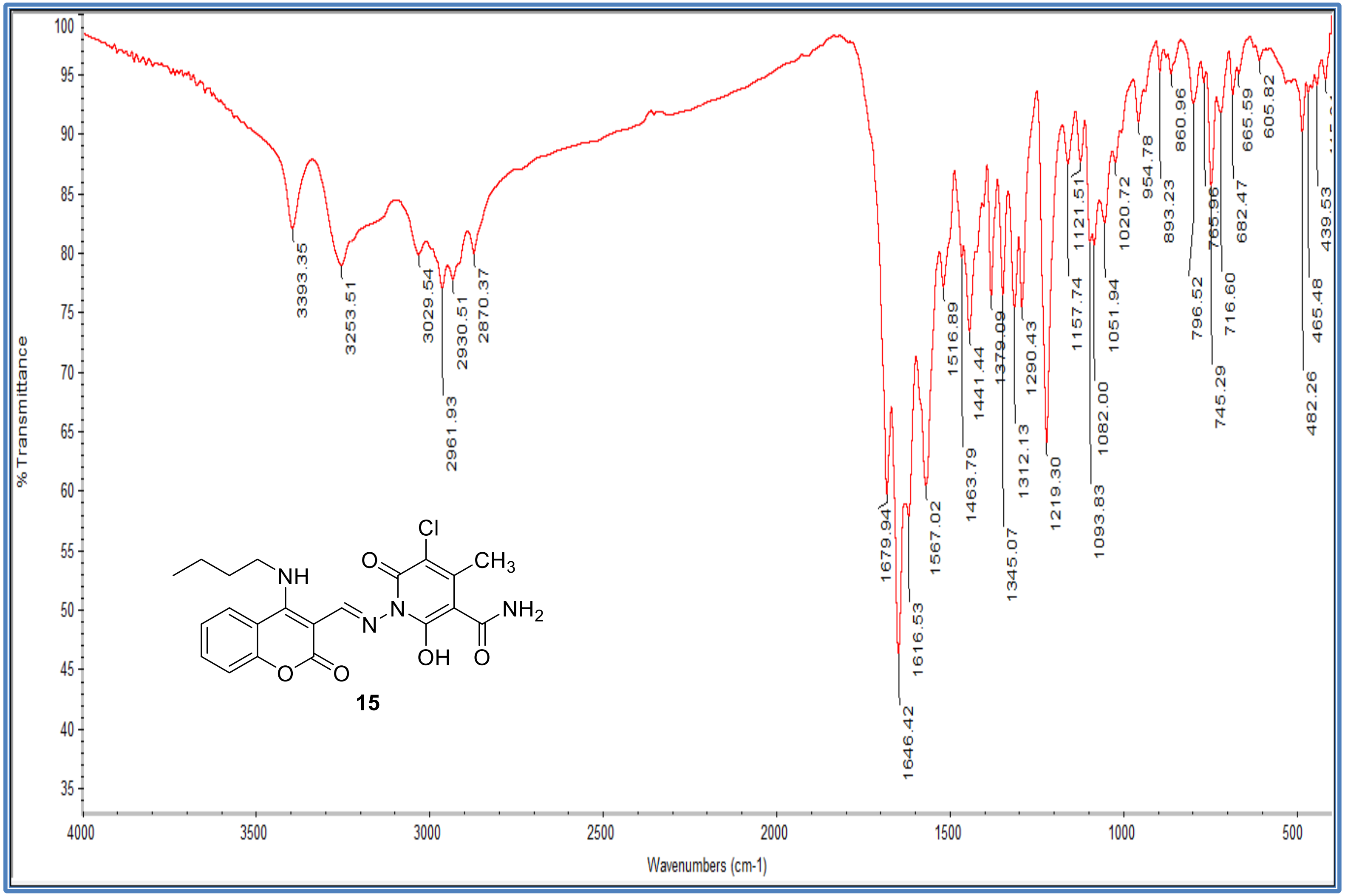

100
95
90
85
80
75
70
65
60
55
50
45
40
35
% Transmittance
4000
3500
3000
2500
2000
1500
1000
500
Wavenumbers (cm-1)
3393.35
3253.51
3029.54
2961.93
2930.51
2870.37
1679.94
1646.42
1616.53
1567.02
1516.89
1463.79
1441.44
1379.09
1345.07
1312.13
1290.43
1219.30
1157.74
1121.51
1093.83
1082.00
1051.94
1020.72
954.78
893.23
860.96
796.52
765.96
745.29
716.60
682.47
665.59
605.82
482.26
465.48
439.53
NH
Cl
CH3
NH2
N
OH
O
15

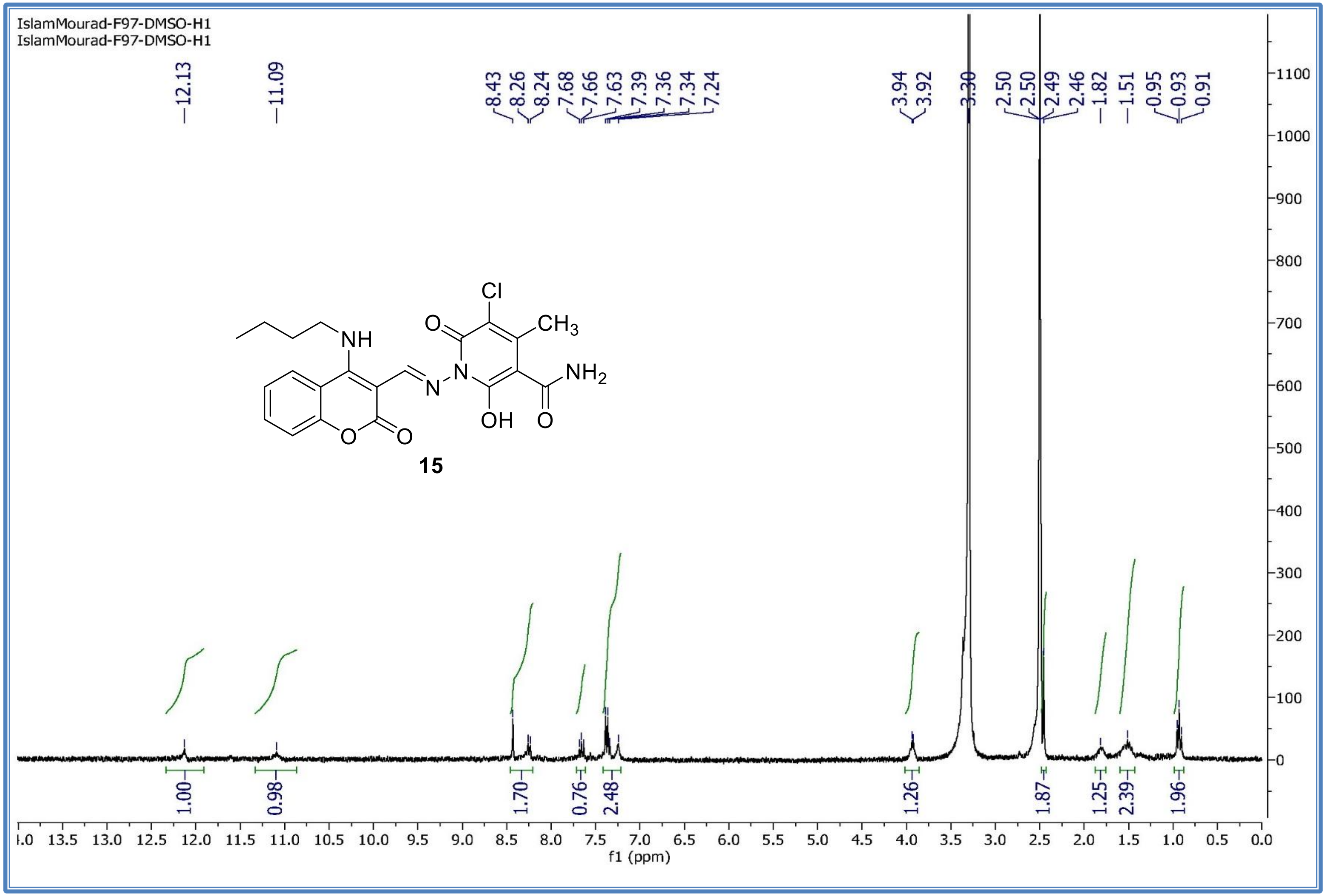
IslamMourad-F97-DMSO-H1
IslamMourad-F97-DMSO-H1
12.13
11.09
8.43 8.26 8.24 7.68 7.66 7.63 7.39 7.36 7.34 7.24
3.94 3.92
3.30
2.50 2.50 2.49 2.46
1.82
1.51
0.95 0.93 0.91
1.00 0.98 1.70 0.76 2.48 1.26 1.87 1.25 2.39 1.96
Cl
O
CH3
NH
NH2
N
OH
O
15
f1 (ppm)

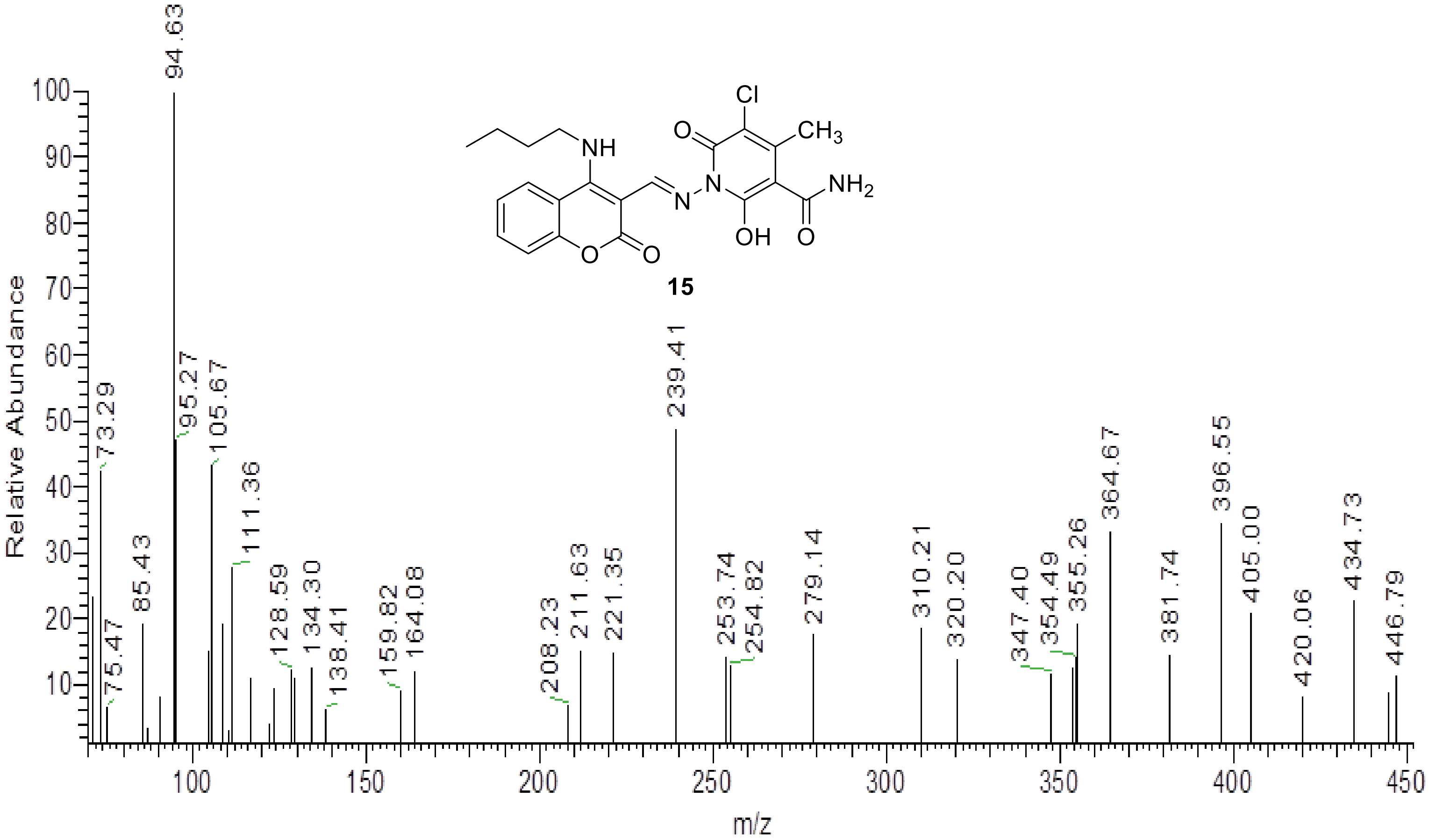

Relative Abundance
m/z
94.63
73.29
75.47
85.43
95.27
105.67
111.36
128.59
134.30
138.41
159.82
164.08
208.23
211.63
221.35
239.41
253.74
254.82
279.14
310.21
320.20
347.40
354.49
355.26
364.67
381.74
396.55
405.00
420.06
434.73
446.79
Cl
CH_3
O
NH
N
N
NH_2
OH
O
O
O
15

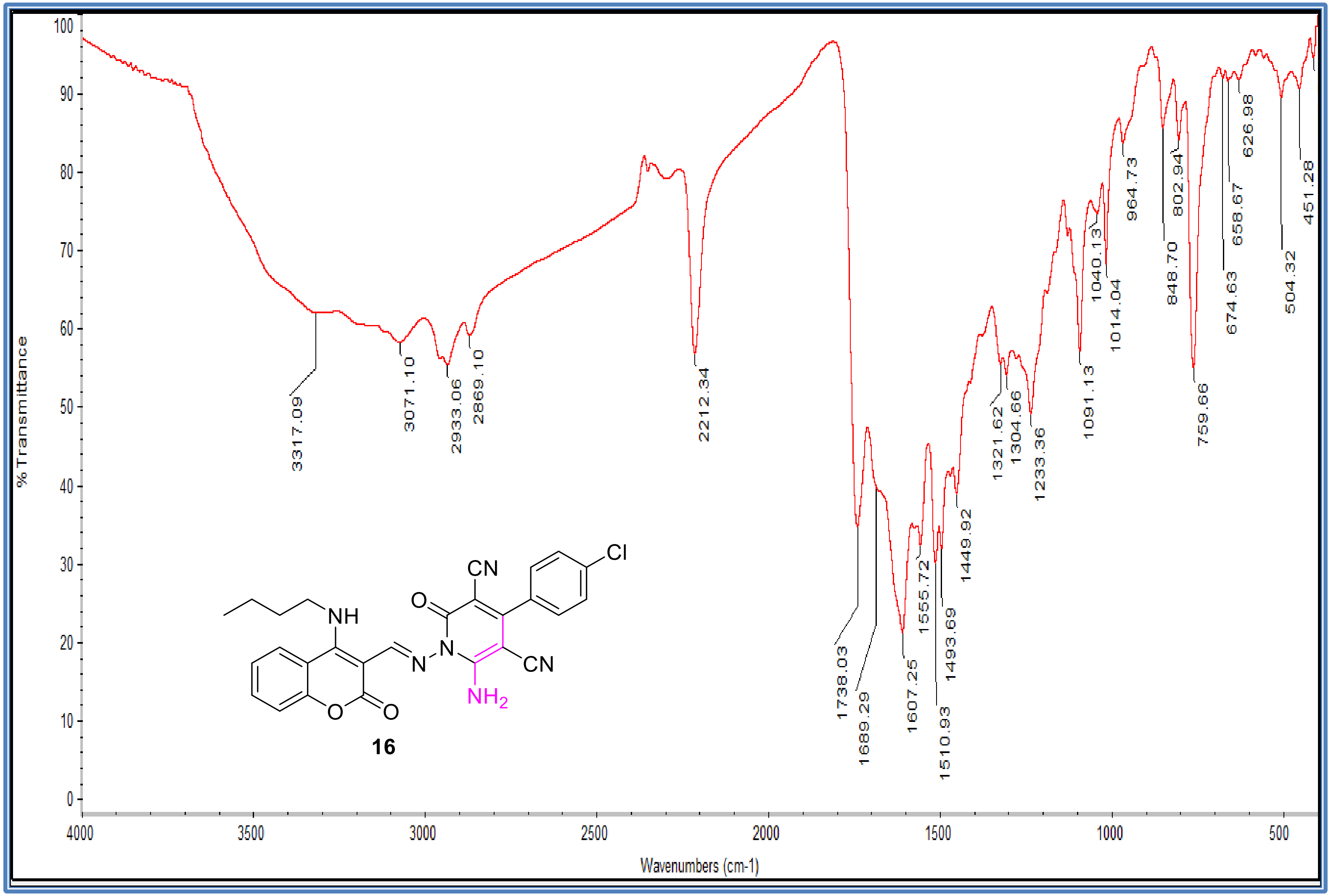
100
90
80
70
60
50
40
30
20
10
0
%Transmittance
4000
3500
3000
2500
2000
1500
1000
500
Wavenumbers (cm-1)
3317.09
3071.10
2933.06
2869.10
2212.34
1738.03
1689.29
1607.25
1555.72
1510.93
1493.69
1449.92
1321.62
1304.66
1233.36
1091.13
1040.13
1014.04
964.73
848.70
802.94
759.66
674.63
658.67
626.98
504.32
451.28
NH
CN
Cl
O
N
N
CN
NH2
O
O
16

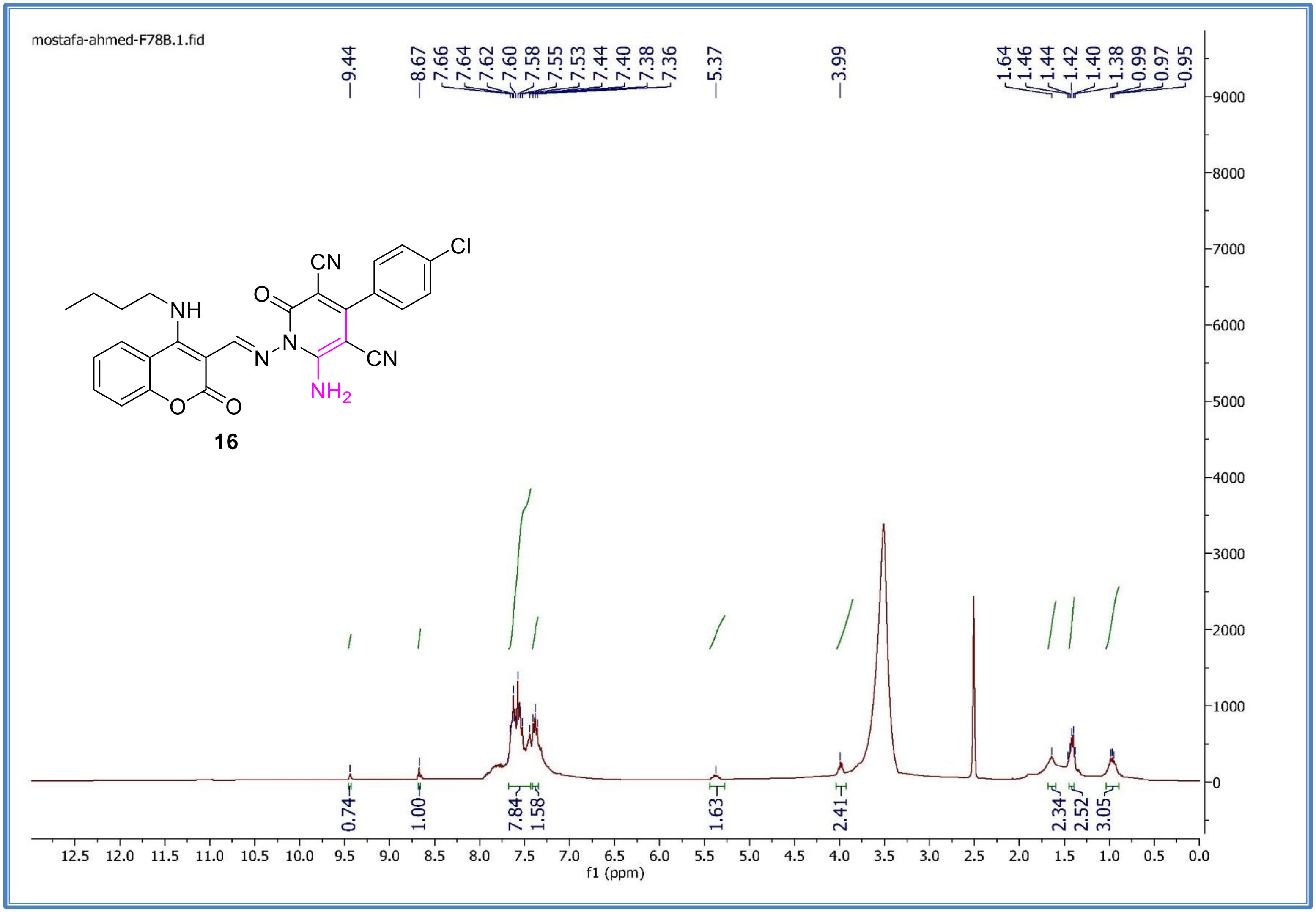
mostafa-ahmed-F78B.1.fid
9.44
8.67
7.66
7.64
7.62
7.60
7.58
7.55
7.53
7.44
7.40
7.38
7.36
5.37
3.99
1.64
1.46
1.44
1.42
1.40
1.38
0.99
0.97
0.95
CN
Cl
O
NH
N
CN
N
O
O
NH2
16
0.74
1.00
7.84
1.58
1.63
2.41
2.34
2.52
3.05
f1 (ppm)

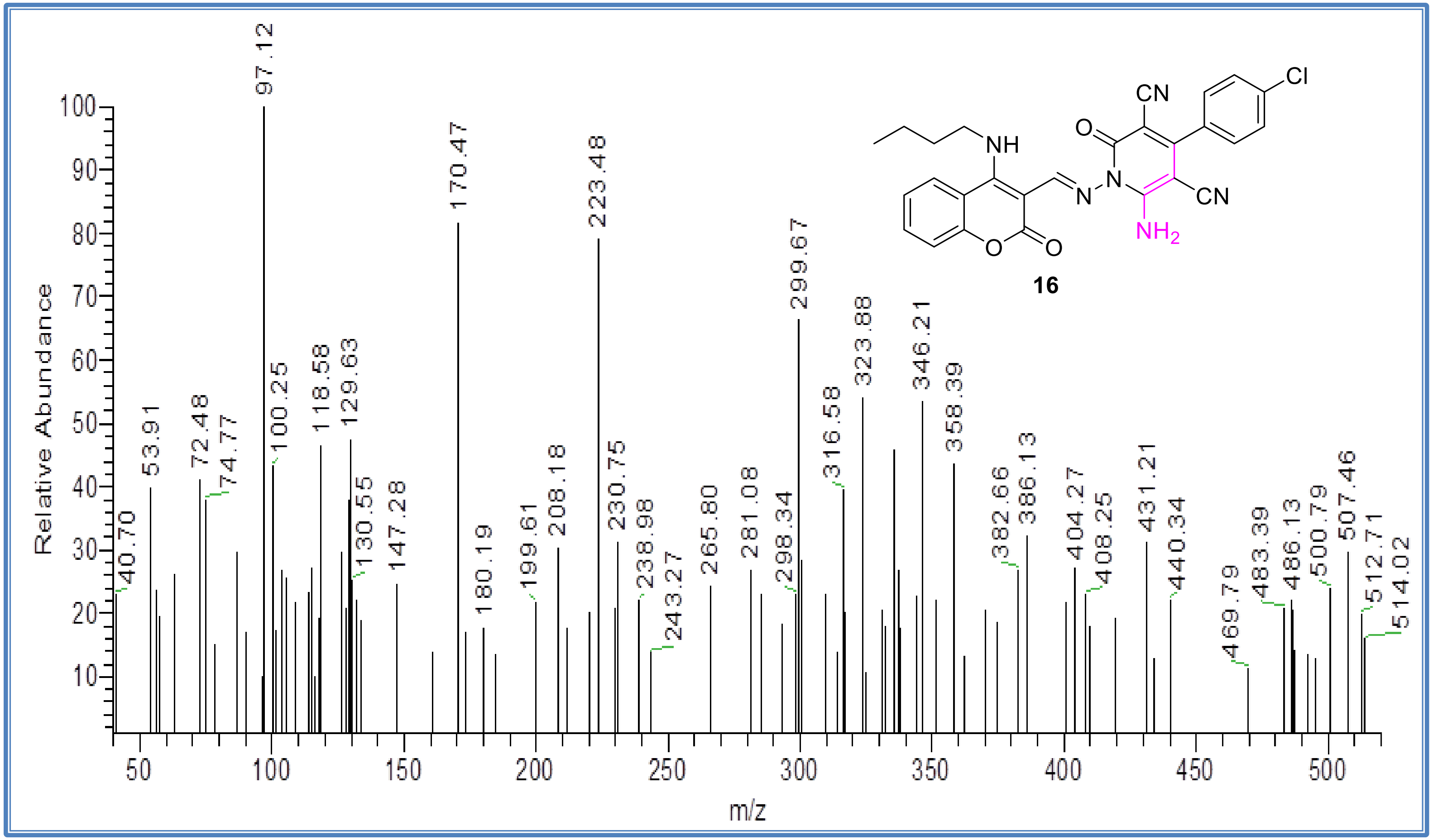

97.12
170.47
223.48
299.67
323.88
346.21
358.39
316.58
100.25
118.58
129.63
53.91
72.48
74.77
40.70
130.55
147.28
180.19
199.61
208.18
230.75
238.98
243.27
265.80
281.08
298.34
382.66
386.13
404.27
408.25
431.21
440.34
469.79
483.39
486.13
500.79
507.46
512.71
514.02
Relative Abundance
m/z
NH
CN
Cl
O
N
CN
NH2
O
O
16

Spectroscopic data

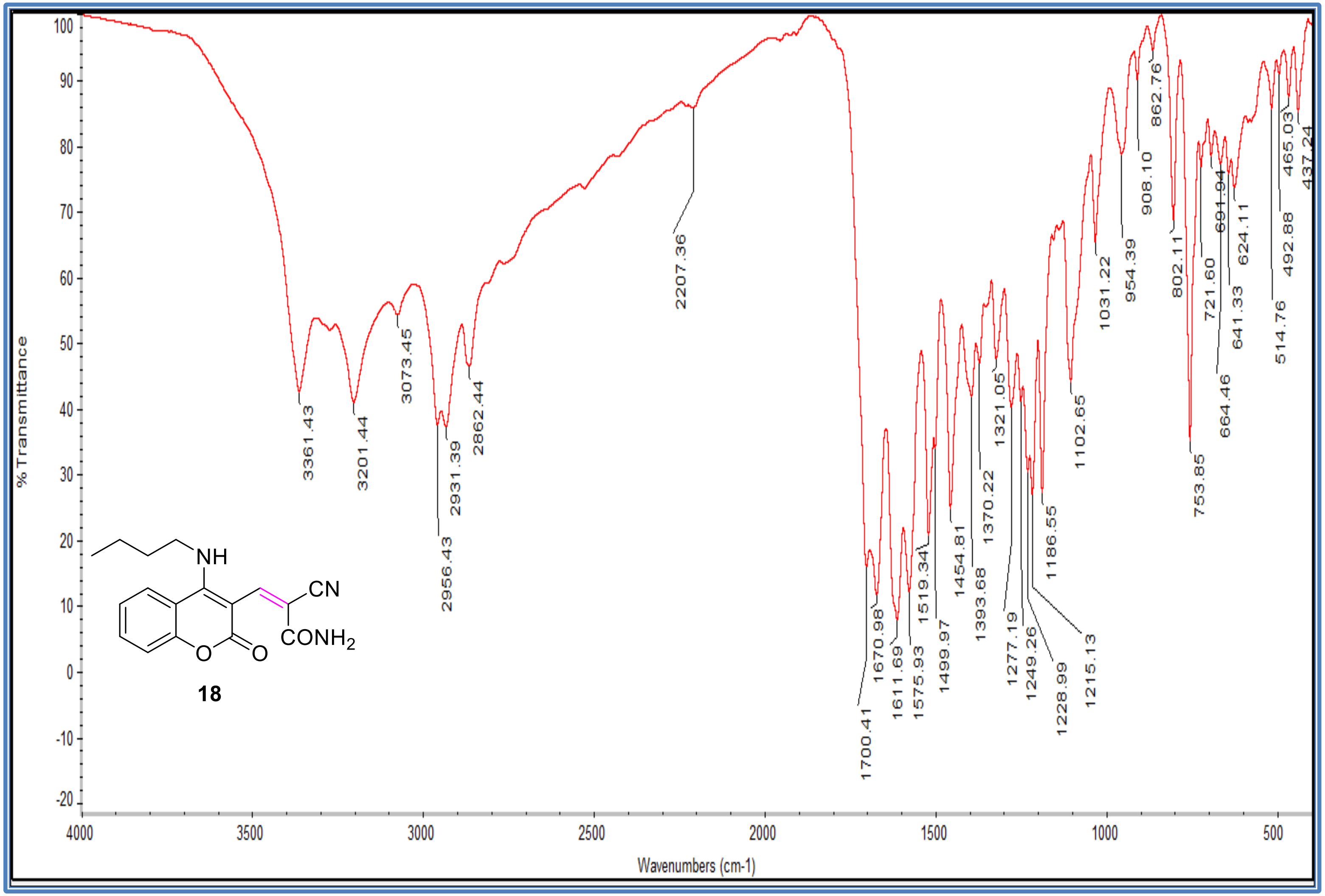

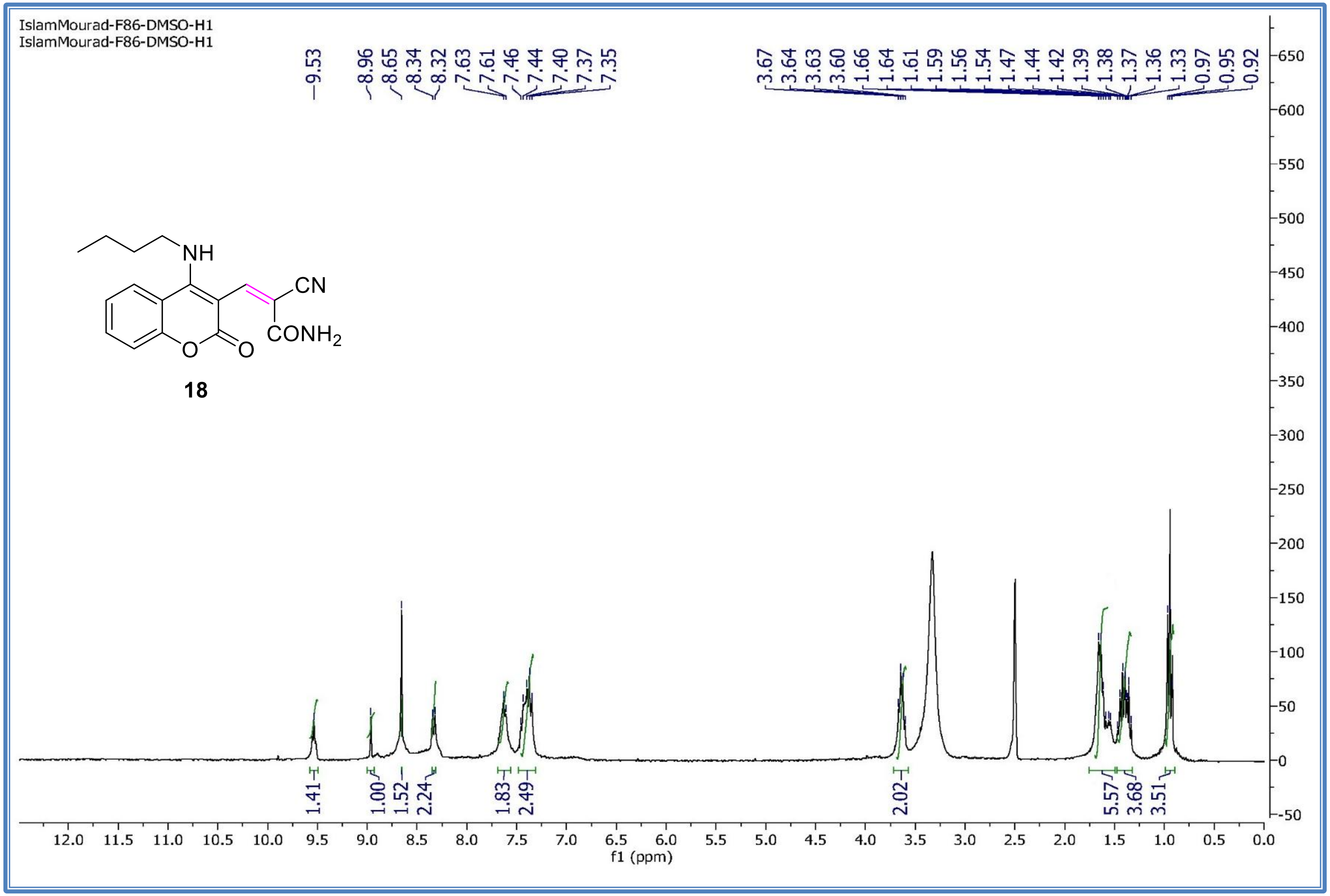
IslamMourad-F86-DMSO-H1
IslamMourad-F86-DMSO-H1
9.53
8.96
8.65
8.34
8.32
7.63
7.61
7.46
7.44
7.40
7.37
7.35
3.67
3.64
3.63
3.60
1.66
1.64
1.61
1.59
1.56
1.54
1.47
1.44
1.42
1.39
1.38
1.37
1.36
1.33
0.97
0.95
0.92
NH
CN
CONH2
O
O
18
1.41
1.00
1.52
2.24
1.83
2.49
2.02
5.57
3.68
3.51
f1 (ppm)

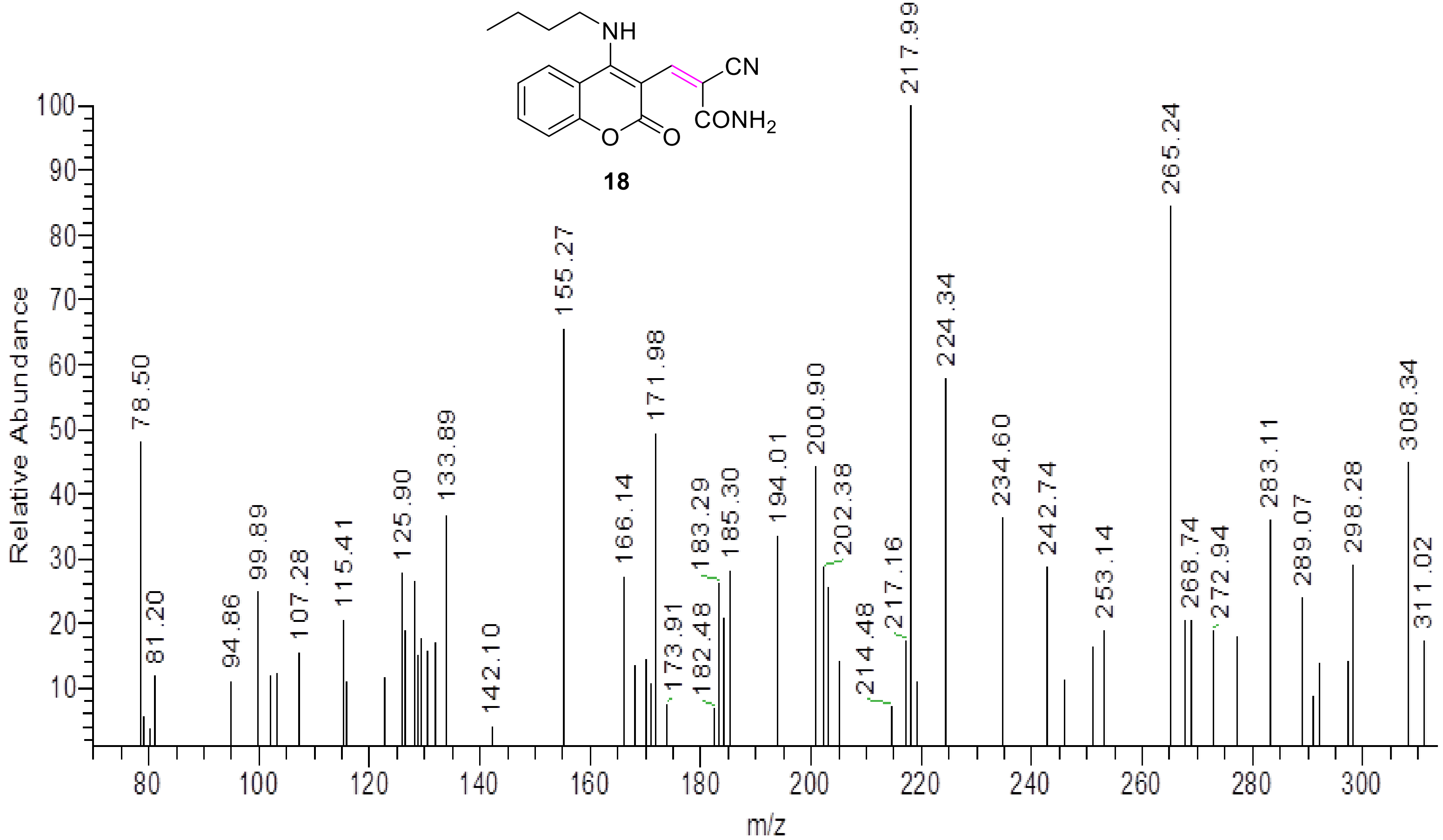
NH
CN
CONH2
O
O
18
Relative Abundance
m/z
217.99
265.24
155.27
224.34
171.98
200.90
78.50
308.34
133.89
234.60
283.11
194.01
99.89
125.90
202.38
185.30
183.29
242.74
298.28
166.14
115.41
107.28
217.16
311.02
253.14
289.07
268.74
272.94
81.20
94.86
214.48
182.48
173.91
142.10

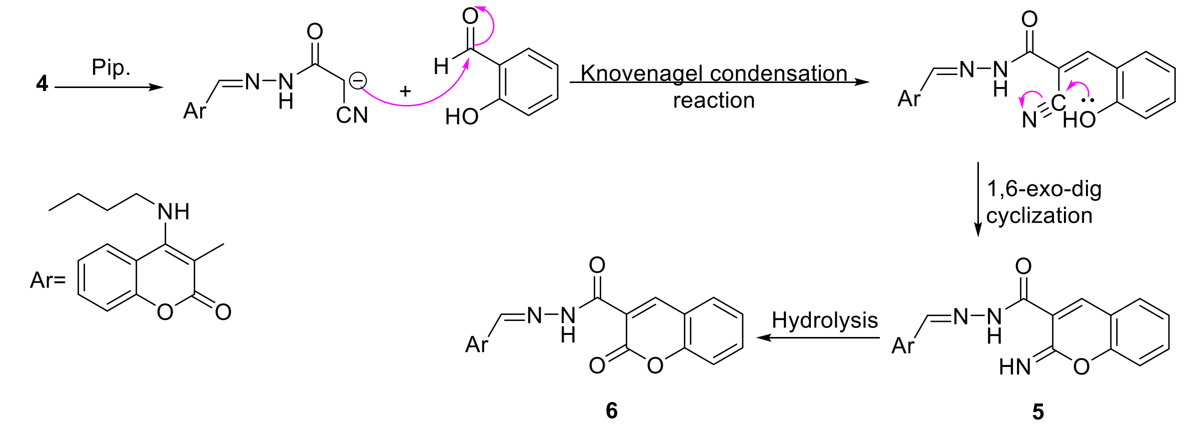

Scheme 1: The plausible mechanisms for formation of compounds 5 and 6.

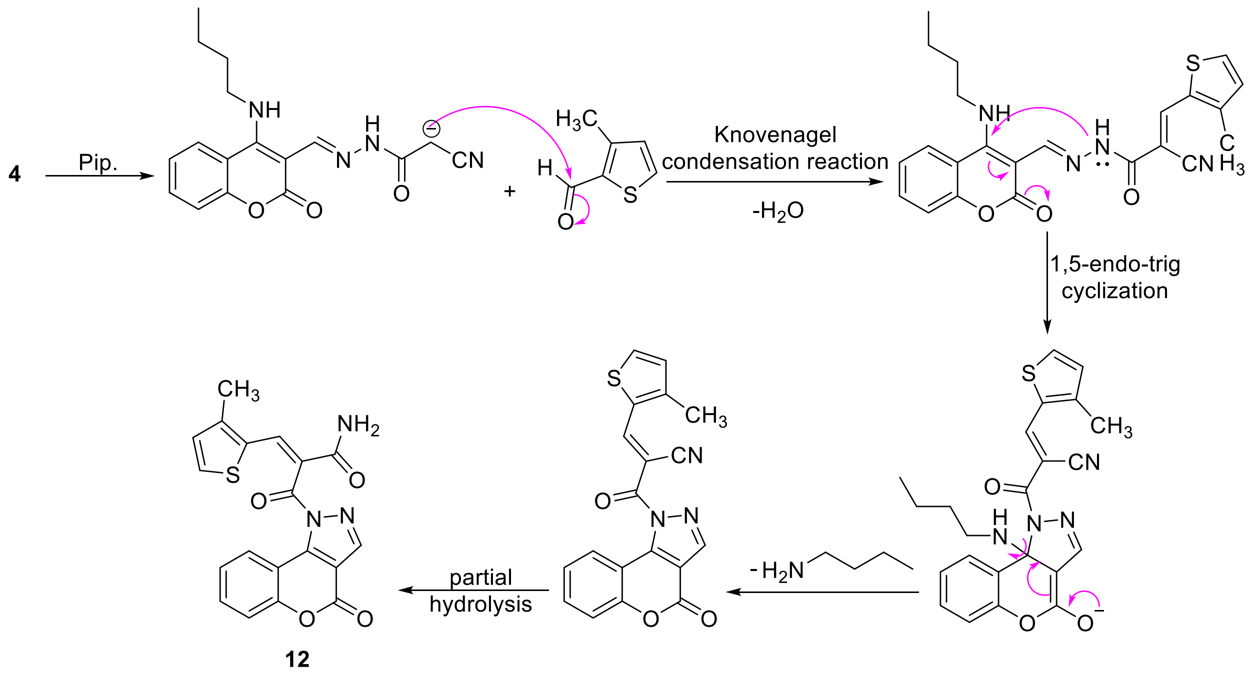

Scheme 2: The plausible mechanisms for formation of compound 12.
